# Cu(I)/Pd(II)-Catalyzed Intramolecular Hydroamidation and C-H Dehydrogenative Coupling of *ortho*-Alkynyl-*N*-arylbenzamides for Access to Isoindolo[2,1-*a*]Indol-6-Ones

**DOI:** 10.3390/molecules27113393

**Published:** 2022-05-25

**Authors:** Baoxin Tang, Ruimao Hua

**Affiliations:** 1 Department of Chemistry, Tsinghua University, Key Laboratory of Organic Optoelectronics & Molecular Engineering of Ministry of Education, Beijing 100084, China; 13261757195@163.com; 2 College of Chemistry, Xinjiang University, Urumqi 830017, China

**Keywords:** *ortho*-alkynyl-*N*-arylbenzamide, C-H dehydrogenative coupling, hydroamidation of alkyne, isoindolo[2,1-*a*]indol-6-one

## Abstract

An efficient, atom-economic and one-pot synthesis of isoindolo[2,1-*a*]indol-6-ones via CuI/Pd(OAc)_2_-catalyzed intramolecular hydroamidation of alkynyl group, and C-H dehydrogenative coupling of *ortho*-alkynyl-*N*-arylbenzamides has been developed. This transformation occurs with the use of oxygen as the oxidant, and water is the only by-product. The reaction shows a high tolerance to a variety of functional groups, and affords isoindolo[2,1-*a*]indol-6-ones in good to high yields.

## 1. Introduction

**Figure 1:**
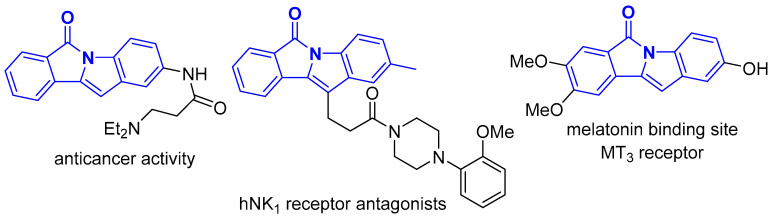
Representative structures of 6*H*-isoindolo[2,1-*a*]-indol-6-ones.

Indole alkaloids are the important class of nitrogen-heterocyclic compounds with interesting physiological and biological activities in natural products, medicines, and agrochemicals [1,2,3]. Among them, 6*H*-isoindolo[2,1-*a*]indol-6-ones, a tetracyclic fused indole and isoindoline ring system, have received increasing attention due to their highly potential application with anticancer activity [4,5], affinity for hNK1 receptor [6], affinity for the melatonin binding site MT_3_ [7] (Figure 1), and used as a precursor for the synthesis of NorA pump inhibitors [8,9]. Therefore, there has been increasing interest in the developments of the efficient methodologies for the synthesis of 6*H*-isoindolo[2,1-*a*]indol-6-ones [10].

**Scheme 1:**
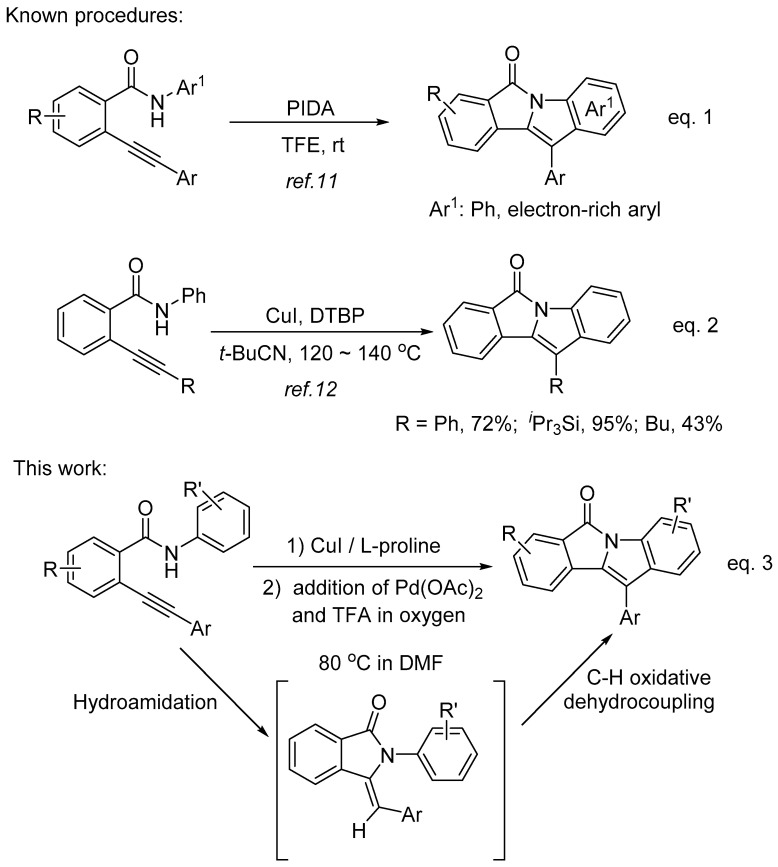
6*H*-Isoindolo[2,1-*a*]indol-6-ones from *ortho*-alkynyl-*N*-arylbenzamides.

In the reported procedures, there are two reaction systems for the synthesis of 6*H*-isoindolo[2,1-*a*]indol-6-ones starting from *ortho*-alkynyl-*N*-arylbenzamides (Figure 1) [11,12]. One is the hypervalent iodine, PhI(OAc)_2_ (PIDA)-mediated intramolecular cascade oxidative cyclization of *ortho*-(1-arylethynyl)-*N*-arylbenzamides affording 11-aryl-6*H*-isoindolo[2,1-*a*]indol-6-ones at room temperature in trifluoroethanol (TFE), but Ar^1^ groups are limited to phenyl and electron-rich aryl groups (Scheme 1, Equation (1)) [11]. The other is CuI-catalyzed radical cascade cyclization of *ortho*-alkynyl-*N*-arylbenzamides in the presence of di-*t*-butyl peroxide (DTBP) in *t*-BuCN at 120~140 °C giving three products of 6*H*-isoindolo[2,1-*a*]indol-6-ones (Scheme 1, Equation (2)) [12].

In recent years, we have been interested in the applications of *ortho*-alkynyl aromatic aldehydes/ketones [13] and functionalized benzamides [14,15] in the synthesis of hetero-/carbo-cyclic compounds. Therefore, in continuation of our interests in development of synthetic methods for the synthesis of nitrogen-heterocyclic compounds via alkyne annulations [16,17,18], we herein describe a CuI/Pd(OAc)_2_-catalyzed formation of 11-aryl-6*H*-isoindolo-[2,1-*a*]-indol-6-ones bearing different functional group(s) from *ortho*-(1-arylethynyl)-*N*-arylbenzamides by the stepwise hydroamidation of the alkynyl group [19], and C-H dehydrogenative coupling reaction [20] with the use of O_2_ as the oxidant (Scheme 1, Equation (3)).

## 2. Results and Discussion

As the designed reaction shows in Scheme 1, the construction of 6*H*-isoindolo-[2,1-*a*]indol-6-ones from *ortho*-(1-arylethynyl)-*N*-arylbenzamides includes the hydroamidation of alkynyl group, and the C-H oxidative dehydrocoupling reaction. Our initial studies focused on optimizing the reaction conditions for the intramolecular hydroamidation of *ortho*-(1-arylethynyl)-*N*-arylbenzamides (**1a**) under N_2_ atmosphere at 80 °C with the use of different catalysts, solvents, and additives (Table 1). When FeCl_3_, Cu(OAc)_2_, Pd(OAc)_2_, and CuI were used as the catalysts (entries 1–4), CuI shows the highest catalytic activity in DMF to catalyze the hydroamidation giving 38% yield of (*Z*)-3-benzylidene-2-phenylisoindolin-1-one (**2a**), which is a known compound, and its X-ray diffraction studies confirm the structure unambiguously [21]. The use of DMSO, MeCN, NMP, 1,4-dioxane, and toluene as the solvents to replace DMF resulted in the decrease of yields (entries 5–9). The addition of nitrogen-containing ligands could improve the catalytic activity of CuI in DMF, and the yield was up to 92% with the use of L-proline (entries 10–12). In addition, it has been found that CuCl and CuBr prove less effective in DMF with the use of L-proline as the ligand (entries 13–14).

After the reaction was finished under the conditions indicated in entry 12 of Table 1, a catalytic amount of Pd(OAc)_2_ (5 mol %) was directly added to the reaction mixture to promote the oxidative C-H dehydrogenative coupling reaction with the use of different oxidants and additional solvent. As shown in Table 2, with the addition of HOAc as the additional solvent, and oxygen (1 atm), 1,4-benzoquinone, K_2_S_2_O_8_, or Cu(OAc)_2_ as the oxidants, the desired 11-phenyl-6*H*-isoindolo- [2,1-*a*]indol-6-one (**2aaa**) was formed, and the green oxidant oxygen exhibits the best result (entries 1–4). The use of *p*-TsOH and TFA to replace HOAc could significantly improve the yields of **2aaa** (entries 5–6). These results could be explained by the AcO^-^ group being replaced by TsO^-^ or CF_3_COO^-^ groups to increase the electrophilicity of the Pd(II) center to enhance the catalytic activity for activation of aromatic C-H bond [22,23]. However, either increasing or decreasing the reaction temperature could not further improve the yield of **2aaa** (entries 7–8).

**Scheme 2:**
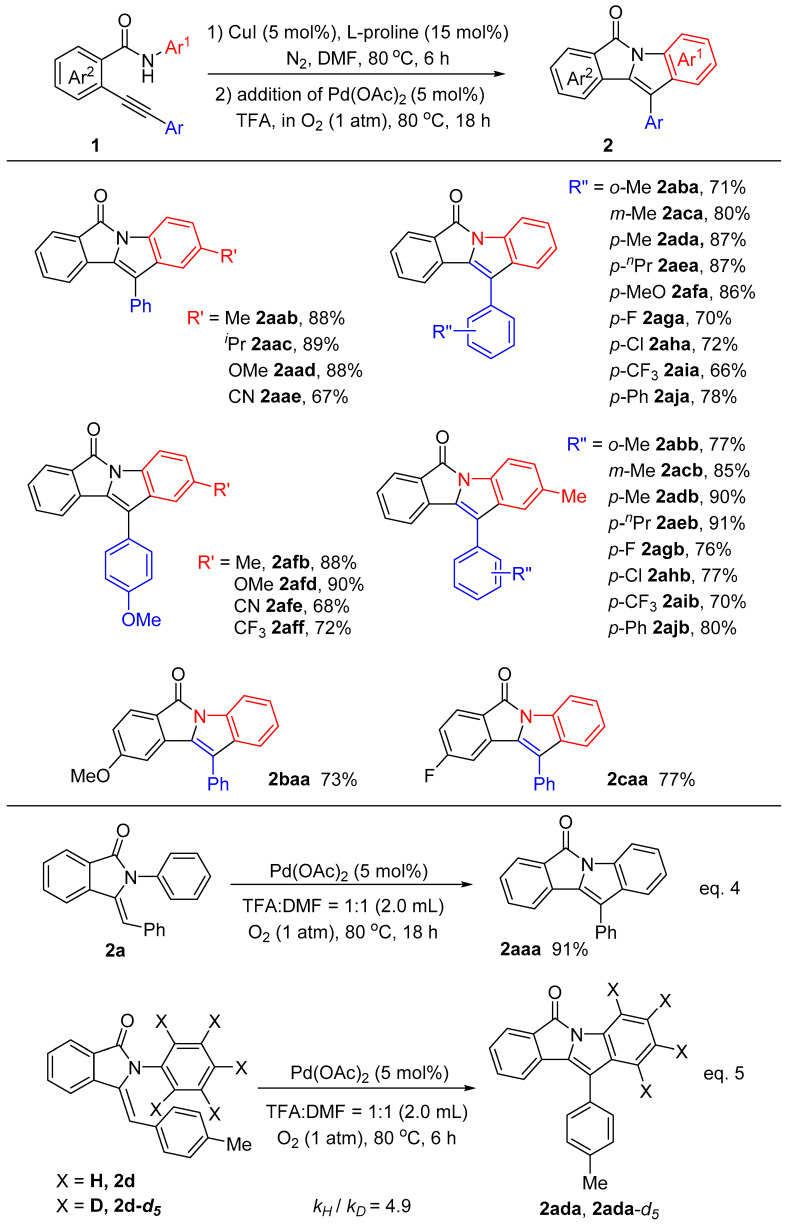
Substrate scope of isoindolo[2,1-*a*]indol-6-one synthesis.

The combined reaction conditions shown in entry 12 of Table 1 and entry 6 of Table 2 were then used to investigate the scope and generality of isoindolo[2,1-*a*]indol-6-one formation with the use of various substrates bearing electron-donating and electron-withdrawing groups in the aromatic rings. Based on the results shown in Scheme 2, several features of the relationship between electron effect or steric effect and yields can be concluded: (1) Substrates with either electron-rich Ar or electron-rich Ar^1^ ring afford the corresponding products in relatively high yields (e.g., **2aab**, **2acc**, **2aad** vs. **2aae**, **2aga**, **2aha**, **2aia**). (2) When both Ar and Ar^1^ were electron-rich groups, the desired products were obtained in high isolated yields (e.g., **2adb**, **2aeb**, **2afb**, **2afd**). (3) The steric effect is of importance to affect the reactivity, Ar ring bearing *ortho*-methyl group decreases the reactivity to some extent, giving the products in relatively low yields compared to the substrates with *para*-methyl-substituted Ar rings (**2aba**, **2abb** vs. **2ada**, **2adb**). (4) The reactions of the substrates having either electron-donating group (OMe) or electron-withdrawing group (F) in Ar^2^ ring produced the expected products in 73% (**2baa**) and 77% (**2caa**), respectively. (5) Under the standard reaction conditions, it shows a good tolerance to a variety of valuable and important functional groups in aromatic rings: the aromatic C(*sp*^2^)-Cl, C(*sp*^2^)-F bonds, and CF_3_, CN functional groups in the obtained products (**2aae**, **2aga**, **2aha**, **2aia**, **2afe**, **2aff**, **2agb**, **2ahb**, **2aib**, **2caa**) have highly potential applications in further transformations.

The isolated **2a** could be completely converted into **2aaa** in the presence of Pd(OAc)_2_ under oxygen atmosphere in a solvents mixture of TFA and DMF (1:1 in volume) at 80 °C for 18 h, confirming that **2a** is an intermediate in the formation of **2aaa** (Equation (4)). In addition, a large kinetic isotope effect (KIE, *k_H_*/*k_D_*) of 4.9 was observed in the oxidative C-H/C-H and C-H/C-D dehydrogenative coupling reactions for the formation of **2ada** and **2ada**-*d_5_* from the corresponding intermediates (Equation (5)), indicating that the activation of C-H bond and their coupling reaction may be the rate-limiting step in the stepwise transformation.

## 3. Materials and Methods

### 3.1. General Methods

All commercial reagents are analytically pure and were used directly without further purification. Nuclear magnetic resonance (NMR) spectra were recorded on an ECA-400 spectrometer (JEOL, Tokyo, Japan) using CDCl_3_ as solvent at 298 K. ^1^H NMR (400 MHz) chemical shifts (δ) were referenced to internal standard TMS (for ^1^H, δ = 0.00 ppm). ^13^C NMR (101 MHz) chemical shifts were referenced to internal solvent CDCl_3_ (for ^13^C, δ = 77.16 ppm). The high-resolution mass spectra (HRMS) were obtained on a micrOTOF-Q II spectrometer (Agilent, California, CA, USA) with electron spray ionization (ESI), or on a FT-ICR-MS spectrometer (Solarix Bruker, Bremen, Germany) with Matrix-Assisted Laser Desorption Ionization (MALDI). Single crystals of **2a** were obtained by slow evaporation of their solution in a mixture solvent of CHCl_2_ and petroleum ether (1:3 in volume). *ortho*-(1-Arylethynyl)-*N*-arylbenzamides (substrates) were prepared according to a modified literature method [24], and the characterization data of new substrates are reported below. All the NMR charts for the prepared starting materials, and the products are reported in the Supplementary Materials.

### 3.2. Characterization Data of Substrates

*N*-Phenyl-*ortho*-(*o*-tolylethynyl)benzamide (**1aba**). White solid, mp 115–117 °C. ^1^H NMR (400 MHz, CDCl_3_) δ 9.14 (s, 1H), 8.09−8.06 (m, 1H), 7.63−7.62 (m, 3H), 7.49–7.42 (m, 3H), 7.33–7.20 (m, 4H), 7.17–7.09 (m, 2H), 2.42 (s, 3H); ^13^C NMR (101 MHz, CDCl_3_) δ 164.7, 140.6, 138.0, 136.0, 133.6, 132.1, 130.9, 130.2, 129.9, 129.4, 129.1, 129.0, 125.9, 124.6, 121.7, 120.1, 120.0, 95.6, 91.0, 20.8; HRMS (ESI) m/z: [M + Na]^+^ calcd for C_22_H_17_NONa 334.1202; found 334.1202.

*N*-Phenyl-*ortho*-(*m*-tolylethynyl)benzamide (**1aca**). White solid, mp 106–108 °C. ^1^H NMR (400 MHz, CDCl_3_) δ 9.22 (s, 1H), 8.07–8.05 (m, 1H), 7.67 (d, *J* = 8.0 Hz, 2H), 7.60–7.57 (m, 1H), 7.45–7.38 (m, 2H), 7.33–7.25 (m, 4H), 7.22–7.09 (m, 3H), 2.26 (s, 3H); ^13^C NMR (101 MHz, CDCl_3_) δ 164.6, 138.4, 138.1, 136.1, 133.4, 132.4, 130.9, 130.3, 130.2, 129.1, 129.0, 128.8, 128.5, 124.5, 121.7, 120.1, 119.8, 96.9, 87.0, 21.2; HRMS (ESI) m/z: [M + Na]^+^ calcd for C_22_H_17_NONa 334.1202; found 334.1201.

*N*-Phenyl-*ortho*-((*p*-propylphenyl)ethynyl)benzamide (**1aea**). White solid, mp 115–117 °C. ^1^H NMR (400 MHz, CDCl_3_) δ 9.30 (s, 1H), 8.12–8.09 (m, 1H), 7.66 (d, *J* = 8.0 Hz, 2H), 7.61–7.59 (m, 1H), 7.46–7.41 (m, 2H), 7.38 (d, *J* = 8.0 Hz, 2H), 7.31 (t, *J* = 8.0 Hz, 2H), 7.15–7.09 (m, 3H), 2.58 (t, *J* = 7.6 Hz, 2H), 1.68–1.58 (m, 2H), 0.93 (t, J = 7.2 Hz, 3H); ^13^C NMR (101 MHz, CDCl_3_) δ 164.5, 144.5, 138.1, 135.8, 133.5, 131.7, 130.9, 130.4, 129.1, 128.9, 128.9, 124.5, 120.1, 119.9, 119.1, 97.1, 86.8, 38.1, 24.3, 13.8; HRMS (ESI) m/z: [M + Na]^+^ calcd for C_24_H_21_NONa 362.1515; found 362.1515.

*ortho*-((*p*-Fluorophenyl)ethynyl)-*N*-phenylbenzamide (**1aga**). White solid, mp 153–155 °C. ^1^H NMR (400 MHz, CDCl_3_) δ 9.04 (s, 1H), 8.10–8.07 (m, 1H), 7.66–7.61 (m, 3H), 7.50–7.43 (m, 4H), 7.34 (t, *J* = 7.2 Hz, 2H), 7.14 (t, *J* = 7.2 Hz, 1H), 7.06–7.01 (m, 2H); ^13^C NMR (101 MHz, CDCl_3_) δ 164.6, 163.2 (d, *J*_C-F_ = 252.5 Hz), 138.1, 136.3, 133.8 (d, *J*_C-F_ = 8.1 Hz), 133.5, 131.0, 130.3, 129.3, 124.7, 120.0, 119.6, 118.1 (d, *J*_C-F_ = 4.0 Hz), 116.2 (d, *J*_C-F_ = 22.2 Hz), 95.5, 87.1; HRMS (ESI) m/z: [M + H]^+^ calcd for C_21_H_15_FNO 316.1132; found 316.1132.

*ortho*-((*p*-Chlorophenyl)ethynyl)-*N*-phenylbenzamide (**1aha**). White solid, mp 146–148 °C. ^1^H NMR (400 MHz, CDCl_3_) δ 8.97 (s, 1H), 8.05–8.02 (m, 1H), 7.66–7.59 (m, 3H), 7.49–7.43 (m, 2H), 7.39–7.28 (m, 6H), 7.14 (t, *J* = 7.2 Hz, 1H); ^13^C NMR (101 MHz, CDCl_3_) δ 164.7, 138.0, 136.4, 135.5, 133.5, 133.0, 131.0, 130.2, 129.3, 129.2, 129.1, 124.7, 120.5, 120.0, 119.4, 95.2, 88.2; HRMS (ESI) m/z: [M + H]^+^ calcd for C_21_H_15_ClNO 332.0837; found 332.0836.

*N*-Phenyl-*ortho*-((*p*-(trifluoromethyl)phenyl)ethynyl)benzamide (**1aia**). White solid, mp 143–145 °C. ^1^H NMR (400 MHz, CDCl_3_) δ 8.88 (s, 1H), 7.95–7.91 (m, 1H), 7.64 (d, *J* = 8.0 Hz, 2H), 7.60–7.57 (m, 1H), 7.56–7.48 (m, 4H), 7.46–7.38 (m, 2H), 7.31 (t, *J* = 8.0 Hz, 2H), 7.13 (t, *J* = 7.2 Hz, 1H); ^13^C NMR (101 MHz, CDCl_3_) δ 164.9, 138.0, 137.1, 133.5, 132.0, 130.8, 130.7 (q, *J*_C-F_ = 32.3 Hz), 129.9, 129.5, 129.2, 125.5 (q, *J*_C-F_ = 3.0 Hz), 124.7, 123.8 (q, *J*_C-F_ = 273.7 Hz), 120.0, 119.2, 94.4, 89.4; HRMS (ESI) m/z: [M + Na]^+^ calcd for C_22_H_14_F_3_NONa 388.0920; found 388.0919.

*ortho*-([1,1′-Biphenyl]-4-ylethynyl)-*N*-phenylbenzamide (**1aja**). White solid, mp 141–143 °C. ^1^H NMR (400 MHz, CDCl_3_) δ 9.19 (s, 1H), 8.13–8.10 (m, 1H), 7.69 (d, *J* = 8.0 Hz, 2H), 7.66–7.63 (m, 1H), 7.60–7.51 (m, 6H), 7.49–7.42 (m, 4H), 7.38–7.31 (m, 3H), 7.13 (t, *J* = 7.2 Hz, 1H); ^13^C NMR (101 MHz, CDCl_3_) δ 164.6, 142.1, 140.1, 138.1, 136.1, 133.6, 132.2, 131.0, 130.4, 129.2, 129.2, 129.0, 128.0, 127.4, 127.1, 124.6, 120.8, 120.1, 119.7, 96.6, 88.0; HRMS (ESI) m/z: [M + H]^+^ calcd for C_27_H_20_NO 374.1539; found 374.1539.

*N*-(*p*-Tolyl)-*ortho*-(*o*-tolylethynyl)benzamide (**1abb**). White solid, mp 98–100 °C. ^1^H NMR (400 MHz, CDCl_3_) δ 9.16 (s, 1H), 7.92 (d, *J* = 7.6 Hz, 1H), 7.54–7.49 (m, 3H), 7.39–7.27 (m, 3H), 7.22–7.17 (m, 1H), 7.14 (d, *J* = 7.2 Hz, 1H), 7.10–7.02 (m, 3H), 2.37 (s, 3H), 2.26 (s, 3H); ^13^C NMR (101 MHz, CDCl_3_) δ 164.6, 140.3, 136.2, 135.5, 133.8, 133.2, 131.9, 130.4, 129.6, 129.6, 129.4, 129.1, 128.6, 125.6, 121.8, 120.0, 119.9, 95.0, 91.0, 20.8, 20.6; HRMS (ESI) m/z: [M + Na]^+^ calcd for C_23_H_19_NONa 348.1359; found 348.1358.

*N*-(*p*-Tolyl)-*ortho*-(*m*-tolylethynyl)benzamide (**1acb**). White solid, mp 119–121 °C. ^1^H NMR (400 MHz, CDCl_3_) δ 9.18 (s, 1H), 8.06–8.04 (m, 1H), 7.58–7.53 (m, 3H), 7.43–7.36 (m, 2H), 7.28–7.25 (m, 2H), 7.22–7.13 (m, 2H), 7.10 (d, *J* = 8.0 Hz, 2H), 2.30 (s, 3H), 2.26 (s, 3H); ^13^C NMR (101 MHz, CDCl_3_) δ 164.4, 138.3, 136.1, 135.5, 134.0, 133.4, 132.4, 130.7, 130.2, 130.1, 129.5, 128.9, 128.7, 128.5, 121.7, 120.1, 119.7, 96.7, 87.1, 21.2, 20.9; HRMS (ESI) m/z: [M + H]^+^ calcd for C_23_H_20_NO 326.1539; found 326.1539.

*N*-(*p*-Tolyl)-*ortho*-(*p*-tolylethynyl)benzamide (**1adb**). White solid, mp 125–127 °C. ^1^H NMR (400 MHz, CDCl_3_) δ 9.24 (s, 1H), 8.13–8.09 (m, 1H), 7.61–7.58 (m, 1H), 7.54 (d, *J* = 8.0 Hz, 2H), 7.44–7.41 (m, 2H), 7.37 (d, *J* = 7.6 Hz, 2H), 7.13 (t, *J* = 8.0 Hz, 4H), 2.36 (s, 3H), 2.31 (s, 3H); ^13^C NMR (101 MHz, CDCl_3_) δ 164.3, 139.7, 135.8, 135.6, 134.1, 133.5, 131.7, 130.8, 130.4, 129.6, 129.5, 128.9, 120.1, 119.8, 118.9, 96.9, 86.9, 21.7, 21.0; HRMS (ESI) m/z: [M + Na]^+^ calcd for C_23_H_19_NONa 348.1359; found 348.1358.

*ortho*-((*p*-Propylphenyl)ethynyl)-*N*-(*p*-tolyl)benzamide (**1aeb**). White solid, mp 116–118 °C. ^1^H NMR (400 MHz, CDCl_3_) δ 9.29 (s, 1H), 7.95 (d, *J* = 7.6 Hz, 1H), 7.54 (d, *J* = 8.0 Hz, 2H), 7.47 (d, *J* = 7.2 Hz, 1H), 7.34–7.23 (m, 4H), 7.04 (t, *J* = 8.0 Hz, 4H), 2.51 (t, *J* = 7.6 Hz, 2H), 2.26 (s, 3H), 1.62–1.52 (m, 2H), 0.89 (t, *J* = 7.6 Hz, 3H); ^13^C NMR (101 MHz, CDCl_3_) δ 164.3, 143.9, 135.8, 135.5, 133.6, 133.1, 131.4, 130.4, 129.8, 129.3, 128.5, 128.4, 119.9, 119.7, 119.0, 96.5, 86.7, 37.7, 24.1, 20.7, 13.6; HRMS (ESI) m/z: [M + H]^+^ calcd for C_25_H_24_NO 354.1852; found 354.1852.

*ortho*-((*p*-Methoxyphenyl)ethynyl)-*N*-(*p*-tolyl)benzamide (**1afb**). White solid, mp 105–107 °C. ^1^H NMR (400 MHz, CDCl_3_) δ 9.28 (s, 1H), 8.11–8.08 (m, 1H), 7.59–7.53 (m, 3H), 7.44–7.36 (m, 4H), 7.11 (d, *J* = 7.6 Hz, 2H), 6.86–6.83 (m, 2H), 3.79 (s, 3H), 2.31 (s, 3H); ^13^C NMR (101 MHz, CDCl_3_) δ 164.3, 160.4, 135.7, 135.6, 134.0, 133.4, 133.3, 130.8, 130.3, 129.6, 128.7, 120.1, 119.9, 114.3, 113.9, 96.9, 86.3, 55.4, 21.0; HRMS (ESI) m/z: [M + H]^+^ calcd for C_23_H_20_NO_2_ 342.1489; found 342.1488.

*ortho*-((*p*-Fluorophenyl)ethynyl)-*N*-(*p*-tolyl)benzamide (**1agb**). White solid, mp 142–144 °C. ^1^H NMR (400 MHz, CDCl_3_) δ 9.02 (s, 1H), 8.05–8.02 (m, 1H), 7.60–7.57 (m, 1H), 7.52 (d, *J* = 8.0 Hz, 2H), 7.46–7.41 (m, 4H), 7.12 (d, *J* = 8.0 Hz, 2H), 7.01 (t, *J* = 8.4 Hz, 2H), 2.32 (s, 3H); ^13^C NMR (101 MHz, CDCl_3_) δ 164.5, 163.1 (d, *J*_C-F_ = 251.5 Hz), 136.4, 135.5, 134.2, 133.8 (d, *J*_C-F_ = 9.1 Hz), 133.4, 130.8, 130.2, 129.7, 129.2, 120.0, 119.5, 118.2 (d, *J*_C-F_ = 3.0 Hz), 116.1 (d, *J*_C-F_ = 22.2 Hz), 95.3, 87.2, 21.0; HRMS (ESI) m/z: [M + H]^+^ calcd for C_22_H_17_FNO 330.1289; found 330.1288.

*ortho*-((*p*-Chlorophenyl)ethynyl)-*N*-(*p*-tolyl)benzamide (**1ahb**). White solid, mp 155–157 °C. ^1^H NMR (400 MHz, CDCl_3_) δ 8.94 (s, 1H), 8.01–7.98 (m, 1H), 7.58–7.56 (m, 1H), 7.51 (d, *J* = 8.4 Hz, 2H), 7.43–7.40 (m, 2H), 7.36 (d, *J* = 8.0 Hz, 2H), 7.28 (d, *J* = 8.0 Hz, 2H), 7.11 (d, *J* = 8.0 Hz, 2H), 3.32 (s, 3H); ^13^C NMR (101 MHz, CDCl_3_) δ 164.5, 136.5, 135.5, 134.3, 133.5, 133.0, 130.9, 130.3, 129.8, 129.4, 129.1, 127.8, 120.6, 120.0, 119.4, 95.2, 88.3, 21.1; HRMS (ESI) m/z: [M + H]^+^ calcd for C_22_H_17_ClNO 346.0993; found 346.0993.

*N*-(*p*-Tolyl)-*ortho*-((*p*-(trifluoromethyl)phenyl)ethynyl)benzamide (**1aib**). White solid, mp 154–159 °C. ^1^H NMR (400 MHz, CDCl_3_) δ 8.81 (s, 1H), 7.97–7.94 (m, 1H), 7.61–7.51 (m, 7H), 7.45–7.40 (m, 2H), 7.12 (d, *J* = 8.0 Hz, 2H), 2.32 (s, 3H); ^13^C NMR (101 MHz, CDCl_3_) δ 164.7, 137.1, 135.4, 134.4, 133.5, 132.0, 130.8 (q, *J*_C-F_ = 33.3 Hz), 130.7, 129.9, 129.7, 129.6, 126.0, 125.6 (q, *J*_C-F_ = 3.0 Hz), 123.9 (q, *J*_C-F_ = 273.7 Hz), 120.0, 119.1, 94.4, 89.5, 21.0; HRMS (ESI) m/z: [M + H]^+^ calcd for C_23_H_17_F_3_NO 380.1257; found 380.1256.

*ortho*-([1,1′-Biphenyl]-4-ylethynyl)-*N*-(p-tolyl)benzamide (**1ajb**). White solid, mp 216–218 °C. ^1^H NMR (400 MHz, CDCl_3_) δ 9.16 (s, 1H), 8.16–8.13 (m, 1H), 7.68–7.65 (m, 1H), 7.62–7.54 (m, 8H), 7.52–7.44 (m, 4H), 7.40–7.36 (m, 1H), 7.15 (d, *J* = 8.4 Hz, 2H), 2.33 (s, 3H); ^13^C NMR (101 MHz, CDCl_3_) δ 164.4, 142.1, 140.1, 136.1, 135.6, 134.3, 133.6, 132.3, 131.0, 130.5, 129.8, 129.2, 129.1, 128.1, 127.4, 127.2, 120.8, 120.1, 119.7, 96.6, 88.1, 21.1; HRMS (ESI) m/z: [M + H]^+^ calcd for C_28_H_22_NO 388.1696; found 388.1695.

*N*-(*p*-Isopropylphenyl)-*ortho*-(phenylethynyl)benzamide (**1aac**). White solid, mp 105–107 °C. ^1^H NMR (400 MHz, CDCl_3_) δ 9.26 (s, 1H), 7.92 (d, *J* = 7.6 Hz, 1H), 7.57 (d, *J* = 8.0 Hz, 2H), 7.50 (d, *J* = 7.6 Hz, 1H), 7.42–7.39 (m, 2H), 7.33–7.22 (m, 5H), 7.10 (d, *J* = 8.0 Hz, 2H), 2.89–2.78 (m, 1H), 1.21 (d, *J* = 6.8 Hz, 6H); ^13^C NMR (101 MHz, CDCl_3_) δ 164.5, 144.8, 136.2, 135.7, 133.2, 131.6, 130.4, 129.7, 129.0, 128.7, 128.4, 126.7, 121.9, 120.1, 119.6, 96.0, 87.3, 33.5, 23.9; HRMS (ESI) m/z: [M + Na]^+^ calcd for C_24_H_21_NONa 362.1515; found 362.1515.

*N*-(*p*-Cyanophenyl)-*ortho*-(phenylethynyl)benzamide (**1aae**). Pale yellow solid, mp 118–120 °C. ^1^H NMR (400 MHz, CDCl_3_) δ 9.50 (s, 1H), 8.13–8.10 (m, 1H), 7.79–7.75 (m, 2H), 7.68–7.65 (m, 1H), 7.61–7.57 (m, 2H), 7.53–7.46 (m, 4H), 7.43–7.35 (m, 3H); ^13^C NMR (101 MHz, CDCl_3_) δ 164.8, 142.0, 135.0, 133.8, 133.5, 131.7, 131.6, 130.6, 129.8, 129.4, 128.9, 121.6, 119.9, 119.7, 118.9, 107.4, 97.2, 87.1; HRMS (ESI) m/z: [M + H]^+^ calcd for C_22_H_15_N_2_O 323.1179; found 323.1178.

*N*-(*p*-Cyanophenyl)-*ortho*-((*p*-methoxyphenyl)ethynyl)benzamide (**1afe**). Pale yellow solid, mp 165–167 °C. ^1^H NMR (400 MHz, CDCl_3_) δ 9.64 (s, 1H), 8.08–8.05 (m, 1H), 7.76 (d, *J* = 8.8 Hz, 2H), 7.62–7.55 (m, 3H), 7.50–7.37 (m, 4H), 6.90–6.86 (m, 2H), 3.84 (s, 3H); ^13^C NMR (101 MHz, CDCl_3_) δ 164.8, 160.7, 142.1, 134.6, 133.5, 133.4, 133.3, 131.5, 130.4, 128.9, 120.1, 119.8, 118.9, 114.5, 113.4, 107.2, 97.5, 86.0, 55.5; HRMS (ESI) m/z: [M + H]^+^ calcd for C_23_H_17_N_2_O_2_ 353.1285; found 353.1284.

*ortho*-((*p*-Methoxyphenyl)ethynyl)-*N*-(*p*-(trifluoromethyl)phenyl)benzamide (**1aff**). White solid, mp 130–132 °C. ^1^H NMR (400 MHz, CDCl_3_) δ 9.56 (s, 1H), 7.98 (d, *J* = 7.6 Hz, 1H), 7.74 (d, *J* = 8.4 Hz, 2H), 7.51 (t, *J* = 8.4 Hz, 3H), 7.40–7.30 (m, 4H), 6.84–6.81 (m, 2H), 3.78 (s, 3H); ^13^C NMR (101 MHz, CDCl_3_) δ 164.9, 160.6, 141.3, 135.1, 133.4, 133.2, 133.1, 130.2, 128.7, 126.3 (q, *J*_C-F_ = 4.0 Hz), 126.0 (q, *J*_C-F_ = 32.3 Hz), 124.2 (q, *J*_C-F_ = 272.7 Hz), 120.2, 119.7, 114.4, 113.7, 97.2, 86.1, 55.3; HRMS (ESI) m/z: [M + H]^+^ calcd for C_23_H_17_F_3_NO_2_ 396.1206; found 396.1206.

*p*-Methoxy-*N*-phenyl-*ortho*-(phenylethynyl)benzamide (**1baa**). White solid, mp 163–165 °C. ^1^H NMR (400 MHz, CDCl_3_) δ 9.33 (s, 1H), 8.11 (d, *J* = 8.8 Hz, 1H), 7.65 (d, *J* = 7.6 Hz, 2H), 7.52–7.49 (m, 2H), 7.39–7.28 (m, 5H), 7.13–7.08 (m, 2H), 6.99–6.96 (m, 1H), 3.85 (s, 3H); ^13^C NMR (101 MHz, CDCl_3_) δ 164.1, 161.3, 138.3, 132.6, 131.8, 129.5, 129.1, 128.8, 128.2, 124.3, 121.7, 121.0, 120.0, 118.0, 115.5, 96.5, 87.5, 55.7; HRMS (ESI) m/z: [M + H]^+^ calcd for C22H18NO2 328.1332; found 328.1331.

*p*-Fluoro-*N*-phenyl-*ortho*-(phenylethynyl)benzamide (**1caa**). White solid, mp 164–166 °C. ^1^H NMR (400 MHz, CDCl_3_) δ 9.19 (s, 1H), 8.09–8.06 (m, 1H), 7.63 (d, *J* = 7.6 Hz, 2H), 7.47–7.44 (m, 2H), 7.40–7.23 (m, 6H), 7.14–7.06 (m, 2H); ^13^C NMR (101 MHz, CDCl_3_) δ 163.7, 163.6 (d, *J*_C-F_ = 252.5 Hz), 138.0, 132.9 (d, *J*_C-F_ = 10.1 Hz), 132.4 (d, *J*_C-F_ = 3.0 Hz), 131.8, 129.7, 129.2, 128.8, 124.6, 121.9 (d, *J*_C-F_ = 10.1 Hz), 120.1, 120.0, 119.8, 116.6 (d, *J*_C-F_ = 21.2 Hz), 97.5, 86.2; HRMS (ESI) m/z: [M + H]^+^ calcd for C_21_H_15_FNO 316.1132; found 316.1132.

### 3.3. Typical Experimental Procedure for the Synthesis of 11-Phenyl-6H-Isoindolo[2,1-a]Indol-6-One (**2aaa**)

A mixture of *N*-phenyl-*ortho*-(phenylethynyl)benzamide (**1aaa**, 148.7 mg, 0.5 mmol), CuI (4.8 mg, 0.025 mmol), and L-proline (8.6 mg, 0.075 mmol) in DMF (1.0 mL) in a 25 mL screw-capped thick-walled Pyrex tube was stirred at 80 °C for 6 h under nitrogen atmosphere. After the reaction mixture was cooled to room temperature, Pd(OAc)_2_ (5.6 mg, 0.025 mmol) and TFA (1.0 mL) was added directly, and the obtained mixture was further heated at 80 °C for 18 h under oxygen atmosphere (1 atm). It was then poured into a solvent mixture of water (15.0 mL) and ethyl acetate (20.0 mL), and the two phases were then separated. The aqueous layer was extracted with ethyl acetate (3 × 15.0 mL). After the combined organic extracts were dried over MgSO_4_ overnight, the filtered solution was concentrated under reduced pressure. The crude product was purified by column chromatography on silica gel with the use of petroleum ether/ethyl acetate as eluent to afford **2aaa** as a pale yellow solid (122.1 mg, 83%).

### 3.4. Characterization Data of Products

11-Phenyl-6*H*-isoindolo[2,1-*a*]indol-6-one (**2aaa**) [11]. Pale yellow solid (122 mg, 83% yield), mp 223–225 °C. ^1^H NMR (400 MHz, CDCl_3_) δ 7.95 (d, *J* = 8.0 Hz, 1H), 7.76 (d, *J* = 7.6 Hz, 1H), 7.71–7.68 (m, 2H), 7.58–7.52 (m, 4H), 7.48–7.43 (m, 1H), 7.42–7.37 (m, 1H), 7.35–7.28 (m, 2H), 7.20–7.15 (m, 1H); ^13^C NMR (101 MHz, CDCl_3_) δ 162.7, 134.8, 134.3, 134.1, 134.0, 133.9, 133.7, 132.3, 129.2, 129.1, 128.9, 128.4, 126.9, 125.5, 124.2, 121.4, 121.3, 120.7, 113.6; HRMS (MALDI) m/z: [M]^+^ calcd for C_21_H_13_NO 295.0092; found 295.0091.

11-(*o*-Tolyl)-6*H*-isoindolo[2,1-*a*]indol-6-one (**2aba**). Pale yellow solid (110.3 mg, 71% yield), mp 172–174 °C. ^1^H NMR (400 MHz, CDCl_3_) δ 7.93 (d, *J* = 8.0 Hz, 1H), 7.74 (d, *J* = 7.6 Hz, 1H), 7.43–7.36 (m, 3H), 7.35−7.17 (m, 5H), 7.13−7.05 (m, 2H), 2.29 (s, 3H); ^13^C NMR (101 MHz, CDCl_3_) δ 162.6, 137.2, 135.0, 134.9, 134.7, 134.0, 133.6, 133.5, 131.2, 130.8, 130.3, 128.7, 128.6, 126.6, 126.1, 125.3, 124.0, 121.6, 121.5, 119.6, 113.5, 20.2; HRMS (MALDI) m/z: [M]^+^ calcd for C_22_H_15_NO 309.1149; found 309.1147.

11-(*m*-Tolyl)-6*H*-isoindolo[2,1-*a*]indol-6-one (**2aca**) [11]. Pale yellow solid (124.0 mg, 80% yield), mp 219–221 °C. ^1^H NMR (400 MHz, CDCl_3_) δ 7.95 (d, *J* = 8.0 Hz, 1H), 7.76 (d, *J* = 7.6 Hz, 1H), 7.58–7.48 (m, 4H), 7.46–7.37 (m, 2H), 7.34–7.24 (m, 3H), 7.19–7.15 (m, 1H), 2.47 (s, 3H); ^13^C NMR (101 MHz, CDCl_3_) δ 162.7, 138.9, 134.9, 134.2, 134.1, 134.0, 133.9, 133.7, 132.2, 129.7, 129.2, 129.1, 128.8, 126.9, 126.2, 125.5, 124.1, 121.5, 121.3, 120.9, 113.6, 21.7; HRMS (MALDI) m/z: [M]^+^ calcd for C_22_H_15_NO 309.1149; found 309.1152.

11-(*p*-Tolyl)-6*H*-isoindolo[2,1-*a*]indol-6-one (**2ada**) [11]. Pale yellow solid (133.9 mg, 87% yield), mp 174–176 °C. ^1^H NMR (400 MHz, CDCl_3_) δ 7.92 (d, *J* = 8.0 Hz, 1H), 7.72 (d, *J* = 7.2 Hz, 1H), 7.57–7.53 (m, 3H), 7.51–7.49 (m, 1H), 7.38–7.30 (m, 3H), 7.29–7.23 (m, 2H), 7.16–7.11 (m, 1H), 2.45 (s, 3H); ^13^C NMR (101 MHz, CDCl_3_) δ 162.6, 138.3, 134.8, 134.1, 134.0, 133.9, 133.8, 133.6, 129.8, 129.3, 128.9, 128.7, 126.8, 125.4, 124.1, 121.4, 121.3, 120.7, 113.6, 21.5; HRMS (MALDI) m/z: [M]^+^ calcd for C_22_H_15_NO 309.1149; found 309.1147.

11-(*p*-Propylphenyl)-6*H*-isoindolo[2,1-*a*]indol-6-one (**2aea**). Pale yellow solid (146.7 mg, 87% yield), mp 181–183 °C. ^1^H NMR (400 MHz, CDCl_3_) δ 7.93 (d, *J* = 8.0 Hz, 1H), 7.74 (d, *J* = 7.6 Hz, 1H), 7.60–7.56 (m, 3H), 7.53 (d, *J* = 8.0 Hz, 1H), 7.39–7.23 (m, 5H), 7.17–7.12 (m, 1H), 2.68 (t, *J* = 8.0 Hz, 2H), 1.78–1.68 (m, 2H), 1.02 (t, *J* = 7.2 Hz, 3H); ^13^C NMR (101 MHz, CDCl_3_) δ 162.6, 143.1, 134.8, 134.2, 134.0, 133.9, 133.8, 133.6, 129.5, 129.2, 128.9, 128.7, 126.8, 125.4, 124.1, 121.5, 121.3, 120.8, 113.6, 38.1, 24.6, 14.1; HRMS (MALDI) m/z: [M]^+^ calcd for C_24_H_19_NO 337.1462; found 337.1459.

11-(*p*-Methoxyphenyl)-6*H*-isoindolo[2,1-*a*]indol-6-one (**2afa**). Pale yellow solid (140.4 mg, 86% yield), mp 195−197 °C. ^1^H NMR (400 MHz, CDCl_3_) δ 7.95 (d, *J* = 8.0 Hz, 1H), 7.77 (d, *J* = 7.6 Hz, 1H), 7.63 (d, *J* = 7.2 Hz, 2H), 7.57 (d, *J* = 7.6 Hz, 1H), 7.52 (d, *J* = 7.6 Hz, 1H), 7.42−7.37 (m, 1H), 7.34−7.27 (m, 2H), 7.20−7.15 (m, 1H), 7.08 (d, *J* = 7.2 Hz, 2H), 3.91 (s, 3H); ^13^C NMR (101 MHz, CDCl_3_) δ 162.6, 159.8, 134.9, 134.3, 134.0, 133.9, 133.8, 133.7, 130.3, 128.7, 126.9, 125.5, 124.5, 124.1, 121.4, 121.2, 120.5, 114.6, 113.6, 55.5; HRMS (MALDI) m/z: [M]^+^ calcd for C_22_H_15_NO_2_ 325.1098; found 325.1098.

11-(*p*-Fluorophenyl)-6*H*-isoindolo[2,1-*a*]indol-6-one (**2aga**). Pale yellow solid (110.2 mg, 70% yield), mp 230–232 °C. ^1^H NMR (400 MHz, CDCl_3_) δ 7.96 (d, *J* = 8.0 Hz, 1H), 7.78 (d, *J* = 7.6 Hz, 1H), 7.69–7.64 (m, 2H), 7.52–7.46 (m, 2H), 7.41 (t, *J* = 7.6 Hz, 1H), 7.35–7.29 (m, 2H), 7.27–7.22 (m, 2H), 7.18 (t, *J* = 7.6 Hz, 1H); ^13^C NMR (101 MHz, CDCl_3_) δ 162.8 (d, *J*_C-F_ = 249.5 Hz), 162.6, 134.6, 134.3, 134.0 (d, *J*_C-F_ = 3.0 Hz), 133.8, 133.7, 130.9, 130.8, 129.0, 128.3, 128.2, 127.0, 125.5, 124.2, 121.1 (d, *J*_C-F_ = 5.1 Hz), 119.5, 116.3 (d, *J*_C-F_ = 21.2 Hz), 113.7; ^19^F NMR (376 MHz, CDCl_3_) δ -112.6; HRMS (MALDI) m/z: [M]^+^ calcd for C_21_H_12_FNO 313.0898; found 313.0896.

11-(*p*-Chlorophenyl)-6*H*-isoindolo[2,1*-a*]indol-6-one (**2aha**). Pale yellow solid (118.8 mg, 72% yield), mp 208–210 °C. ^1^H NMR (400 MHz, CDCl_3_) δ 7.93 (d, *J* = 8.0 Hz, 1H), 7.76 (d, *J* = 7.2 Hz, 1H), 7.63–7.60 (m, 2H), 7.53–7.50 (m, 3H), 7.47 (d, *J* = 8.0 Hz, 1H), 7.43–7.38 (m, 1H), 7.34–7.29 (m, 2H), 7.20–7.15 (m, 1H); ^13^C NMR (101 MHz, CDCl_3_) δ 162.6, 134.5, 134.4, 134.3, 133.9, 133.8, 133.8, 133.7, 130.8, 130.4, 129.5, 129.1, 127.1, 125.6, 124.3, 121.2, 121.0, 119.3, 113.7; HRMS (MALDI) m/z: [M]^+^ calcd for C_21_H_12_ClNO 329.0602; found 329.0600.

11-(*p*-(Trifluoromethyl)phenyl)-6*H*-isoindolo[2,1-*a*]indol-6-one (**2aia**). Pale yellow solid (120.1 mg, 66% yield), mp 196–198 °C. ^1^H NMR (400 MHz, CDCl_3_) δ 7.90 (d, *J* = 8.0 Hz, 1H), 7.83–7.74 (m, 4H), 7.72 (d, *J* = 7.2 Hz, 1H), 7.49–7.44 (m, 2H), 7.41–7.36 (m, 1H), 7.32–7.27 (m, 2H), 7.18–7.13 (m, 1H); ^13^C NMR (101 MHz, CDCl_3_) δ 162.5, 136.2, 135.0, 134.3, 133.9, 133.8, 133.7, 133.4, 130.3 (q, *J*_C-F_ = 32.3 Hz), 129.4, 129.3, 127.1, 126.1 (q, *J*_C-F_ = 4.0 Hz), 125.6, 124.4, 124.2 (q, *J*_C-F_ = 272.7 Hz), 121.2, 120.9, 118.8, 113.7; ^19^F NMR (376 MHz, CDCl_3_) δ -62.4; HRMS (MALDI) m/z: [M]^+^ calcd for C_22_H_12_F_3_NO 363.0866; found 363.0869.

11-([1,1′-Biphenyl]-4-yl)-6*H*-isoindolo[2,1-*a*]indol-6-one (**2aja**). Pale yellow solid (144.3 mg, 78% yield), mp 234−236 °C. ^1^H NMR (400 MHz, CDCl_3_) δ 7.98 (d, *J* = 8.0 Hz, 1H), 7.81−7.78 (m, 5H), 7.71–7.68 (m, 2H), 7.65 (d, *J* = 7.6 Hz, 1H), 7.59 (d, *J* = 7.6 Hz, 1H), 7.49 (t, *J* = 7.6 Hz, 2H), 7.45–7.30 (m, 4H), 7.23–7.18 (m, 1H); ^13^C NMR (101 MHz, CDCl_3_) δ 162.7, 141.4, 140.7, 134.9, 134.5, 134.2, 134.1, 134.0, 133.8, 131.4, 129.6, 129.1, 129.0, 127.9, 127.8, 127.2, 127.0, 125.6, 124.3, 121.4, 120.4, 113.7; HRMS (MALDI) m/z: [M]^+^ calcd for C_27_H_17_NO 371.1305; found 371.1306.

2-Methyl-11-phenyl-6*H*-isoindolo[2,1-*a*]indol-6-one (**2aab**) [11]. Pale yellow solid (136.1 mg, 88% yield), mp 178–180 °C. ^1^H NMR (400 MHz, CDCl_3_) δ 7.81 (d, *J* = 7.6 Hz, 1H), 7.75–7.72 (m, 1H), 7.69–7.66 (m, 2H), 7.57–7.52 (m, 3H), 7.48–7.43 (m, 1H), 7.39–7.34 (m, 1H), 7.31–7.24 (m, 2H), 7.14–7.10 (m, 1H), 2.37 (s, 3H); ^13^C NMR (101 MHz, CDCl_3_) δ 162.5, 134.8, 134.5, 134.3, 134.0, 133.8, 133.6, 132.4, 132.0, 129.1, 128.7, 128.4, 128.0, 125.4, 121.4, 121.2, 120.5, 113.2, 21.7; HRMS (ESI) m/z: [M + H]^+^ calcd for C_22_H_16_NO 310.1226; found 310.1228.

2-Methyl-11-(*o*-tolyl)-6*H*-isoindolo[2,1-*a*]indol-6-one (**2abb**). Pale yellow solid (124.8 mg, 77% yield), mp 161–163 °C. ^1^H NMR (400 MHz, CDCl_3_) δ 7.78 (d, *J* = 7.6 Hz, 1H), 7.71 (d, *J* = 8.0 Hz, 1H), 7.52–7.39 (m, 4H), 7.36–7.32 (m, 1H), 7.28–7.21 (m, 3H), 7.09 (d, *J* = 8.0 Hz, 1H), 2.46 (s, 3H), 2.35 (s, 3H); ^13^C NMR (101 MHz, CDCl_3_) δ 162.4, 138.8, 134.8, 134.3, 134.2, 134.0, 133.7, 133.5, 132.3, 131.9, 129.6, 129.1, 129.0, 128.6, 127.9, 126.2, 125.3, 121.4, 121.1, 120.6, 113.1, 21.7, 21.6; HRMS (MALDI) m/z: [M]^+^ calcd for C_23_H_17_NO 323.1305; found 323.1310.

2-Methyl-11-(*m*-tolyl)-6H-isoindolo[2,1-*a*]indol-6-one (**2acb**). Pale yellow solid (137.9 mg, 85% yield), mp 162–165 °C. ^1^H NMR (400 MHz, CDCl_3_) δ 7.82 (d, *J* = 8.0 Hz, 1H), 7.75 (d, *J* = 7.2 Hz, 1H), 7.54 (d, *J* = 7.2 Hz, 1H), 7.50–7.41 (m, 3H), 7.40–7.35 (m, 1H), 7.31–7.26 (m, 3H), 7.13 (d, J = 7.6 Hz, 1H), 2.48 (s, 3H), 2.38 (s, 3H); ^13^C NMR (101 MHz, CDCl_3_) δ 162.6, 138.8, 134.9, 134.4, 134.4, 134.1, 133.8, 133.6, 132.4, 132.0, 129.7, 129.2, 129.0, 128.7, 128.0, 126.2, 125.4, 121.5, 121.2, 120.7, 113.2, 21.7, 21.6; HRMS (MALDI) m/z: [M]^+^ calcd for C_23_H_17_NO 323.1305; found 323.1302.

2-Methyl-11-(*p*-tolyl)-6*H*-isoindolo[2,1-*a*]indol-6-one (**2adb**). Pale yellow solid (145.7 mg, 90% yield), mp 206−208 °C. ^1^H NMR (400 MHz, CDCl_3_) δ 7.79 (d, *J* = 7.6 Hz, 1H), 7.72 (d, *J* = 7.6 Hz, 1H), 7.57−7.52 (m, 3H), 7.37−7.33 (m, 3H), 7.29−7.23 (m, 2H), 7.10 (d, *J* = 8.0 Hz, 1H), 2.46 (s, 3H), 2.36 (s, 3H); ^13^C NMR (101 MHz, CDCl_3_) δ 162.5, 138.3, 134.8, 134.4, 134.2, 134.0, 133.7, 133.5, 132.0, 129.8, 129.4, 129.0, 128.6, 127.9, 125.3, 121.4, 121.2, 120.6, 113.2, 21.6, 21.5; HRMS (ESI) m/z: [M + H]^+^ calcd for C_23_H_18_NO 324.1383; found 324.1384.

2-Methyl-11-(*p*-propylphenyl)-6*H*-isoindolo[2,1-*a*]indol-6-one (**2aeb**). Pale yellow solid (159.8 mg, 91% yield), mp 133–135 °C. ^1^H NMR (400 MHz, CDCl_3_) δ 7.75–7.71 (m, 1H), 7.66 (d, *J* = 7.2 Hz, 1H), 7.54–7.52 (m, 2H), 7.49 (d, *J* = 7.6 Hz, 1H), 7.31–7.25 (m, 4H), 7.21–7.15 (m, 1H), 7.04 (d, *J* = 8.0 Hz, 1H), 2.66 (t, *J* = 7.6 Hz, 2H), 2.32 (s, 3H), 1.77–1.66 (m, 2H), 1.01 (d, *J* = 7.6 Hz, 3H); ^13^C NMR (101 MHz, CDCl_3_) δ 162.3, 142.9, 134.7, 134.3, 134.1, 133.9, 133.6, 133.4, 131.9, 129.6, 129.1, 128.9, 128.4, 127.8, 125.1, 121.4, 121.1, 120.5, 113.1, 38.0, 24.6, 21.6, 14.1; HRMS (MALDI) m/z: [M]^+^ calcd for C_25_H_21_NO 351.1618; found 351.1616.

11-(*p*-Methoxyphenyl)-2-methyl-6*H*-isoindolo[2,1-*a*]indol-6-one (**2afb**). Pale yellow solid (148.9 mg, 88% yield), mp 158–160 °C. ^1^H NMR (400 MHz, CDCl_3_) δ 7.78 (d, *J* = 8.0 Hz, 1H), 7.71 (d, *J* = 7.6 Hz, 1H), 7.60–7.57 (m, 2H), 7.51 (d, *J* = 7.6 Hz, 1H), 7.37–7.32 (m, 1H), 7.27–7.22 (m, 2H), 7.11–7.05 (m, 3H), 3.89 (s, 3H), 2.36 (s, 3H); ^13^C NMR (101 MHz, CDCl_3_) δ 162.4, 159.7, 134.8, 134.4, 134.0, 133.9, 133.7, 133.5, 132.0, 130.3, 128.5, 127. 9, 125.3, 124.6, 121.4, 121.0, 120.3, 114.5, 113.2, 55.5, 21.6; HRMS (ESI) m/z: [M + H]^+^ calcd for C_23_H_18_NO_2_ 340.1332; found 340.1333.

11-(*p*-Fluorophenyl)-2-methyl-6*H*-isoindolo[2,1-*a*]indol-6-one (**2agb**). Pale yellow solid (124.3 mg, 76% yield), mp 157–159 °C. ^1^H NMR (400 MHz, CDCl_3_) δ 7.80 (d, *J* = 8.0 Hz, 1H), 7.76–7.37 (m, 1H), 7.68–7.62 (m, 2H), 7.49–7.46 (m, 1H), 7.41–7.36 (m, 1H), 7.32–7.21 (m, 4H), 7.15–7.11 (m, 1H), 2.38 (s, 3H); ^13^C NMR (101 MHz, CDCl_3_) δ 162.8 (d, *J*_C-F_ = 248.5 Hz), 162.5, 161.5, 134.6, 134.2, 134.0, 133.9, 133.7, 131.9, 130.9, 130.8, 128.9, 128.4 (d, *J*_C-F_ = 3.0 Hz), 128.1, 125.5, 121.1 (d, *J*_C-F_ = 13.1 Hz), 119.3, 116.2 (d, *J*_C-F_ = 22.2 Hz), 113.3, 21.7; ^19^F NMR (376 MHz, CDCl_3_) δ -112.7; HRMS (MALDI) m/z: [M]^+^ calcd for C_22_H_14_FNO 327.1054; found 327.1053.

11-(*p*-Chlorophenyl)-2-methyl-6*H*-isoindolo[2,1-*a*]indol-6-one (**2ahb**). Pale yellow solid (131.7 mg, 77% yield), mp 222−224 °C. ^1^H NMR (400 MHz, CDCl_3_) δ 7.79 (d, *J* = 7.6 Hz, 1H), 7.72 (d, *J* = 7.6 Hz, 1H), 7.57−7.52 (m, 3H), 7.37−7.33 (m, 3H), 7.29−7.23 (m, 2H), 7.10 (d, *J* = 8.0 Hz, 1H), 2.46 (s, 3H), 2.36 (s, 3H); ^13^C NMR (101 MHz, CDCl_3_) δ 162.5, 138.3, 134.8, 134.4, 134.2, 134.0, 133.7, 133.5, 132.0, 129.8, 129.4, 129.0, 128.6, 127.9, 125.3, 121.4, 121.2, 120.6, 113.2, 21.6, 21.5; HRMS (ESI) m/z: [M + H]^+^ calcd for C_22_H_15_ClNO 344.0837; found 344.0836.

2-Methyl-11-(*p-*(trifluoromethyl)phenyl)-6*H*-isoindolo[2,1-*a*]indol-6-one (**2aib**). Pale yellow solid (132.2 mg, 70% yield), mp 180–182 °C. ^1^H NMR (400 MHz, CDCl_3_) δ 7.81–7.75 (m, 5H), 7.71 (d, *J* = 7.6 Hz, 1H), 7.46 (d, *J* = 7.6 Hz, 1H), 7.40–7.35 (m, 1H), 7.30–7.26 (m, 1H), 7.23 (s, 1H), 7.12–7.09 (m, 1H), 2.36 (s, 3H); ^13^C NMR (101 MHz, CDCl_3_) δ 162.4, 136.4, 135.1, 134.3, 134.1, 133.9, 133.8, 133.7, 131.8, 130.2 (q, *J*_C-F_ = 33.3 Hz), 129.4, 129.1, 128.3, 126.1 (q, *J*_C-F_ = 4.0 Hz), 125.5, 124.2 (q, *J*_C-F_ = 272.7 Hz), 121.0, 120.9, 118.7, 113.3, 21.6; ^19^F NMR (376 MHz, CDCl_3_) δ -62.4; HRMS (MALDI) m/z: [M]^+^ calcd for C_23_H_14_F_3_NO 377.1022; found 377.1020.

11-([1,1′-Biphenyl]-4-yl)-2-methyl-6*H*-isoindolo[2,1-*a*]indol-6-one (**2ajb**). Pale yellow solid (153.8 mg, 80% yield), mp 235–237 °C. ^1^H NMR (400 MHz, CDCl_3_) δ 7.85 (d, *J* = 7.6 Hz, 1H), 7.81–7.75 (m, 5H), 7.71–7.68 (m, 2H), 7.62 (d, *J* = 7.6 Hz, 1H), 7.52–7.47 (m, 2H), 7.43–7.37 (m, 3H), 7.33–7.29 (m, 1H), 7.16 (d, *J* = 7.6 Hz, 1H), 2.41 (s, 3H); ^13^C NMR (101 MHz, CDCl_3_) δ 162.6, 141.2, 140.6, 134.8, 134.6, 134.3, 134.1, 133.9, 133.7, 132.1, 131.5, 129.5, 129.1, 128.8, 128.1, 127.8, 127.2, 125.5, 121.5, 121.3, 120.2, 113.3, 21.7; HRMS (MALDI) m/z: [M]^+^ calcd for C_28_H_19_NO 385.1462; found 385.1467.

2-Isopropyl-11-phenyl-6*H*-isoindolo[2,1-*a*]indol-6-one (**2aac**). Pale yellow solid (150.1 mg, 89% yield), mp 141−143 °C. ^1^H NMR (400 MHz, CDCl_3_) δ 7.84 (d, *J* = 7.6 Hz, 1H), 7.72−7.66 (m, 3H), 7.56−7.49 (m, 3H), 7.46−7.42 (m, 1H), 7.35−7.31 (m, 2H), 7.26−7.18 (m, 2H), 2.97−2.89 (m, 1H), 1.26 (d, *J* = 6.8 Hz, 6H); ^13^C NMR (101 MHz, CDCl_3_) δ 162.5, 145.2, 134.8, 134.5, 134.2, 134.0, 133.6, 132.5, 132.2, 129.2, 129.1, 128.7, 128.3, 125.6, 125.3, 121.1, 120.7, 118.8, 113.4, 34.5, 24.5; HRMS (ESI) m/z: [M + H]^+^ calcd for C_24_H_20_NO 338.1539; found 338.1541.

2-Methoxy-11-phenyl-6H-isoindolo[2,1-*a*]indol-6-one (**2aad**) [11]. Pale yellow solid (143.2 mg, 88% yield), mp 193–195 °C. ^1^H NMR (400 MHz, CDCl_3_) δ 7.82 (d, *J* = 8.8 Hz, 1H), 7.73 (d, *J* = 7.6 Hz, 1H), 7.67 (d, *J* = 7.6 Hz, 2H), 7.57–7.50 (m, 3H), 7.46 (t, *J* = 7.6 Hz, 1H), 7.36 (t, *J* = 7.6 Hz, 1H), 7.27 (t, *J* = 7.2 Hz, 1H), 7.00 (s, 1H), 6.90 (d, *J* = 8.8 Hz, 1H), 3.80 (s, 3H); ^13^C NMR (101 MHz, CDCl_3_) δ 162.3, 157.2, 135.2, 135.1, 134.7, 134.1, 133.5, 132.4, 129.2, 129.0, 128.8, 128.5, 128.4, 125.4, 121.2, 120.4, 114.5, 114.2, 105.2, 55.9; HRMS (MALDI) m/z: [M]^+^ calcd for C_22_H_15_NO_2_ 325.1098; found 325.1098.

2-Cyano-11-phenyl-6*H*-isoindolo[2,1-*a*]indol-6-one (**2aae**). Pale yellow solid (107.7 mg, 67% yield), mp 209–211 °C. ^1^H NMR (400 MHz, CDCl_3_) δ 8.04 (d, *J* = 8.4 Hz, 1H), 7.87–7.82 (m, 2H), 7.69–7.58 (m, 6H), 7.54–7.46 (m, 2H), 7.43–7.38 (m, 1H); ^13^C NMR (101 MHz, CDCl_3_) δ 162.5, 135.9, 135.6, 134.5, 134.4, 134.3, 133.5, 131.1, 130.3, 129.9, 129.5, 129.1, 129.0, 126.1, 126.0, 122.0, 120.7, 119.5, 114.2, 107.6; HRMS (MALDI) m/z: [M]^+^ calcd for C_22_H_12_N_2_O 320.0945; found 320.0950.

2-Methoxy-11-(*p*-methoxyphenyl)-6*H*-isoindolo[2,1-*a*]indol-6-one (**2afd**). Pale yellow solid (160.1 mg, 90% yield), mp 170–172 °C. ^1^H NMR (400 MHz, CDCl_3_) δ 7.77 (d, *J* = 8.8 Hz, 1H), 7.68 (d, *J* = 7.2 Hz, 1H), 7.59–7.54 (m, 2H), 7.48 (d, *J* = 7.6 Hz, 1H), 7.33 (t, *J* = 7.6 Hz, 1H), 7.23 (t, *J* = 7.6 Hz, 1H), 7.08–7.03 (m, 2H), 6.95 (d, *J* = 2.4 Hz, 1H), 6.87–6.84 (m, 1H), 3.89 (s, 3H), 3.78 (s, 3H); ^13^C NMR (101 MHz, CDCl_3_) δ 162.2, 159.7, 157.1, 135.2, 134.7, 134.6, 134.0, 133.4, 130.2, 128.5, 128.5, 125.2, 124.5, 121.0, 120.2, 114.6, 114.2, 114.0, 105.2, 55.8, 55.5; HRMS (MALDI) m/z: [M]^+^ calcd for C_23_H_17_NO_3_ 355.1203; found 355.1201.

2-Cyano-11-(*p*-Methoxyphenyl)-6*H*-isoindolo[2,1-*a*]indol-one (**2afe**). Pale yellow solid (118.6 mg, 68% yield), mp 181–183 °C. ^1^H NMR (400 MHz, CDCl_3_) δ 8.01 (d, *J* = 8.8 Hz, 1H), 7.83–7.80 (m, 2H), 7.63–7.56 (m, 4H), 7.50–7.45 (m, 1H), 7.40–7.36 (m, 1H), 7.14–7.09 (m, 2H), 3.93 (s, 3H); ^13^C NMR (101 MHz, CDCl_3_) δ 162.4, 160.3, 135.6, 135.4, 134.6, 134.5, 134.4, 133.4, 130.2, 129.6, 126.0, 125.9, 123.2, 121.8, 119.6, 119.5, 115.0, 114.1, 107.5, 55.6; HRMS (MALDI) m/z: [M]^+^ calcd for C_23_H_14_N_2_O_2_ 350.1050; found 350.1048.

11-(*p*-Methoxyphenyl)-2-(trifluoromethyl)-6*H*-isoindolo[2,1-*a*]indol-6-one (**2aff**). Pale yellow solid (142.3 mg, 72% yield), mp 155–157 °C. ^1^H NMR (400 MHz, CDCl_3_) δ 7.95 (d, *J* = 7.6 Hz, 1H), 7.74–7.71 (m, 2H), 7.59–7.51 (m, 4H), 7.43–7.38 (m, 1H), 7.32–7.27 (m, 1H), 7.11–7.08 (m, 2H), 3.91 (s, 3H); ^13^C NMR (101 MHz, CDCl_3_) δ 162.4, 160.1, 135.3, 135.0, 134.5, 134.1, 134.0, 133.4, 130.2, 129.2, 126.3 (q, *J*_C-F_ = 32.3 Hz), 125.7, 124.6 (q, *J*_C-F_ = 273.7 Hz), 123.6 (q, *J*_C-F_ = 3.0 Hz), 123.5, 121.5, 120.0, 118.6 (q, *J*_C-F_ = 4.0 Hz), 114.8, 113.5, 55.5; ^19^F NMR (376 MHz, CDCl_3_) δ -61.2; HRMS (MALDI) m/z: [M]^+^ calcd for C_23_H_14_F_3_NO_2_ 393.0972; found 393.0969.

9-Methoxy-11-phenyl-6*H*-isoindolo[2,1-*a*]indol-6-one (**2baa**). Pale yellow solid (118.4 mg, 73% yield), mp 173–175 °C. ^1^H NMR (400 MHz, CDCl_3_) δ 7.93 (d, *J* = 8.0 Hz, 1H), 7.69–7.65 (m, 3H), 7.56–7.51 (m, 3H), 7.46–7.42 (m, 1H), 7.31 (t, *J* = 7.6 Hz, 1H), 7.15 (t, *J* = 7.6 Hz, 1H), 7.07 (s, 1H), 6.75 (d, *J* = 8.4 Hz, 1H), 3.78 (s, 3H); ^13^C NMR (101 MHz, CDCl_3_) δ 164.4, 162.5, 136.8, 133.9, 133.8, 133.7, 132.4, 129.1, 129.1, 128.4, 127.1, 126.9, 126.3, 123.8, 121.3, 120.4, 113.6, 113.4, 107.5, 55.8; HRMS (MALDI) m/z: [M]^+^ calcd for C_22_H_15_NO_2_ 325.1098; found 325.1103.

9-Fluoro-11-phenyl-6*H*-isoindolo[2,1-*a*]indol-6-one (**2caa**). Pale yellow solid (120.7 mg, 77% yield), mp 195–197 °C. ^1^H NMR (400 MHz, CDCl_3_) δ 7.94 (d, *J* = 8.0 Hz, 1H), 7.77–7.72 (m, 1H), 7.68–7.50 (m, 2H), 7.59–7.53 (m, 3H), 7.50–7.46 (m, 1H), 7.37–7.32 (m, 1H), 7.26–7.17 (m, 2H), 7.00–6.94 (m, 1H); ^13^C NMR (101 MHz, CDCl_3_) δ 166.5 (d, *J*_C-F_ = 254.5 Hz), 161.6, 137.1 (d, *J*_C-F_ = 11.1 Hz), 134.0, 133.8, 132.9 (d, *J*_C-F_ = 4.0 Hz), 131.9, 129.9, 129.3, 129.0, 128.8, 127.6, 127.5 (d, *J*_C-F_ = 11.1 Hz), 124.3, 121.7, 121.6, 115.9 (d, *J*_C-F_ = 24.2 Hz), 113.6, 109.1 (d, *J*_C-F_ = 25.3 Hz); ^19^F NMR (376 MHz, CDCl_3_) δ -103.9; HRMS (MALDI) m/z: [M]^+^ calcd for C_21_H_12_FNO 313.0898; found 313.0896.

### 3.5. Typical Experimental Procedure for the Synthesis of (Z)-3-Benzylidene-2-Phenylisoindolin-1-One (**2a**)

A mixture of *N*-phenyl-*ortho*-(phenylethynyl)benzamide (**1aaa**, 297.4 mg, 1.0 mmol), CuI (9.5 mg, 0.05 mmol), and L-proline (17.3 mg, 0.15 mmol) in DMF (2.0 mL) in a 25 mL screw-capped thick-walled Pyrex tube was stirred at 80 °C for 6 h under nitrogen atmosphere. After the reaction mixture was cooled to room temperature, it was then poured into a solvent mixture of water (15.0 mL) and ethyl acetate (20.0 mL), and the two phases were then separated. The aqueous layer was extracted with ethyl acetate (3 × 20.0 mL). After the combined organic extracts were dried over MgSO_4_ overnight, the filtered solution was concentrated under reduced pressure. The crude product was purified by column chromatography on silica gel with the use of petroleum ether/ethyl acetate as eluent to afford **2a** as a white solid (273.6 mg, 92%).

### 3.6. Characterization Data of Intermediates **2a**, **2d** and **2d**-**d_5_**

(*Z*)-3-Benzylidene-2-phenylisoindolin-1-one (**2a**) [25]. White solid, mp 198–200 °C. ^1^H NMR (400 MHz, CDCl_3_) δ 7.94 (d, *J* = 7.6 Hz, 1H), 7.84 (d, *J* = 7.6 Hz, 1H), 7.66 (t, *J* = 8.0 Hz, 1H), 7.53 (t, *J* = 7.6 Hz, 1H), 7.09–7.04 (m, 5H), 6.97–6.89 (m, 3H), 6.86–6.82 (m, 3H); ^13^C NMR (101 MHz, CDCl_3_) δ 168.0, 138.8, 136.0, 134.4, 133.7, 132.5, 129.3, 129.2, 128.3, 127.9, 127.3, 126.8, 126.7, 124.0, 119.5, 107.7; HRMS (ESI) m/z: [M + H]^+^ calcd for C_21_H_16_NO 298.1226; found 298.1230.

(*Z*)-3-(*p*-Methylbenzylidene)-2-phenylisoindolin-1-one (**2d**). White solid (283.7 mg, 91% yield), mp 187–189 °C. ^1^H NMR (400 MHz, CDCl_3_) δ 7.93 (d, *J* = 7.6 Hz, 1H), 7.82 (d, *J* = 7.6 Hz, 1H), 7.66–7.61 (m, 1H), 7.53–7.48 (m, 1H), 7.10–7.05 (m, 5H), 6.78 (s, 1H), 6.74–6.69 (m, 4H), 2.17 (s, 3H); ^13^C NMR (101 MHz, CDCl_3_) δ 168.0, 138.9, 136.6, 136.1, 133.8, 132.4, 130.7, 129.2, 129.1, 128.2, 128.0, 127.8, 127.3, 126.6, 123.9, 119.4, 108.0, 21.2; HRMS (ESI) m/z: [M + H]^+^ calcd for C_22_H_18_NO 312.1383; found 312.1387.

(*Z*)-3-(*p*-Methylbenzylidene)-2-(phenyl-*d_5_*)isoindolin-1-one (**2d**-*d_5_*). White solid (0.5 mmol-scale, 145.5 mg, 92% yield), mp 188−190 °C. ^1^H NMR (400 MHz, CDCl_3_) δ 7.93 (d, *J* = 7.6 Hz, 1H), 7.81 (d, *J* = 8.0 Hz, 1H), 7.63 (t, *J* = 7.6 Hz, 1H), 7.51 (t, *J* = 7.6 Hz, 1H), 6.78 (s, 1H), 6.74−6.69 (m, 4H), 2.17 (s, 3H); HRMS (ESI) m/z: [M + H]^+^ calcd for C_22_H_13_D_5_NO 317.1697; found 317.1671.

## 4. Conclusions

In summary, in the presence of CuI and Pd(OAc)_2_, *ortho*-alkynyl-*N*-arylbenzamides undergo a stepwise intramolecular hydroamidation of alkynyl group, and C-H dehydrogenative coupling reaction in oxygen atmosphere to give isoindolo[2,1-*a*]indol-6-one derivatives under mild reaction conditions. The reaction conditions show a high tolerance to a variety of functional groups such as Cl, F, CF_3_, CN and OMe, some of them being useful for further transformations.

## Figures and Tables

**Table 1:** Optimizing reaction conditions for hydroamidation *^a^*.

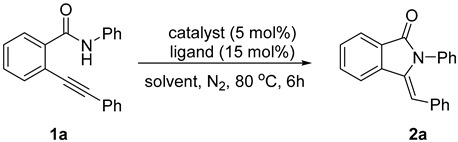				
Entry	Catalyst	Ligand	Solvent	Yield (%) *^b^*
1	FeCl_3_	-	DMF	0
2	Cu(OAc)_2_	-	DMF	0
3	Pd(OAc)_2_	-	DMF	0
4	CuI	-	DMF	38
5	CuI	-	DMSO	35
6	CuI	-	MeCN	18
7	CuI	-	NMP	<5
8	CuI		1,4-dioxane	12
9	CuI	-	toluene	20
10	CuI	1,10-phen	DMF	72
11	CuI	TMEDA	DMF	51
12	CuI	L-proline	DMF	92
13	CuCl	L-proline	DMF	80
14	CuBr	L-proline	DMF	82

*^a^* Reactions were carried out using **1a** (0.5 mmol), catalyst (5 mol %, 0.025 mmol), ligand (15 mol %, 0.075 mmol), solvent (1.0 mL), in N_2_, at 80 °C for 6 h. *^b^* Isolated yields.

**Table 2:** Optimizing conditions for C-H oxidative dehydrocoupling reaction *^a^*.

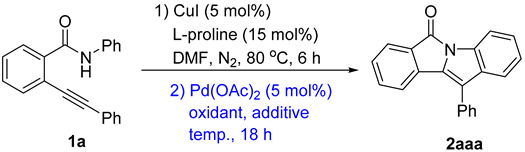				
Entry	Oxidant	Additive	Temp (°C)	Yield (%) *^b^*
1	O_2_	HOAc	80	51
2 *^c^*	1,4-benzoquinone	HOAc	80	40
3 *^c^*	K_2_S_2_O_8_	HOAc	80	<5
4 *^c^*	Cu(OAc)_2_	HOAc	80	16
5 *^d^*	O_2_	*p*-TsOH	80	78
6	O_2_	TFA	80	83
7	O_2_	TFA	100	76
8	O_2_	TFA	60	58

*^a^* Reaction conditions: (1) **1a** (0.5 mmol), CuI (5 mol %, 0.025 mmol), L-proline (15 mol %, 0.075 mmol), DMF (1.0 mL), in N_2_, at 80 °C for 6 h; (2) Pd(OAc)_2_ (5 mol %), O_2_ (1 atm), additional solvent (1.0 mL), temp., 18 h. *^b^* Isolated yields. *^c^* Oxidant (3.0 equiv). *^d^* *p*-TsOH (~30 equiv).

## Data Availability

Not applicable.

## Supplementary Materials

The following are available online at https://www.mdpi.com/article/10.3390/molecules27113393/s1: the typical procedure for the synthesis of starting materials [26,27], the copies of NMR charts of new starting materials, and all products, as well as X-ray structural details of **2a**.

## Sample Availability

Samples of the final products are not available from the authors.
