# Supplementary material for: Cu(I)/Pd(II)-Catalyzed Intramolecular Hydroamidation and C-H Dehydrogenative Coupling of ortho-Alkynyl-N-arylbenzamides for Access to Isoindolo[2,1-a]Indol-6-Ones
Source: Molecules. 2022 May 25;27(11):3393. doi: 10.3390/molecules27113393 (PMC9181885; doi:10.3390/molecules27113393)
Supplement: Supplementary file 1 [file molecules-27-03393-s001.zip › molecules-1718846-supplementary.pdf]

Supplementary File

Cu(I)/Pd(II)-Catalyzed Intramolecular Hydroamidation and C-H Dehydrogenative Coupling of *ortho*-Alkynyl-*N*-arylbenzamides for Access to Isoindolo[2,1-*a*]indol-6-ones

Baoxin Tang [1] and Ruimao Hua [1,2,*]

[1] Department of Chemistry, Tsinghua University, Key Laboratory of Organic Optoelectronics & Molecular Engineering of Ministry of Education, Beijing 100084, China

[2] College of Chemistry, Xinjiang University, Urumqi 830017, China

Contents Page

1. The structures of starting materials S2
2. Typical procedure for synthesis of *ortho*-arylethynyl-*N*-arylbenzamides S3
3. ^{1}H and ^{13}C NMR charts of the starting materials (new compounds) S5
4. ^{1}H-, ^{13}C-, and ^{19}F NMR charts of the products S27
5. ^{1}H and ^{13}C NMR charts of the intermediates S60
6. X-ray structural details of **2a** S64

1. The structure of starting materials

All the substrates of *ortho*-(1-arylethynyl)-*N*-arylbenzamides were prepared according to a modified literature methods [1]. **1aaa** [2], **1ada** [2], **1afa** [3], **1aab** [2], **1aad** [2], **1afd** [1] are known compounds.

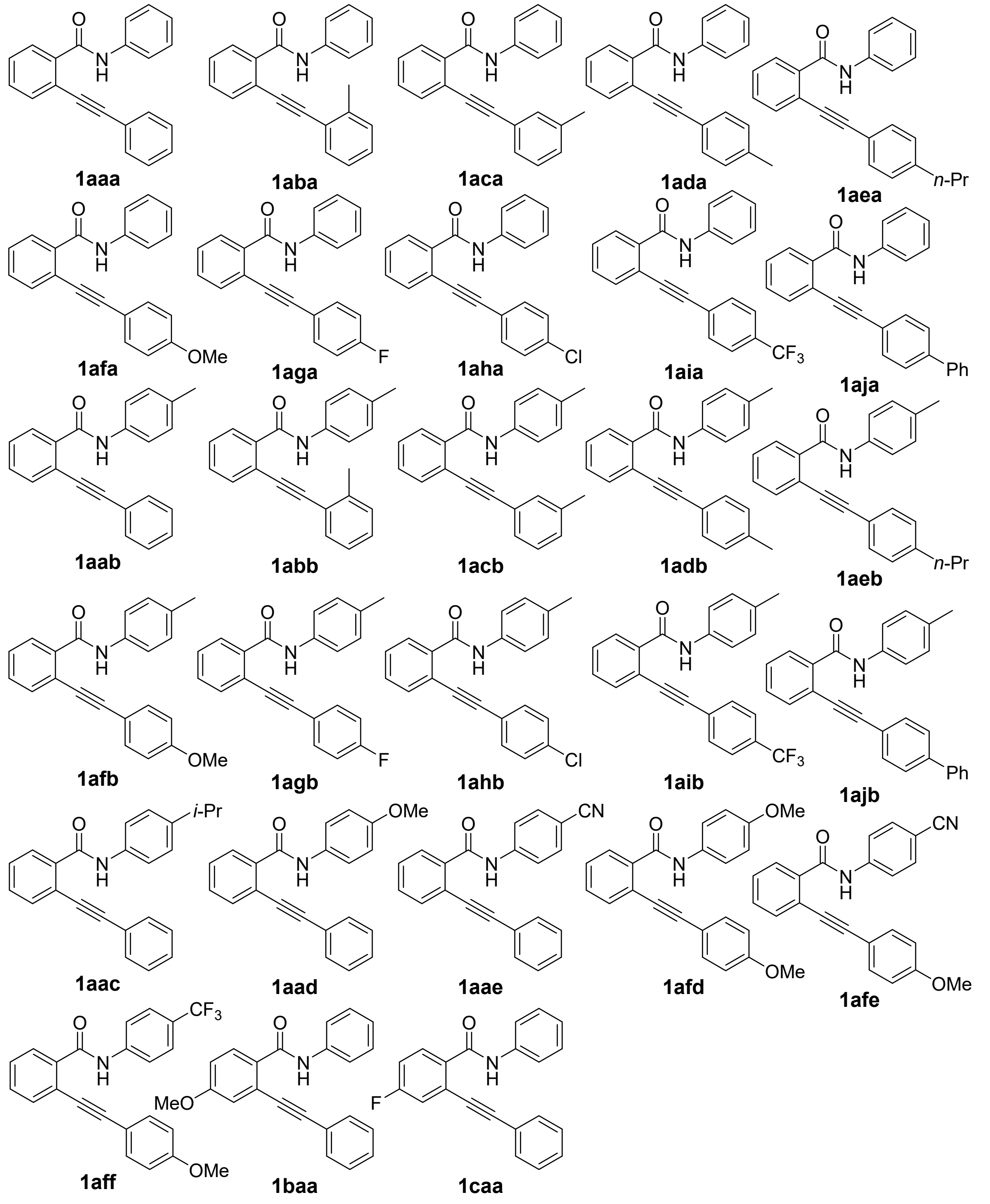

2. Typical procedure for synthesis of *ortho*-arylethynyl-*N*-arylbenzamides [1]

(1) Typical procedure for synthesis of 2-iodo-*N*-arylbenzamide according to a modified literature method [4].

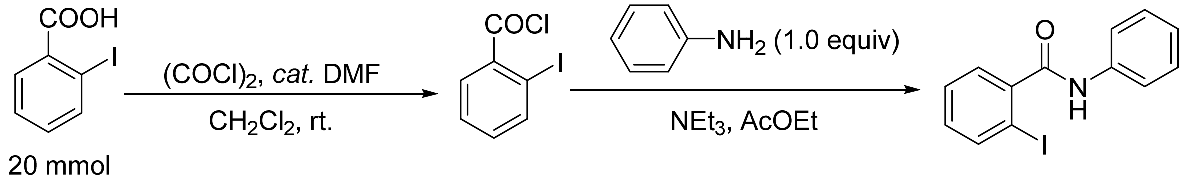

Step 1: To a solution of the 2-iodobenzoic acid (4.960 g, 20.0 mmol) in CH_2Cl_2 (40 mL) was added 5 drops of DMF. Oxalylchloride (3.046 g, 24 mmol) was then added dropwise. The resulting mixture was stirred for 2 hours at room temperature, and solvent and excess oxalyl chloride were removed *in vacuo* to afford 2-iodobenzoyl chloride as a yellow solid in 99% yield (5.266 g). The crude product was used in the next step without further purification.

Step 2: To a solution of the 2-iodobenzoyl chloride (2.665 g, 10.0 mmol) in ethyl acetate (25 mL) was added NEt_3 (1.012 g, 10.0 mmol), then aniline (0.931 g, 10.0 mmol). The resulting mixture was stirred at room temperature overnight. After the reaction, water (25 mL) was added, and the mixture was extracted with ethyl acetate (3 x 25 mL). The combined organic phases were dried over $MgSO_4$. The filtered solution was concentrated under reduced pressure to afford 2-iodo-*N*-phenylbenzamide as a white solid in 95% yield (3.070 g). The crude product was used in the next step without further purification.

(2) Typical procedure for synthesis of *ortho*-arylethynyl-*N*-arylbenzamide according to a modified literature method [1].

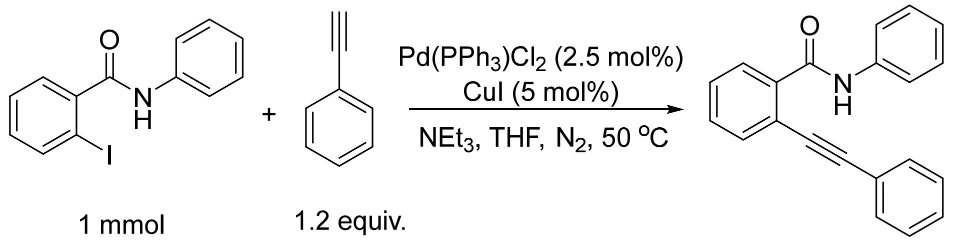

A mixture of 2-iodo-*N*-phenylbenzamide (646.3 mg, 2.0 mmol), $Pd(PPh_3)_2Cl_2$ (35.1 mg, 2.5 mol%), CuI (19.0 mg, 5 mol%) in NEt_3 (2 mL) and THF (2 mL), the mixture was stirred for 10 minutes under nitrogen atmosphere. Then ethynylbenzene (245.1 mg, 2.4 mmol, 1.2 eq.) was added to the solution and heated at 50 °C overnight. The reaction mixture was diluted with water (25 mL) and the aqueous phase was extracted with EtOAc (3 x 25 mL). The combined organic phases were dried over $MgSO_4$.The filtered solution was concentrated

under reduced pressure. The he crude residue was purified by column chromatography on silica gel with the use of petroleum ether/ethyl acetate (gradient mixture ratio from 12:1 to 4:1 in volume) to afford **1aaa** as a white solid in 95% yield (566.0 mg).

[1] Yao, B.; Jaccoud, C.; Wang, Q.; Zhu, J. Synergistic effect of palladium and copper catalysts: catalytic cyclizative dimerization of *ortho*-(1-alkynyl)benzamides leading to axially chiral 1,3-butadienes. *Chem. Eur. J.* **2012**, *18*, 5864–5868.

[2] Dev, K, Maurya R. Facile synthesis of 11-aryl-6*H*-isoindolo[2,1-a]indol-6-ones via hypervalent iodine(III)-promoted cascade cyclization. *RSC Adv.* **2015**, *5*, 13102–13106.

[3] Madich, Y.; Álvarez, R.; Aurrecoechea, J. M. Palladium-catalyzed regioselective 5-exo-O-cyclization/oxidative heck cascades from o-alkynylbenzamides and electron-deficient alkenes. *Eur. J. Org. Chem.*, **2014**, 6263–6271.

[4] Dothager, R. S.; Putt, K. S.; Allen, B. J.; Leslie, B. J.; Nesterenko, V.; Hergenrother, P. J. Synthesis and identification of small molecules that potently induce apoptosis in melanoma cells through G1 cell cycle arrest. *J. Am. Chem. Soc.* **2005**, 127, 8686–8696.

3. ^{1}H and ^{13}C NMR charts of the starting materials (new compounds)

^{1}H NMR spectrum of **1aba**

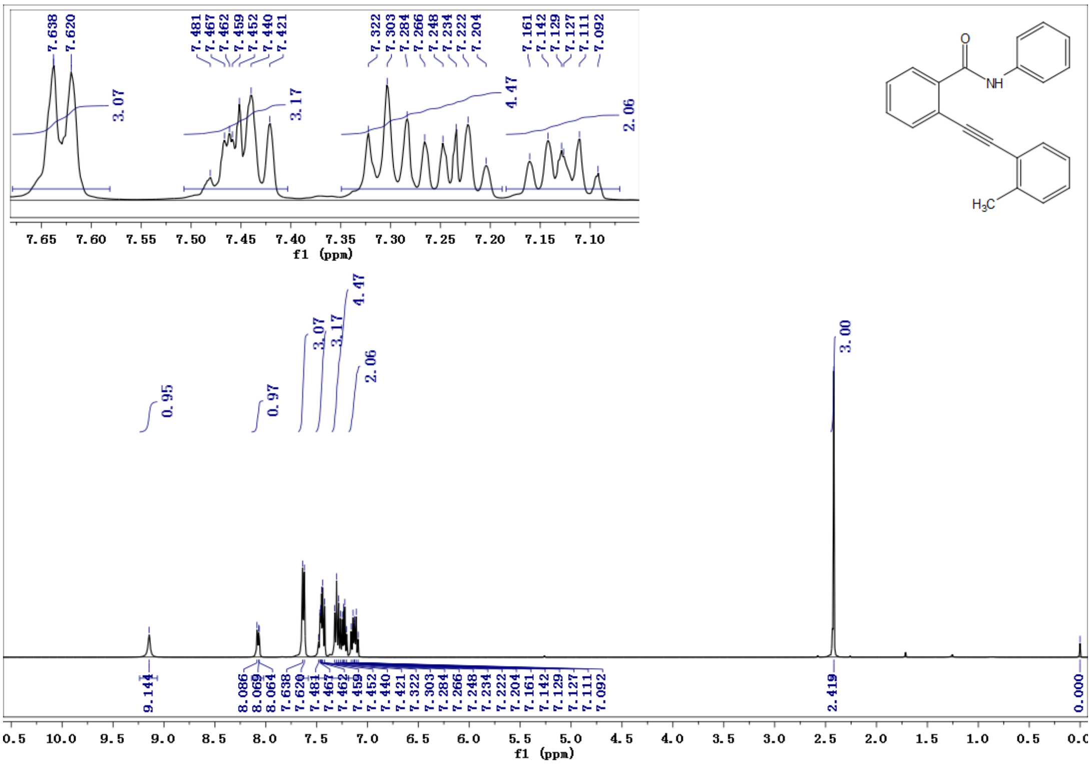

^{13}C NMR spectrum of **1aba**

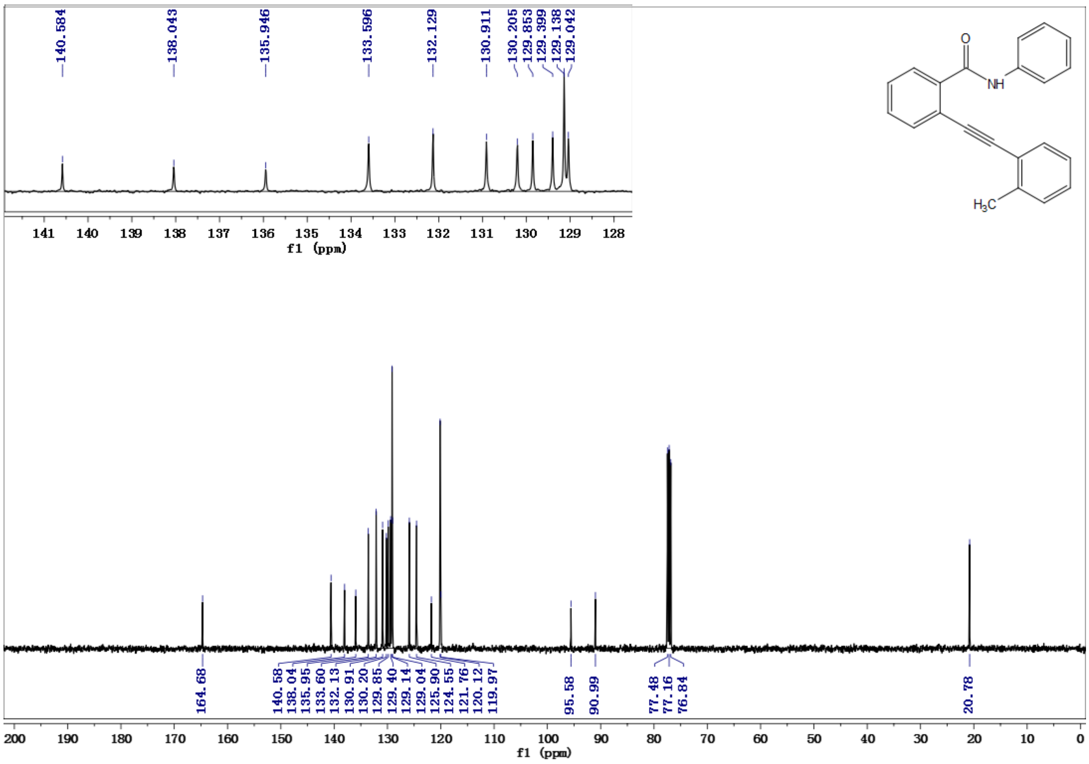

^{1}H NMR spectrum of **1aca**

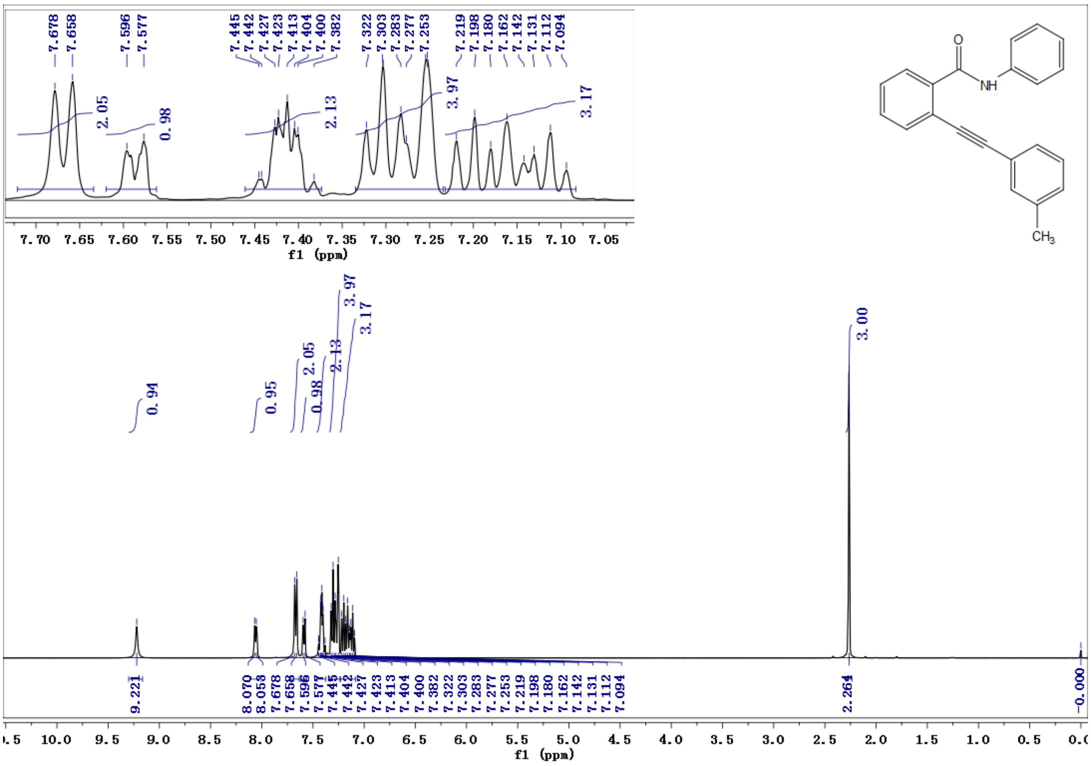

^{13}C NMR spectrum of **1aca**

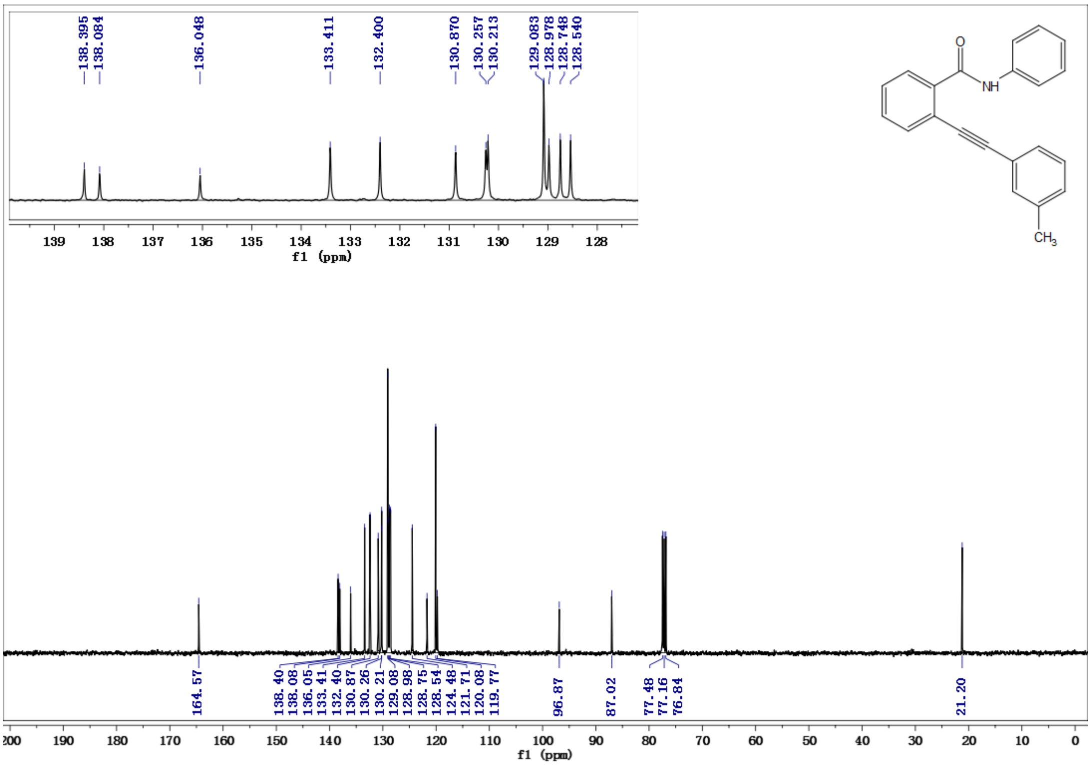

^{1}H NMR spectrum of **1aea**

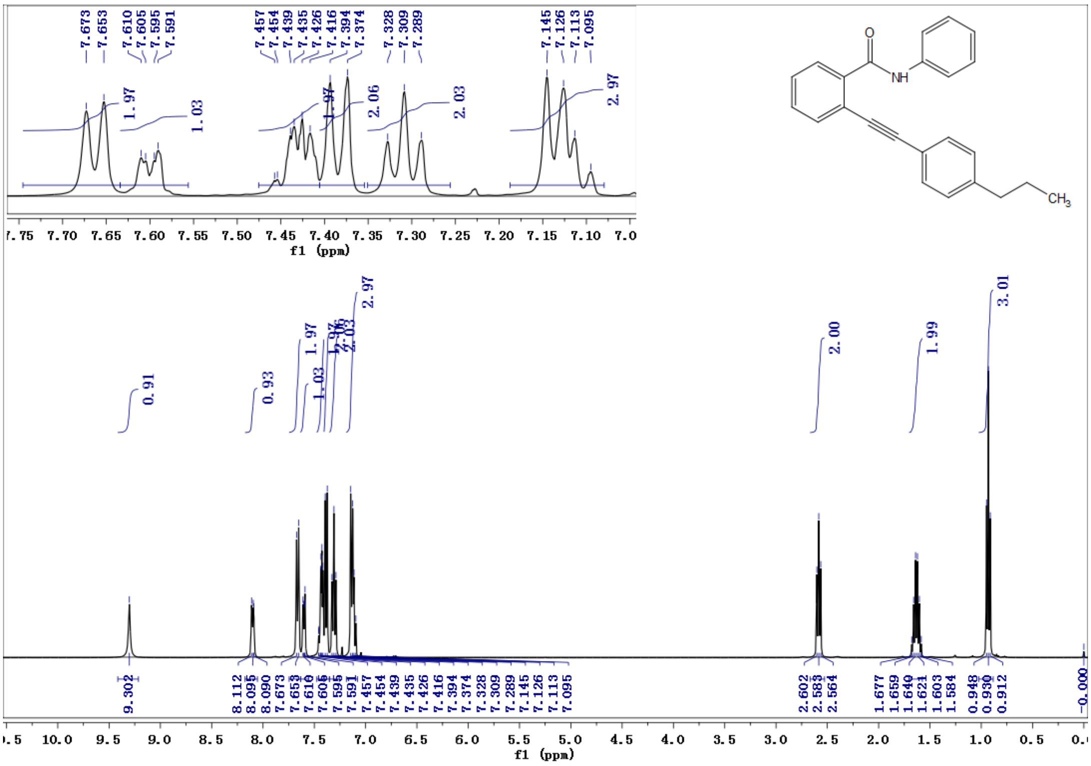

^{13}C NMR spectrum of **1aea**

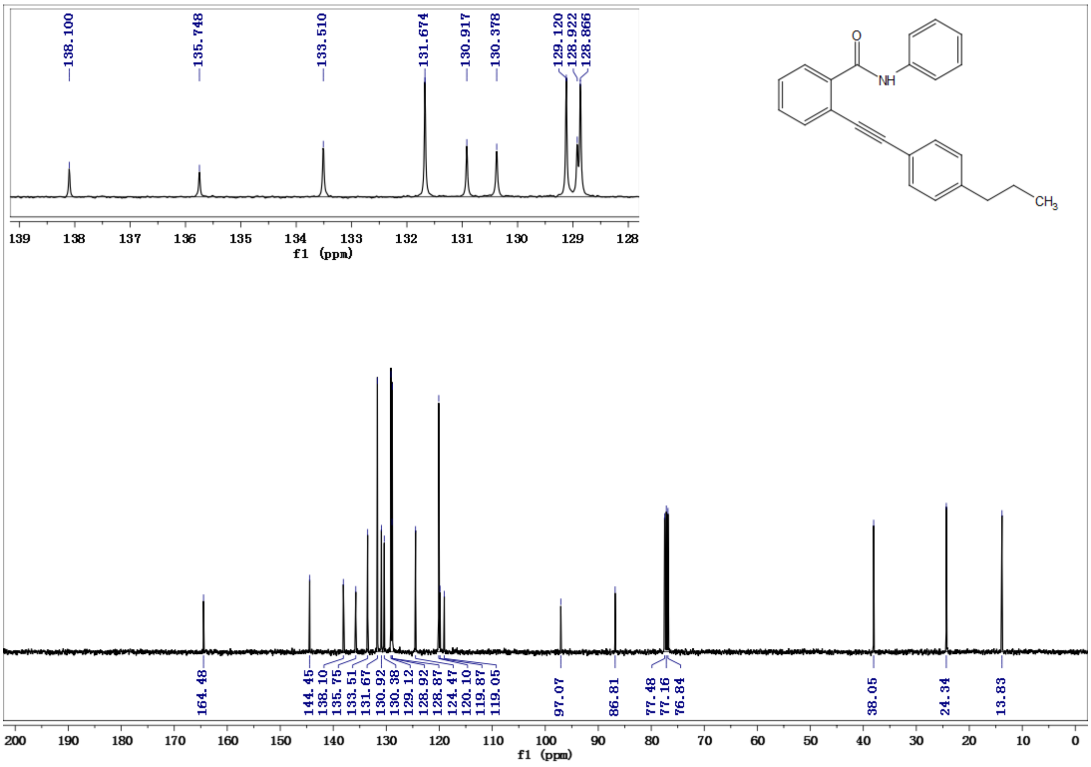

^{1}H NMR spectrum of **1aga**

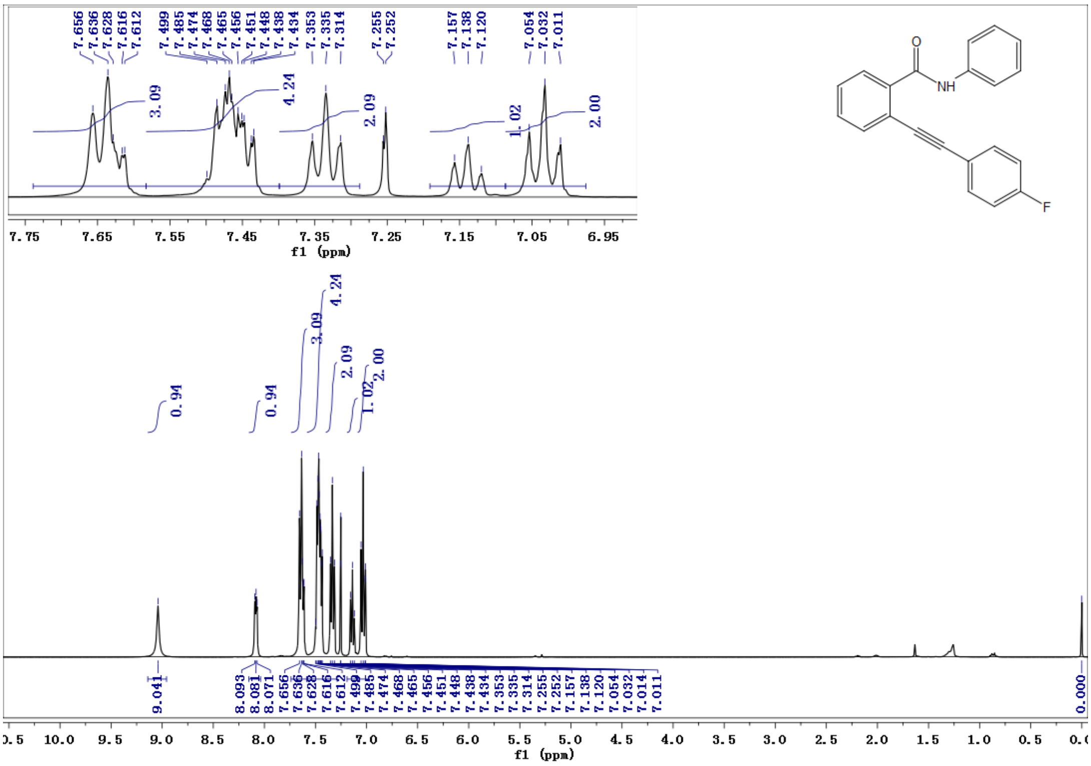

^{13}C NMR spectrum of **1aga**

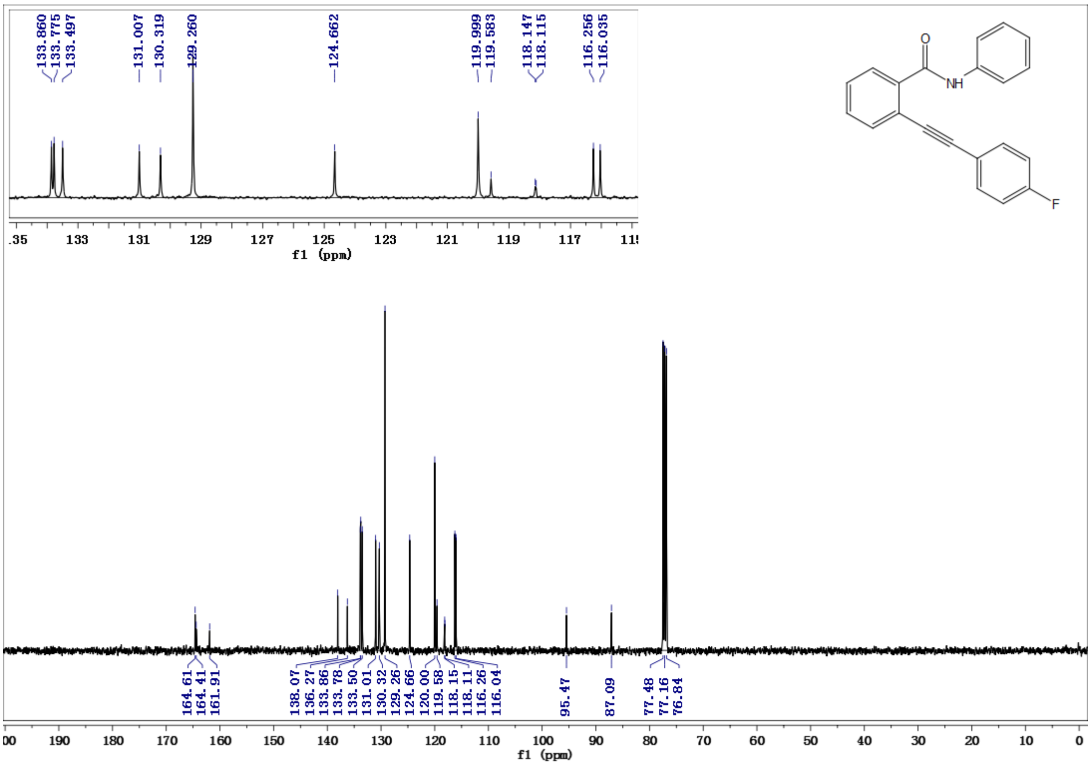

^{1}H NMR spectrum of **1aha**

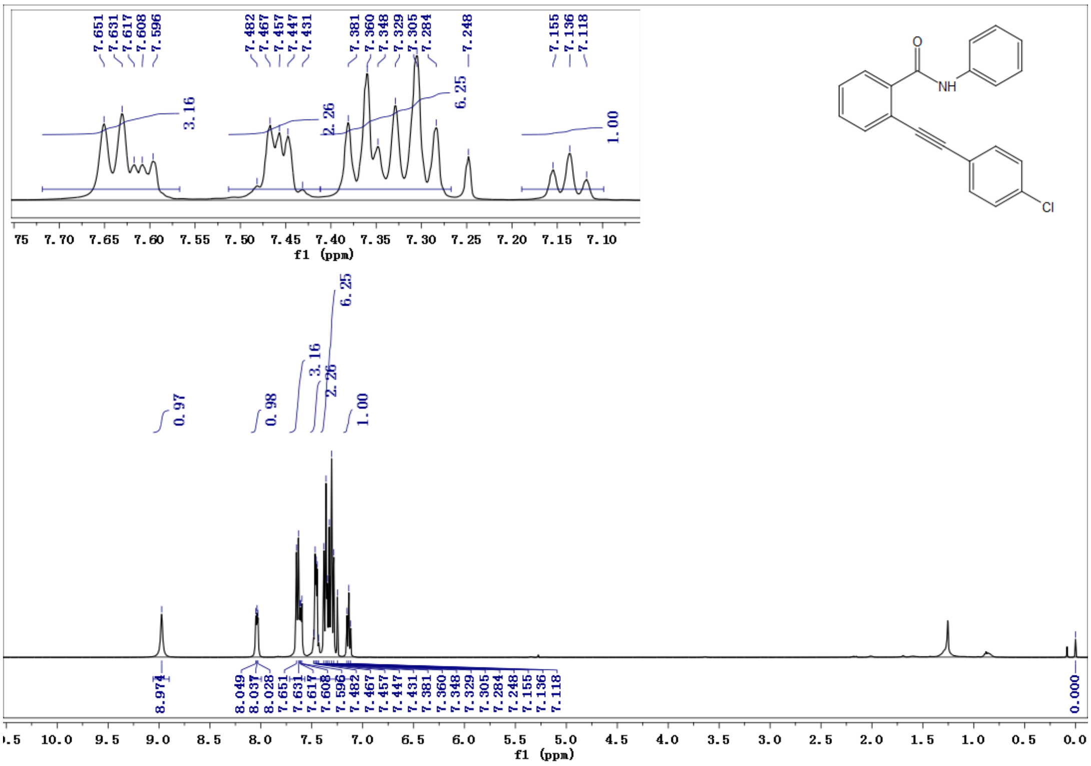

^{13}C NMR spectrum of **1aha**

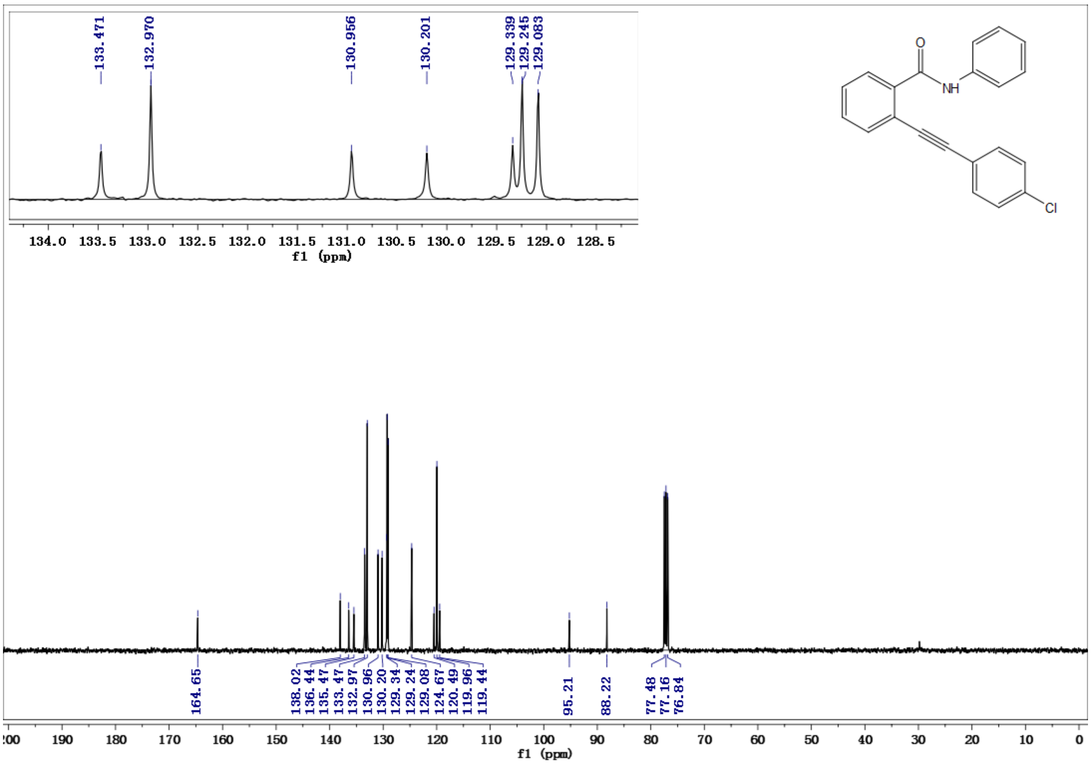

^{1}H NMR spectrum of **1aia**

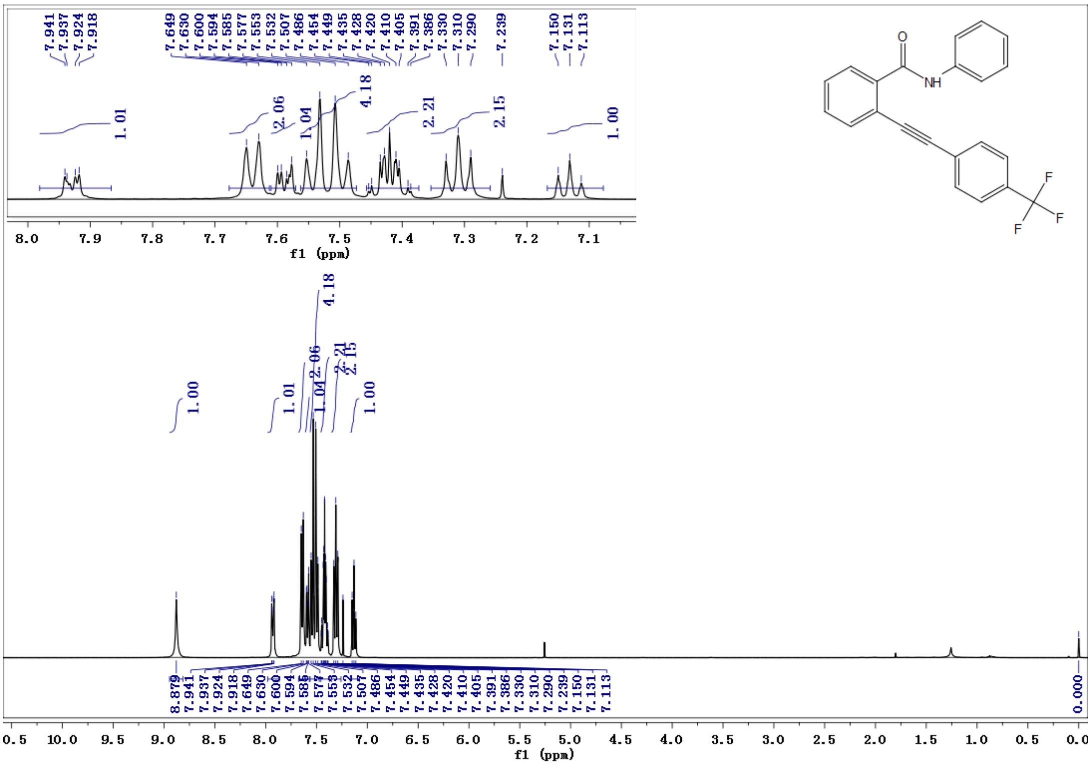

^{13}C NMR spectrum of **1aia**

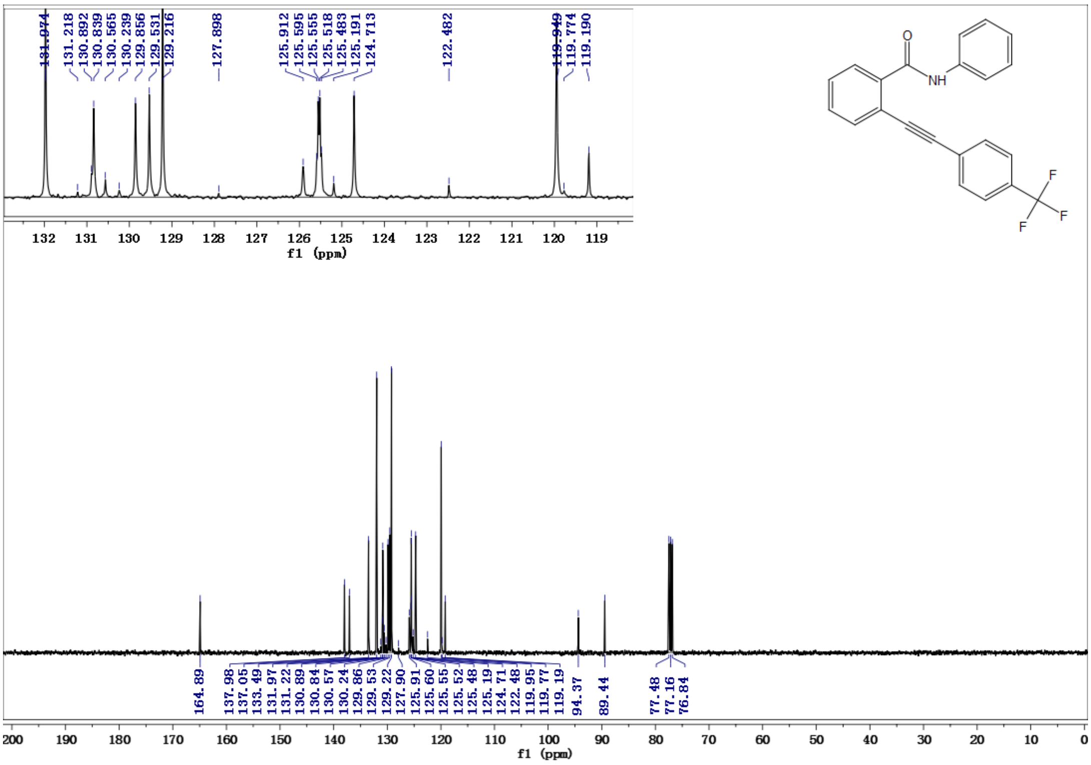

^{1}H NMR spectrum of **1aja**

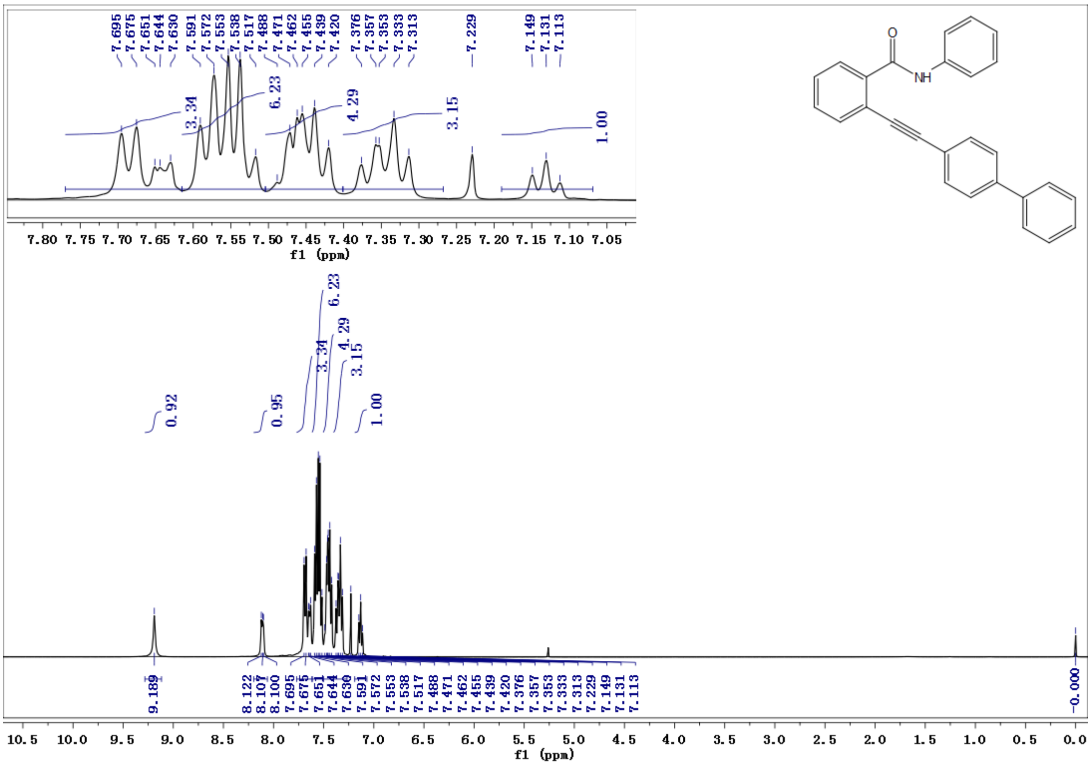

^{13}C NMR spectrum of **1aja**

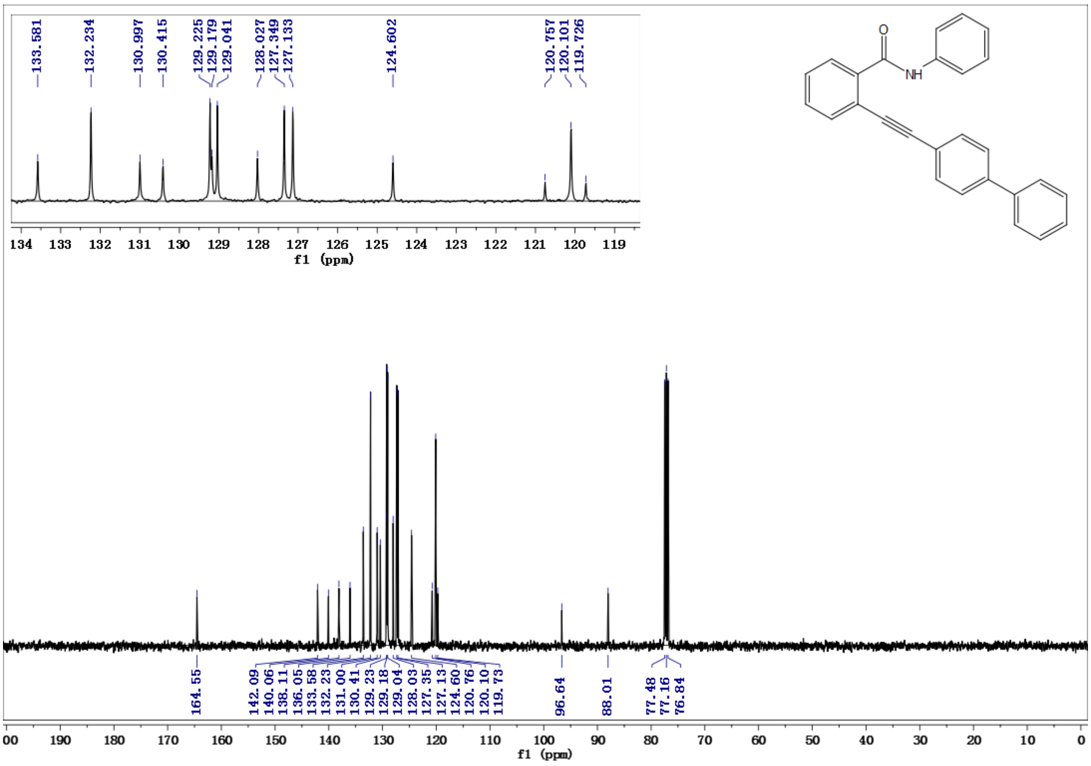

^{1}H NMR spectrum of **1abb**

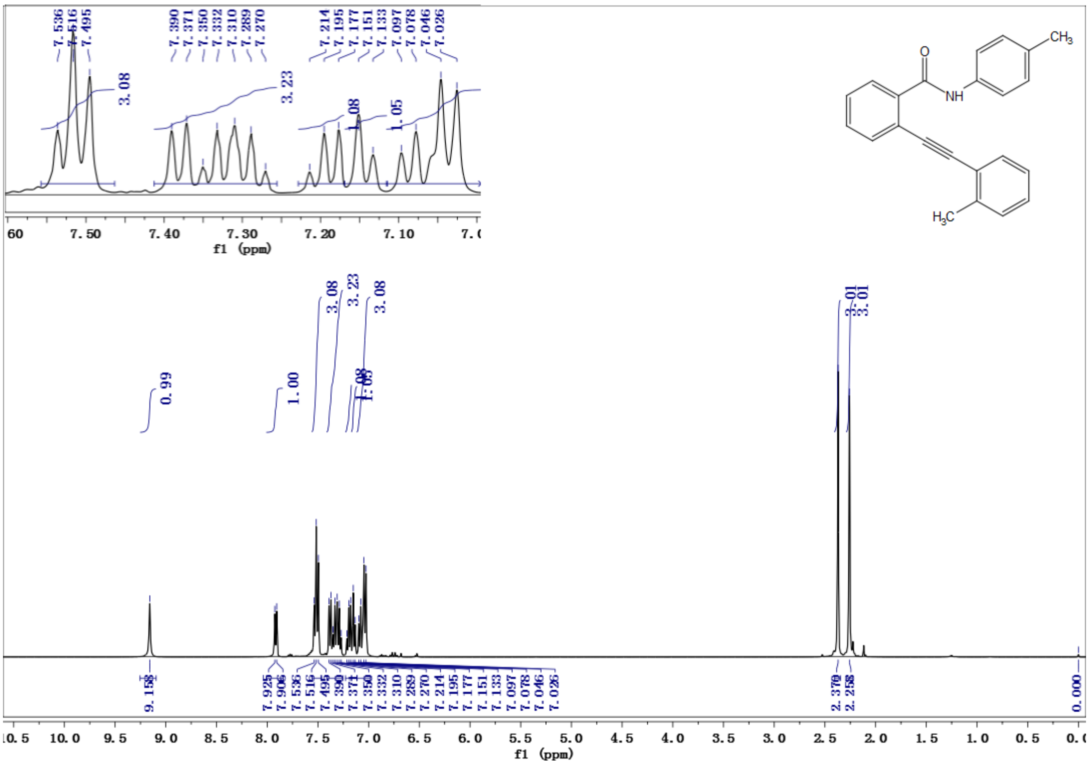

^{13}C NMR spectrum of **1abb**

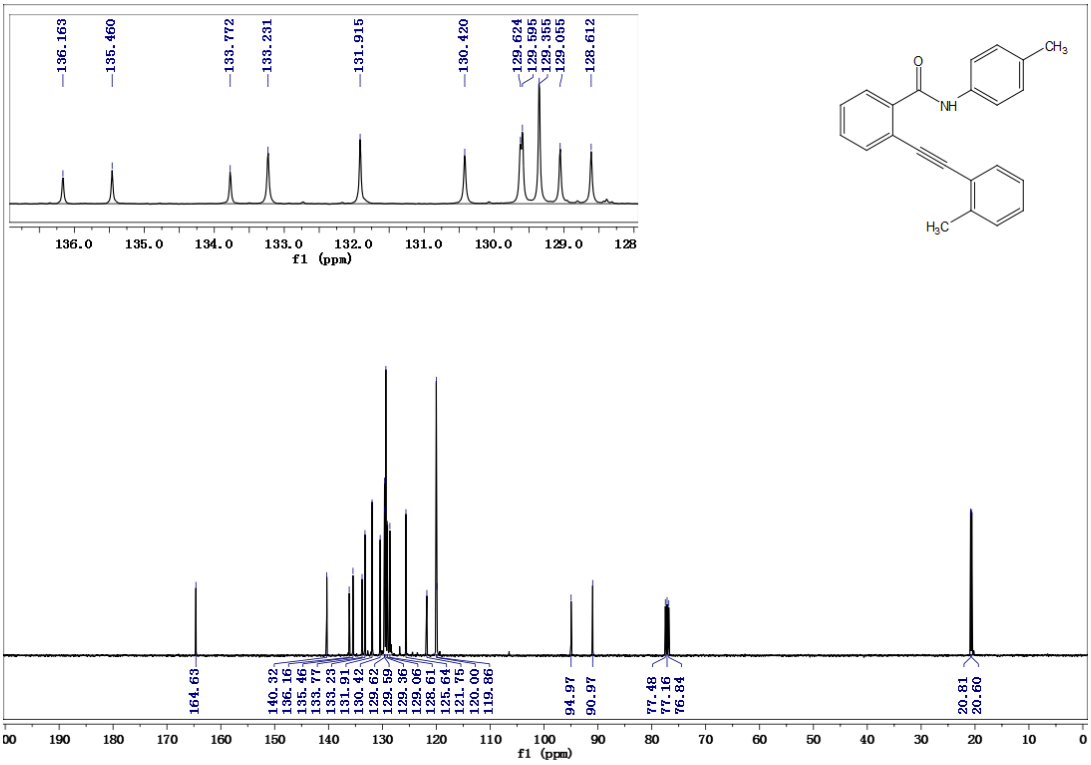

^{1}H NMR spectrum of **1acb**

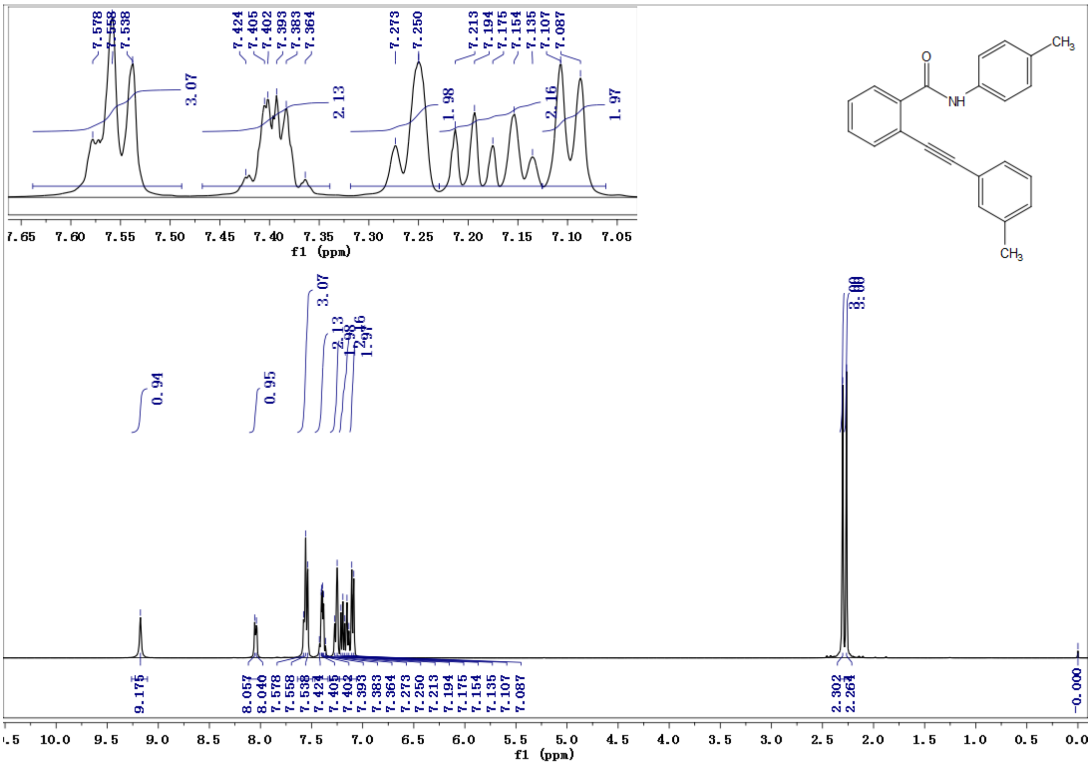

^{13}C NMR spectrum of **1acb**

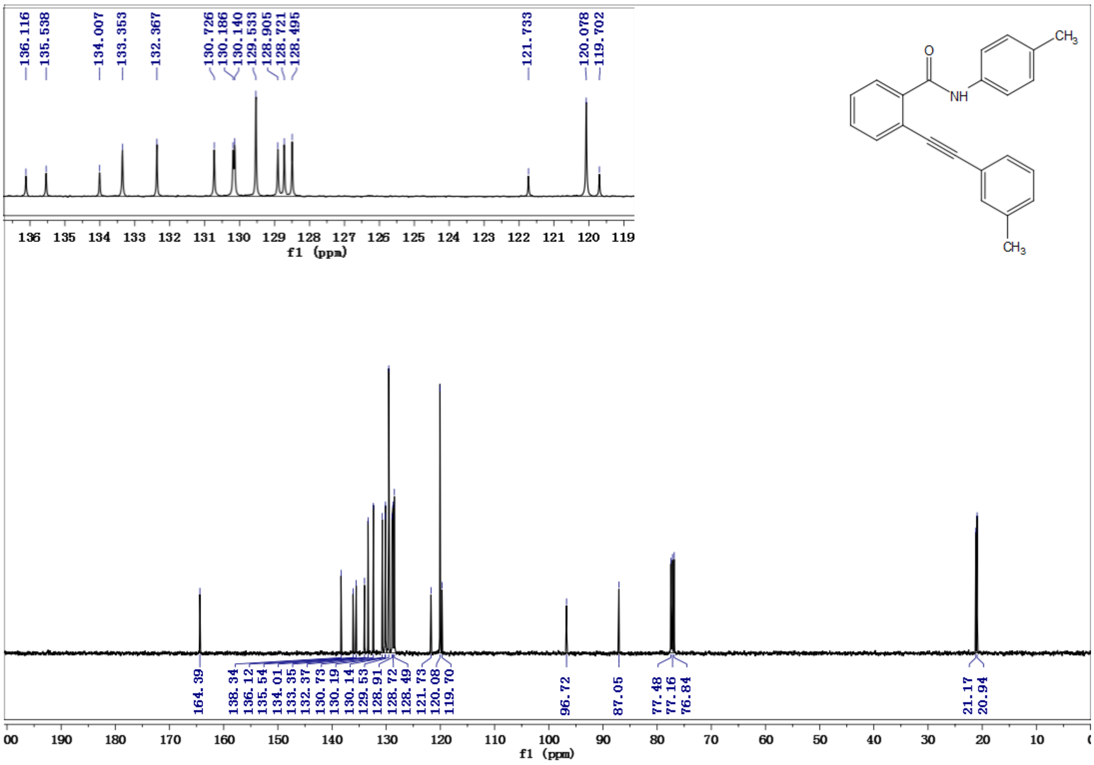

^{1}H NMR spectrum of **1adb**

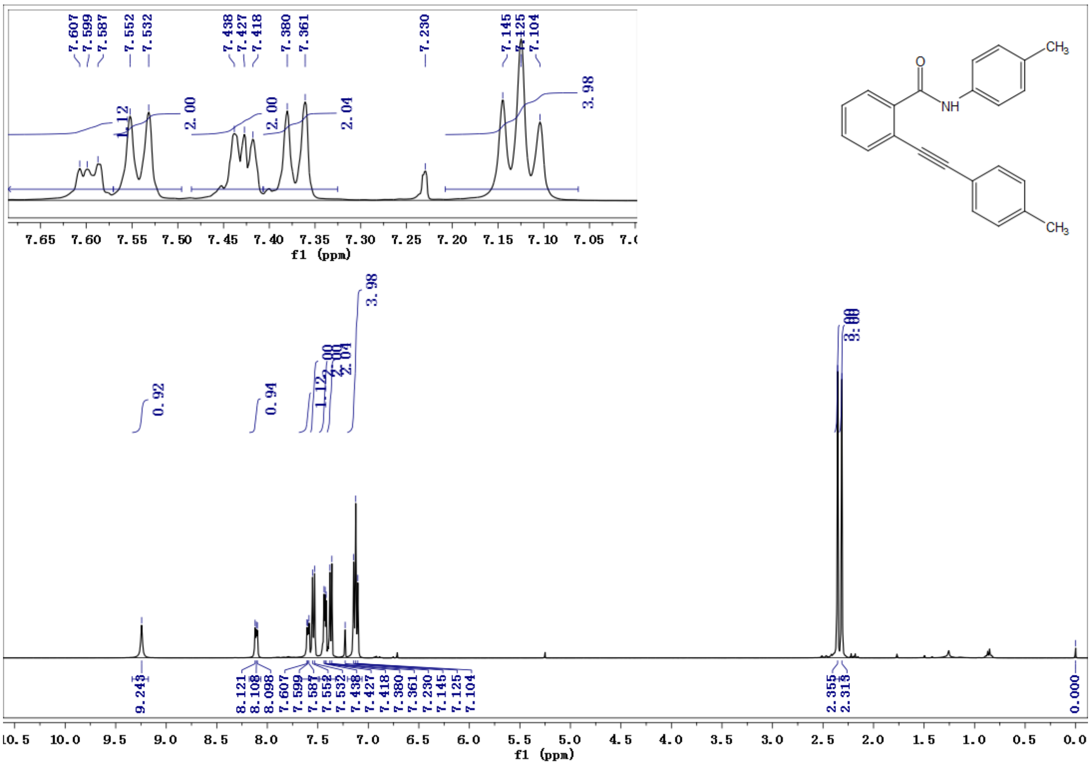

^{13}C NMR spectrum of **1adb**

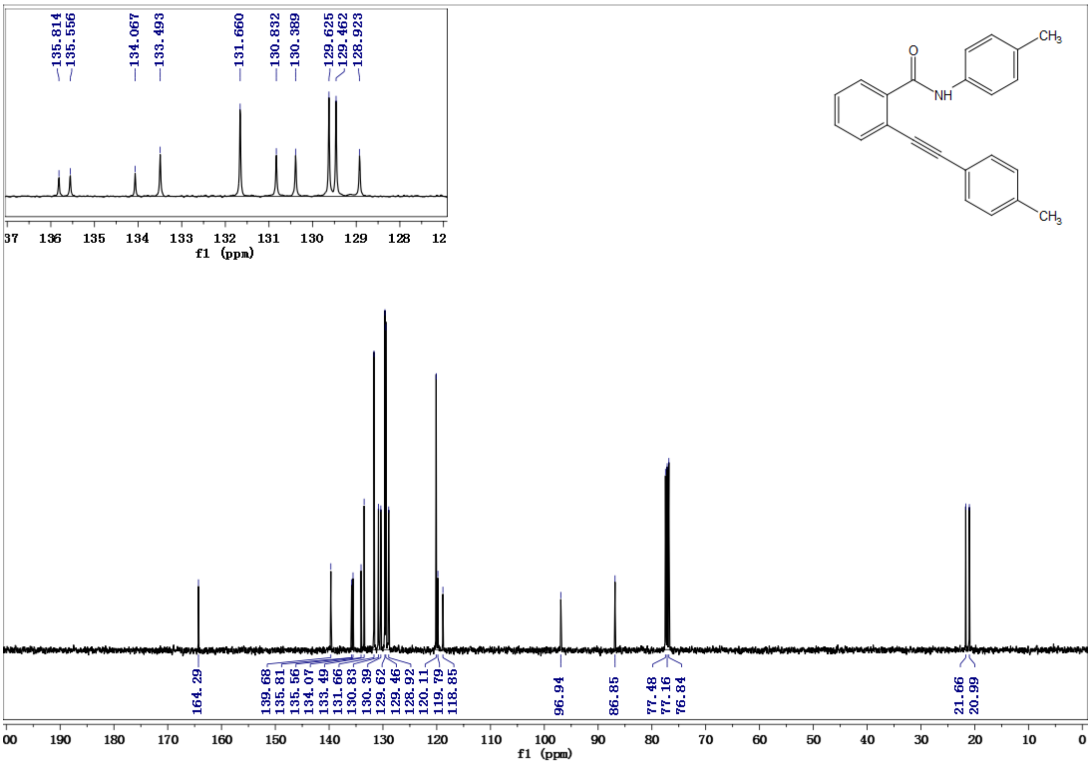

^{1}H NMR spectrum of **1aeb**

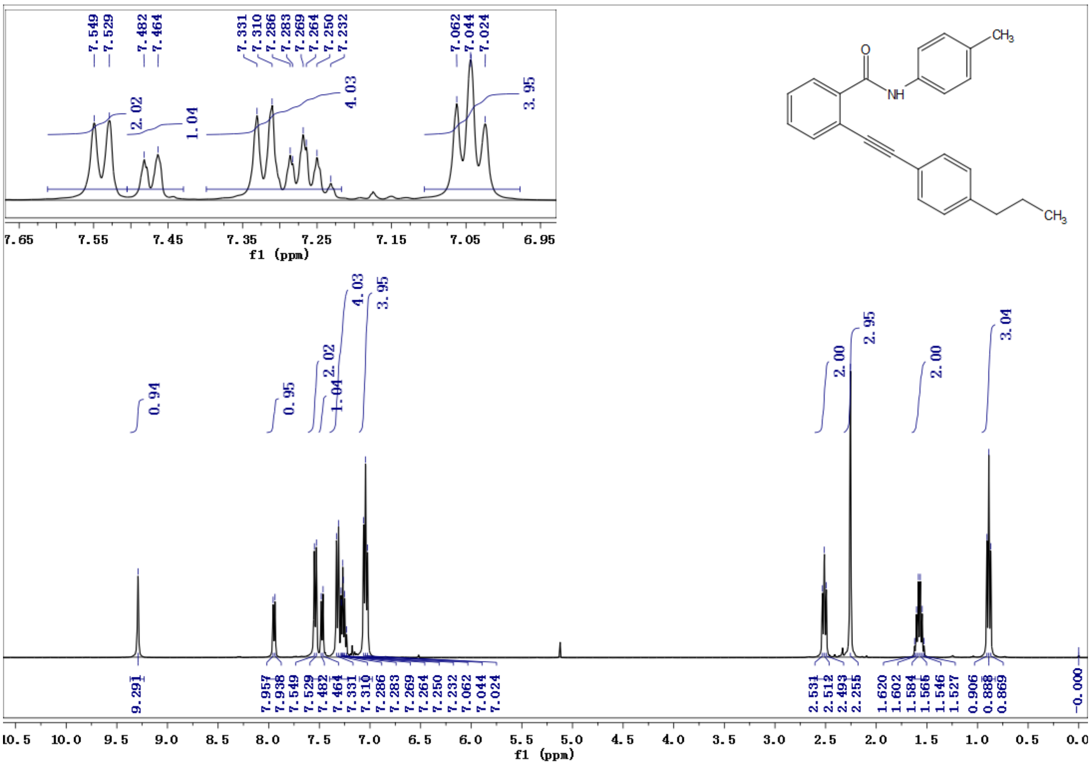

^{13}C NMR spectrum of **1aeb**

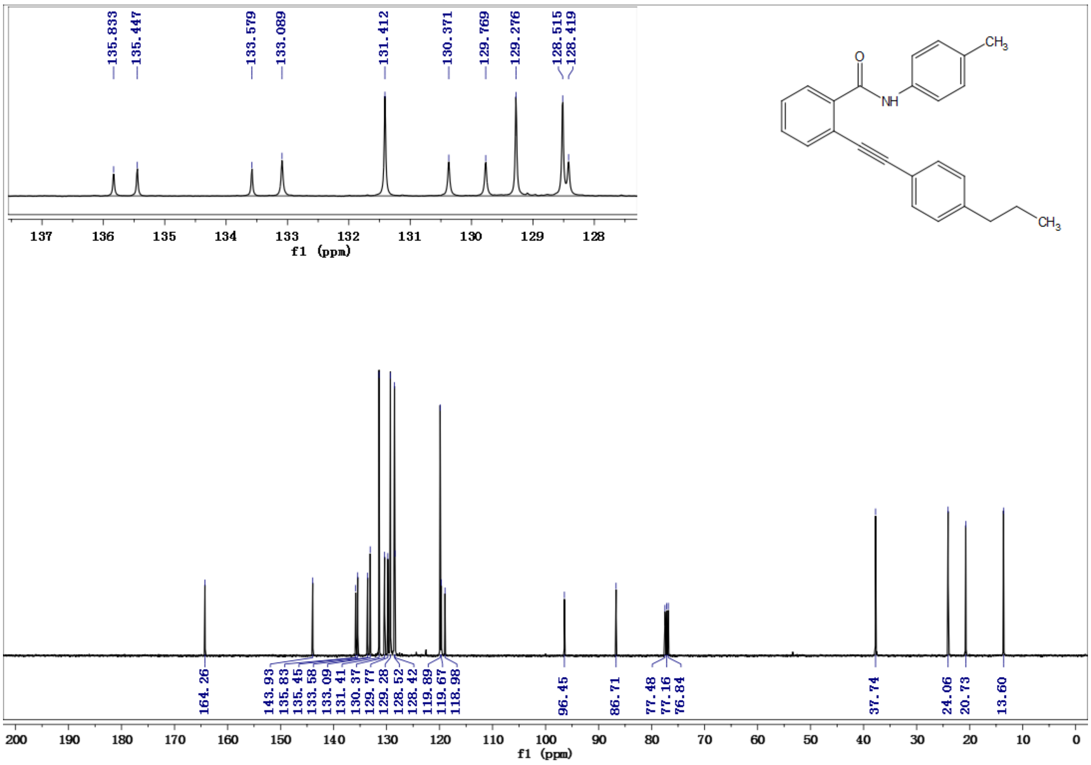

^{1}H NMR spectrum of **1afb**

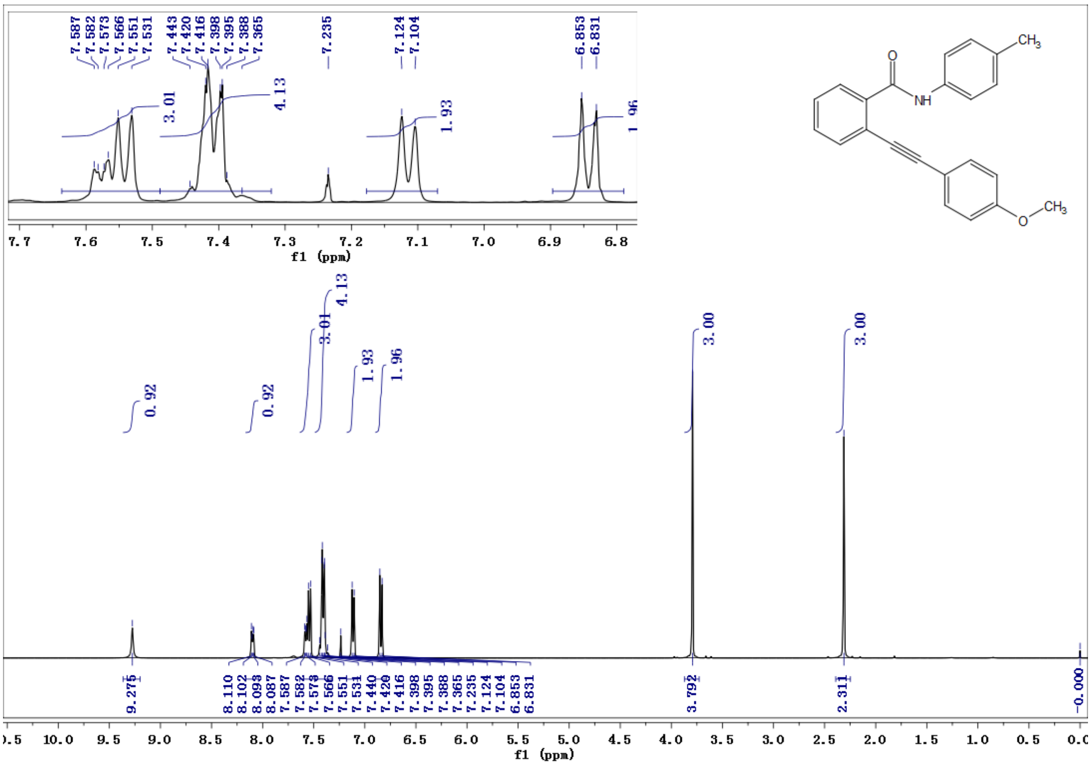

^{13}C NMR spectrum of **1afb**

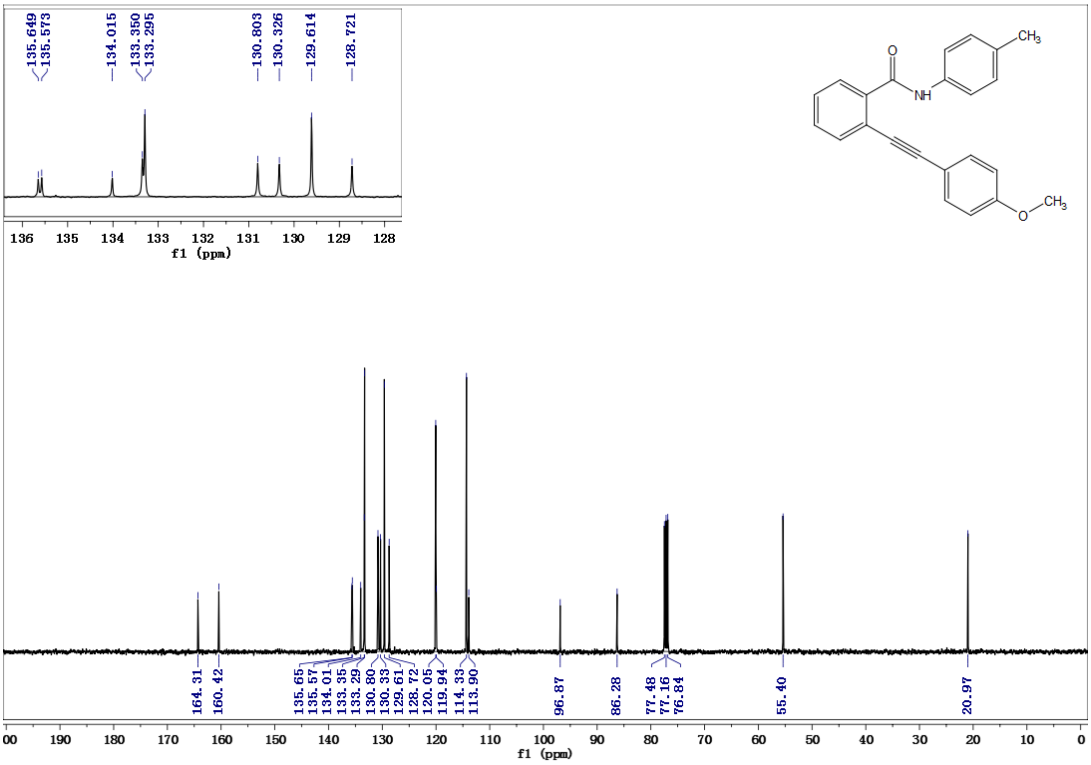

^{1}H NMR spectrum of **1agb**

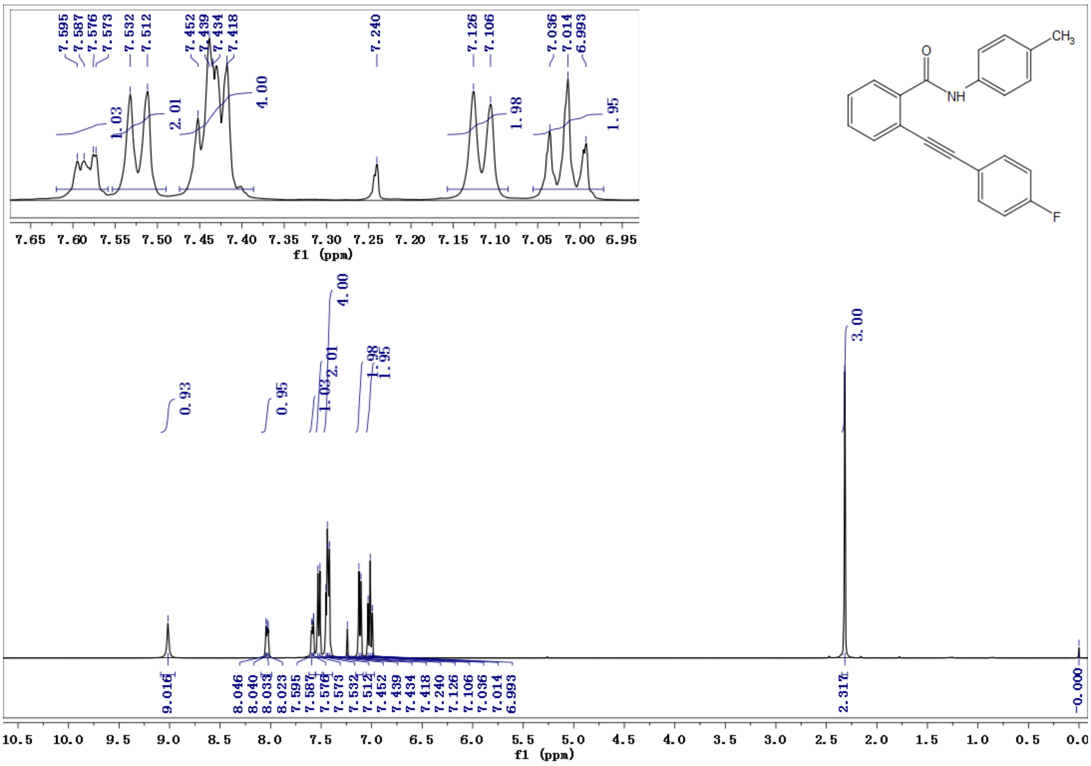

^{13}C NMR spectrum of **1agb**

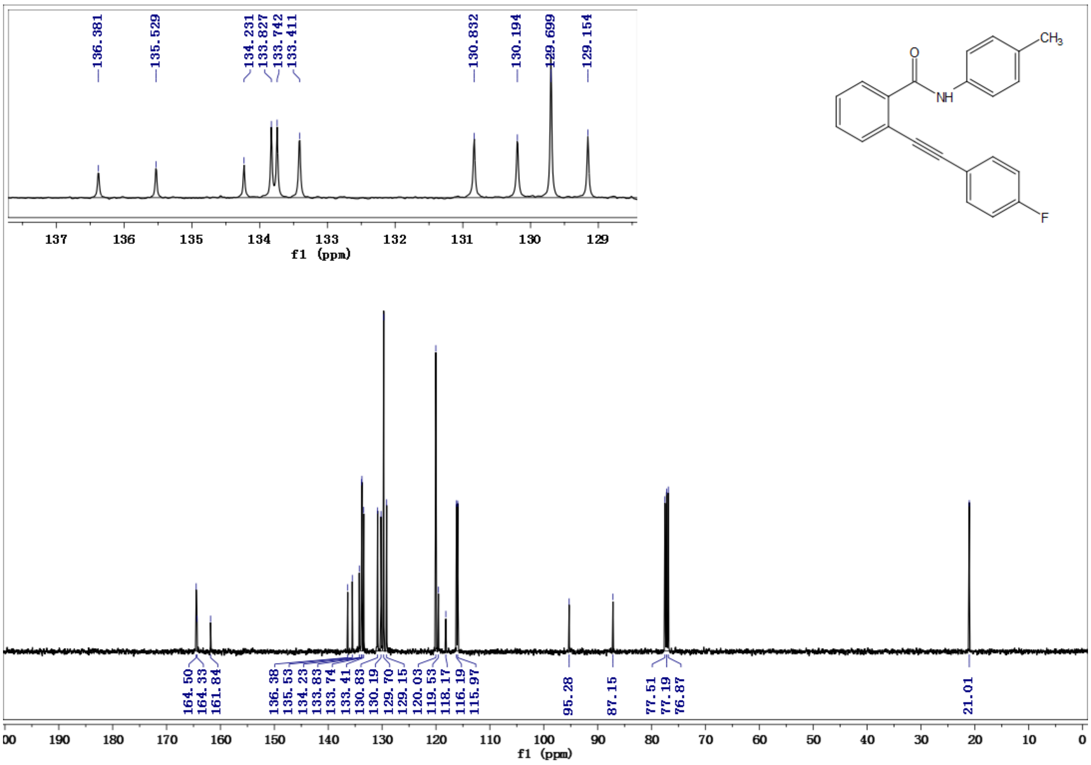

^{1}H NMR spectrum of **1ahb**

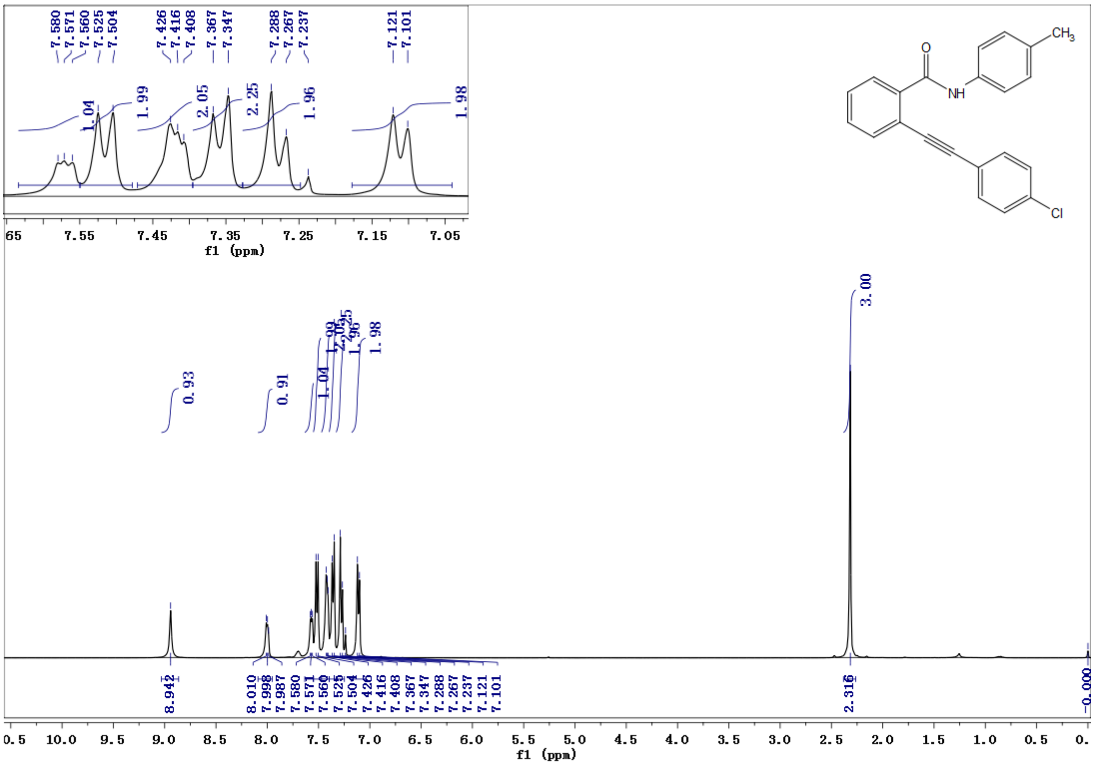

^{13}C NMR spectrum of **1ahb**

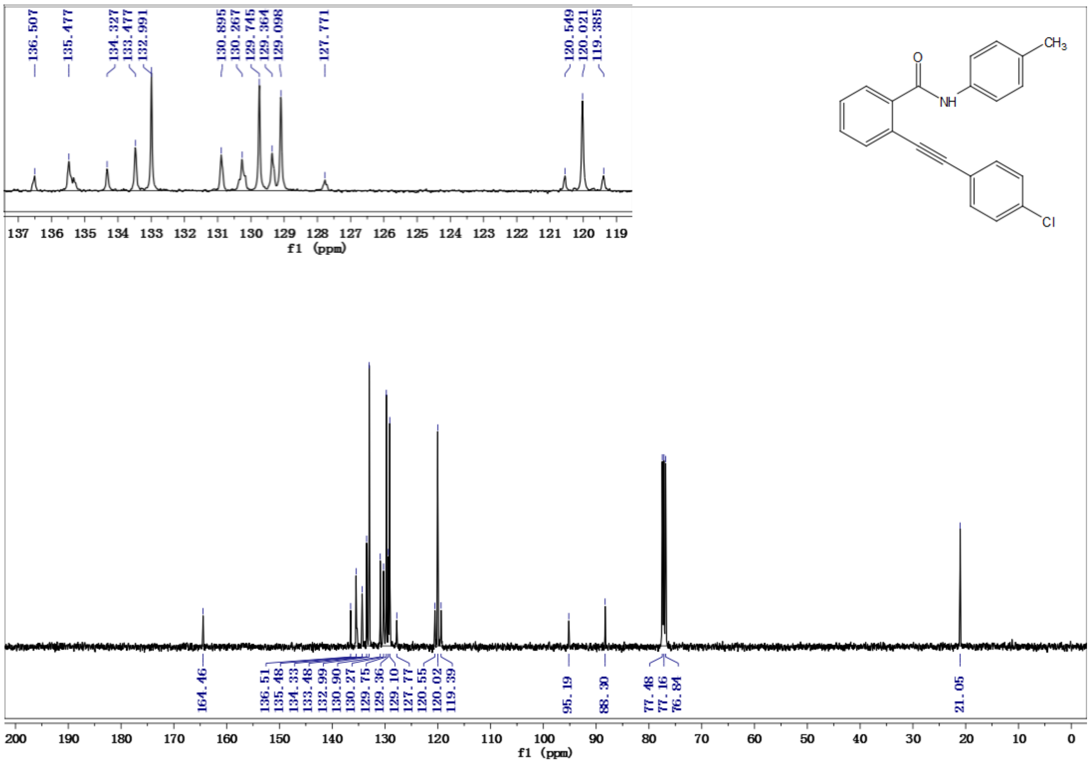

^{1}H NMR spectrum of **1aib**

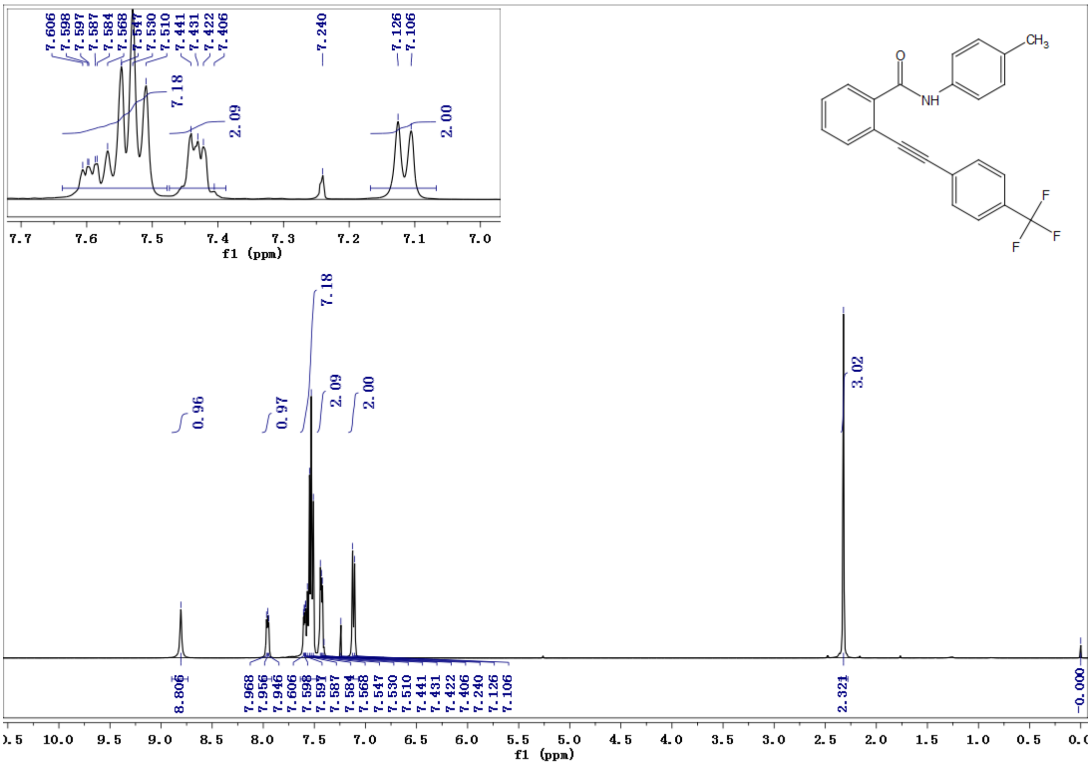

^{13}C NMR spectrum of **1aib**

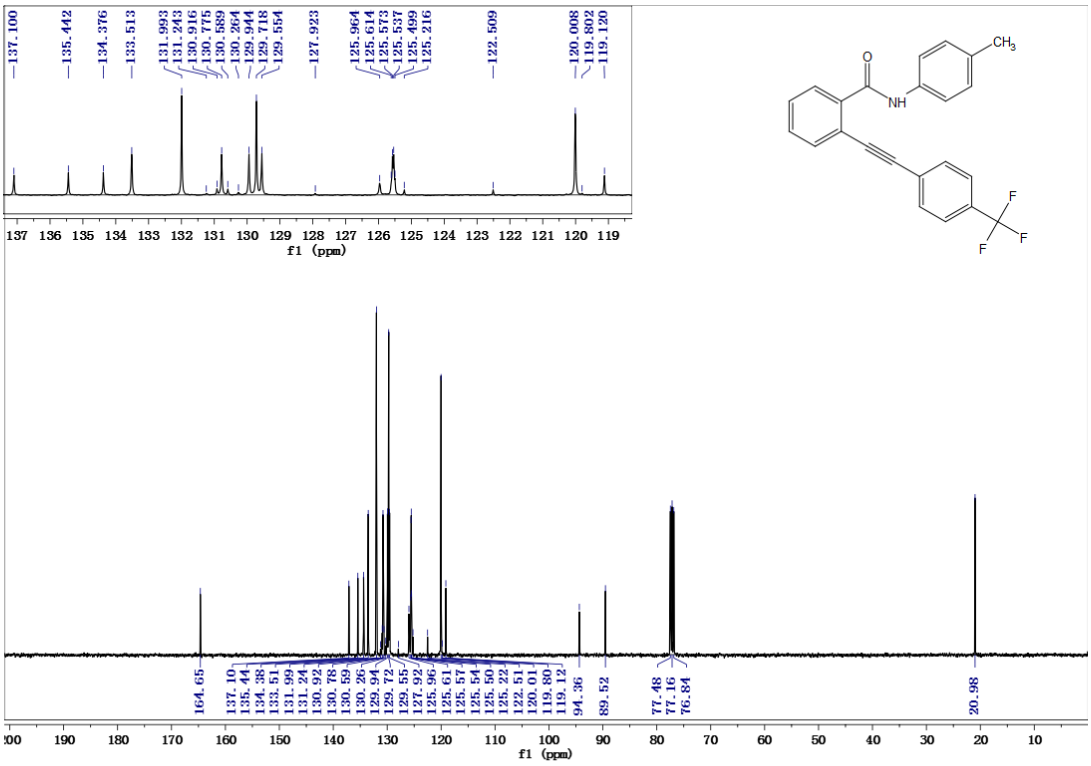

^{1}H NMR spectrum of **1ajb**

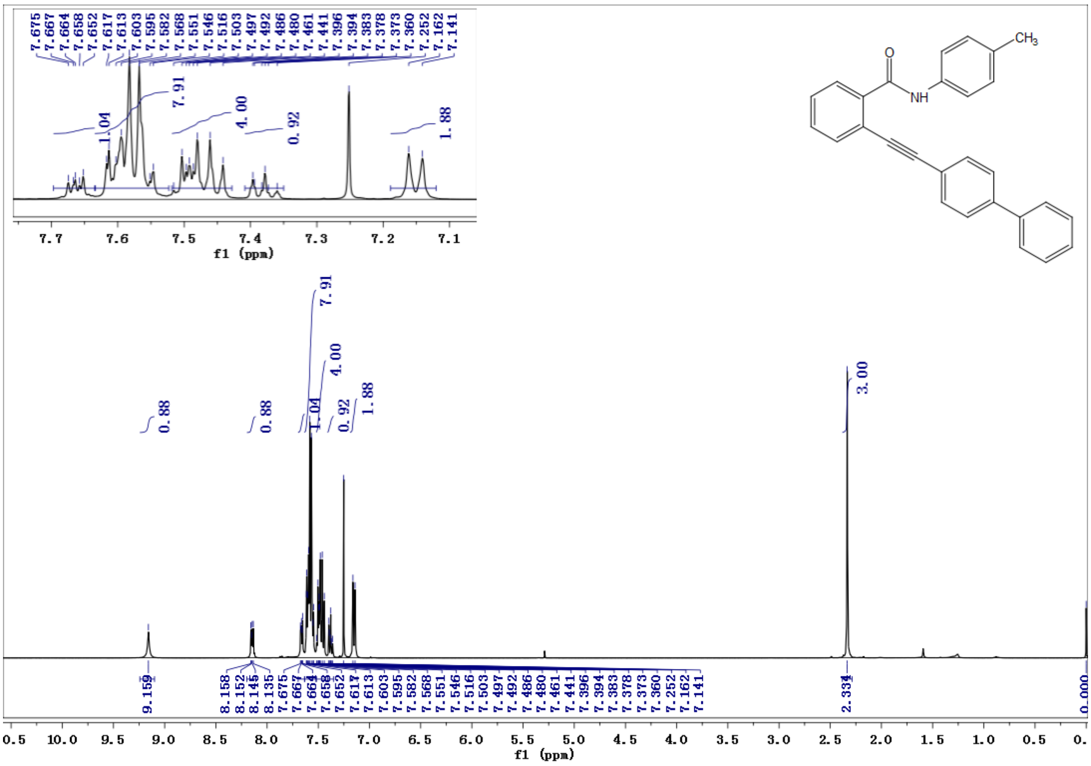

^{13}C NMR spectrum of **1ajb**

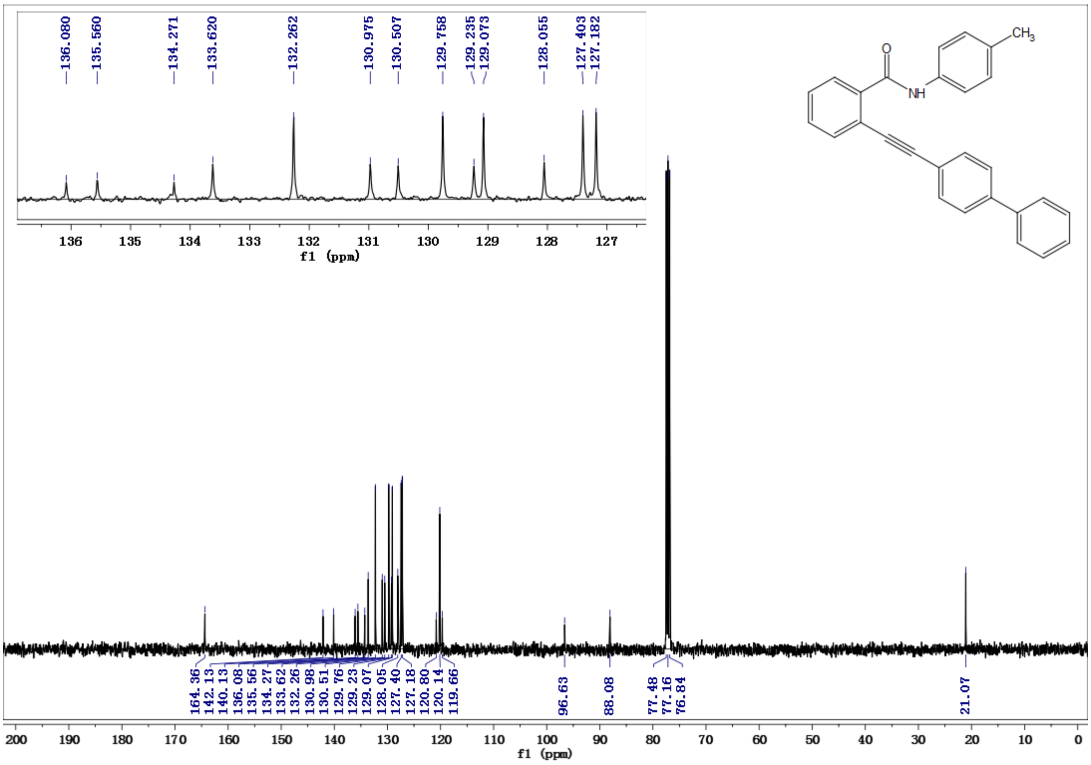

^{1}H NMR spectrum of **1aac**

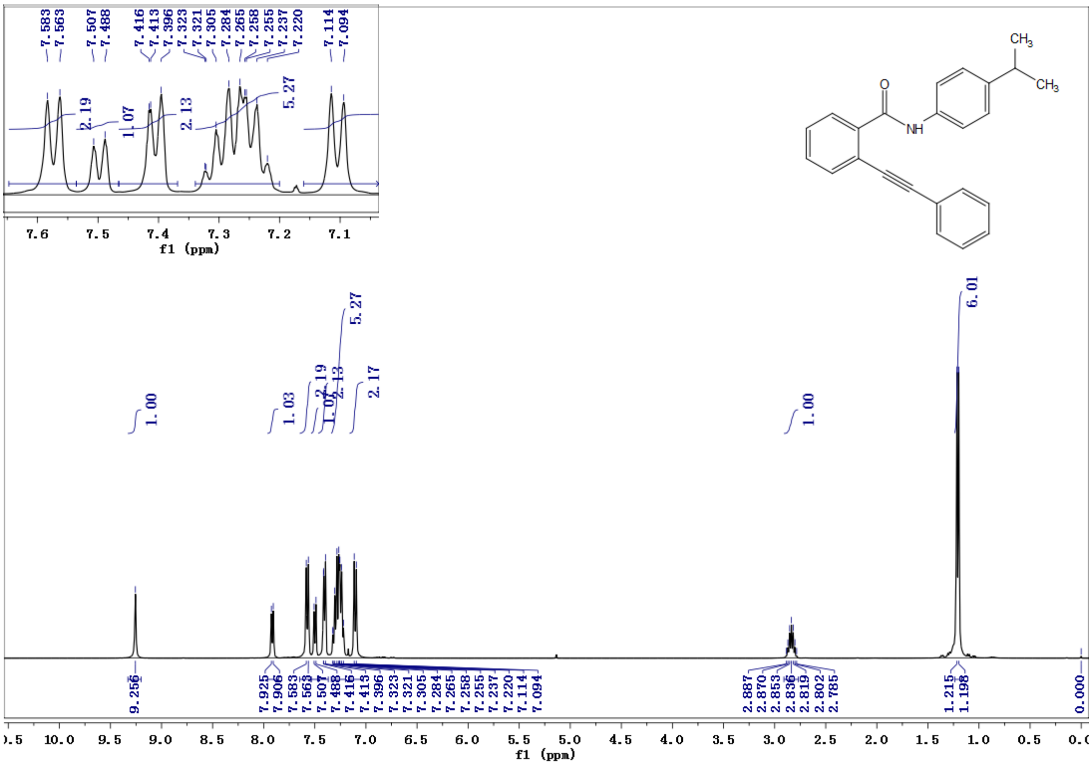

^{13}C NMR spectrum of **1aac**

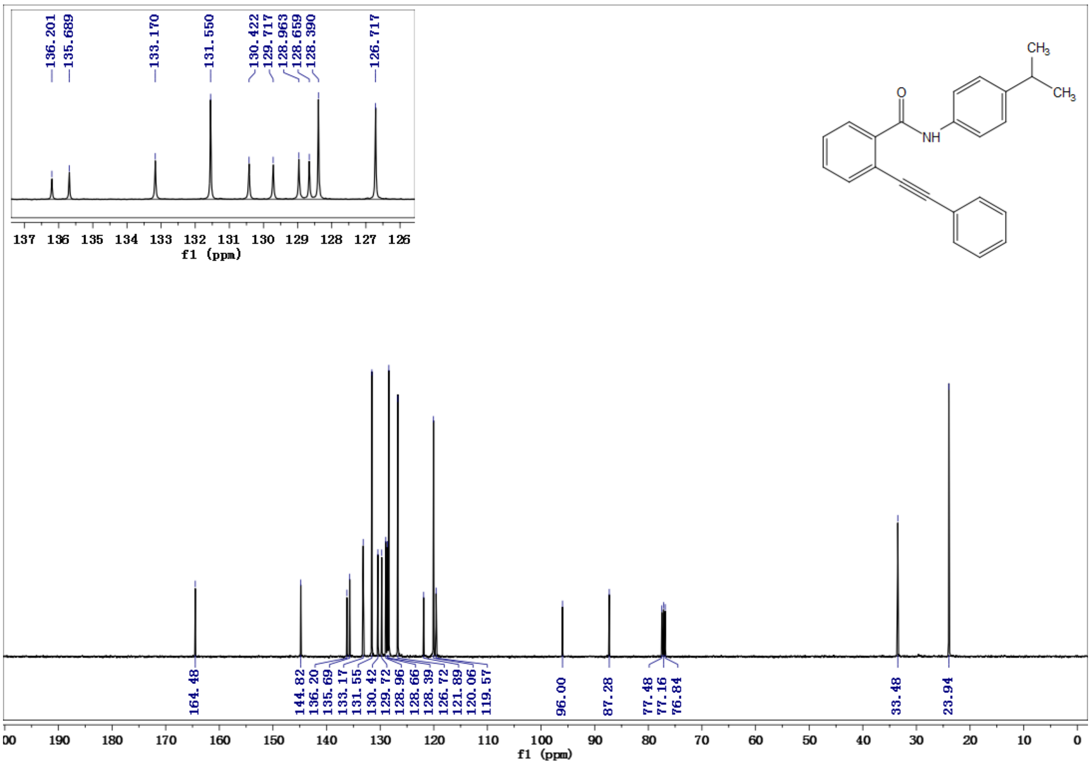

^{1}H NMR spectrum of **1aae**

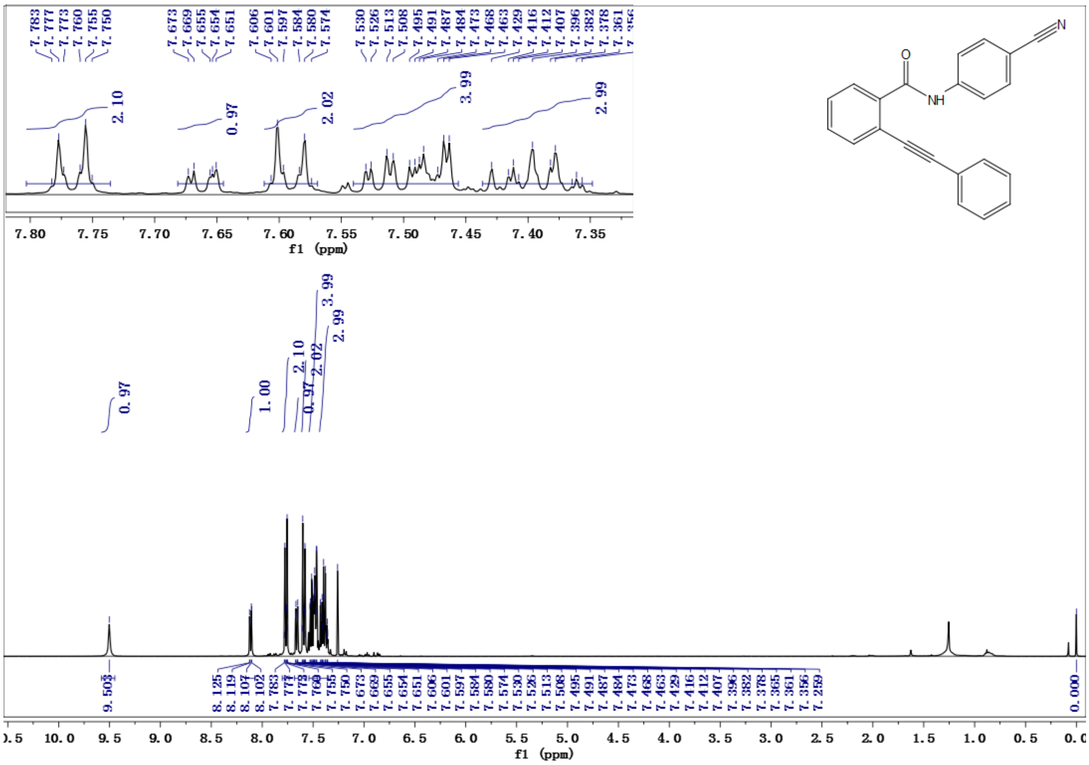

^{13}C NMR spectrum of **1aae**

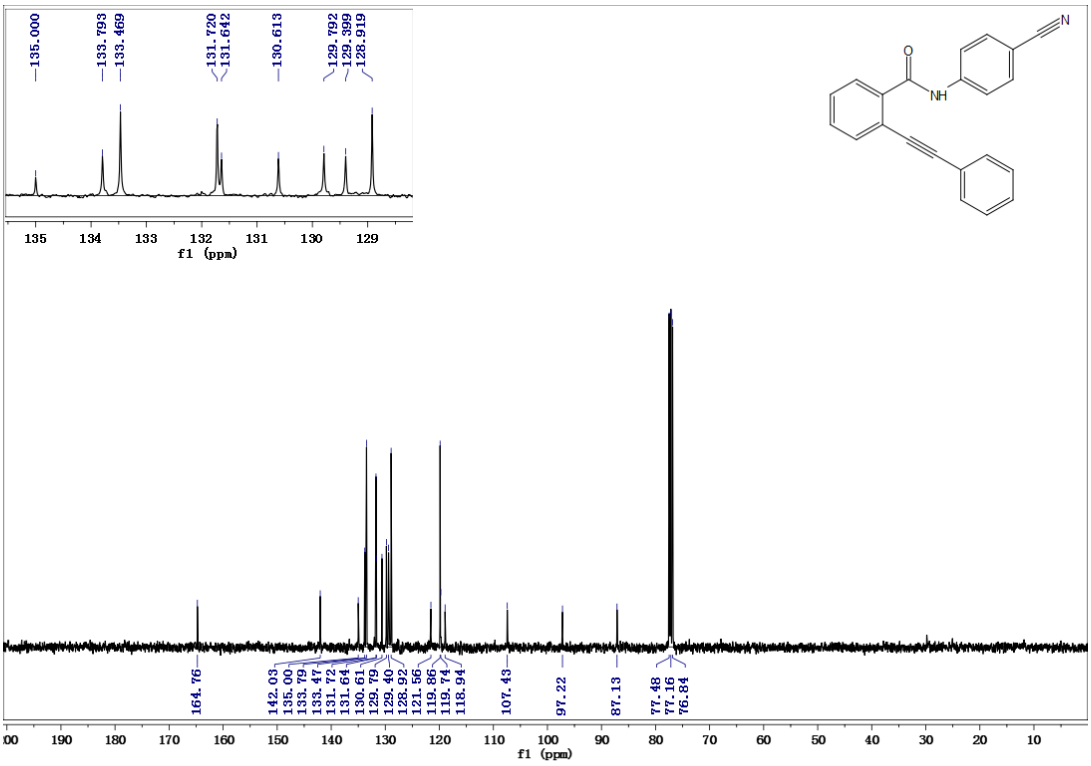

^{1}H NMR spectrum of **1afe**

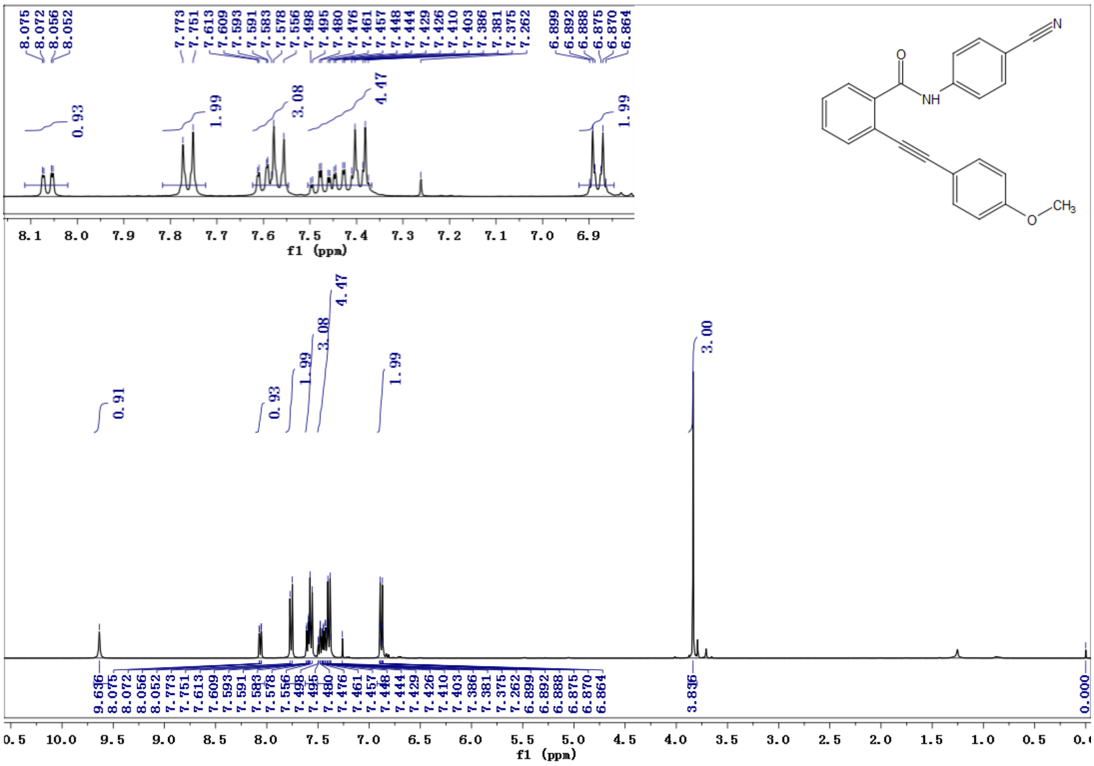

^{13}C NMR spectrum of **1afe**

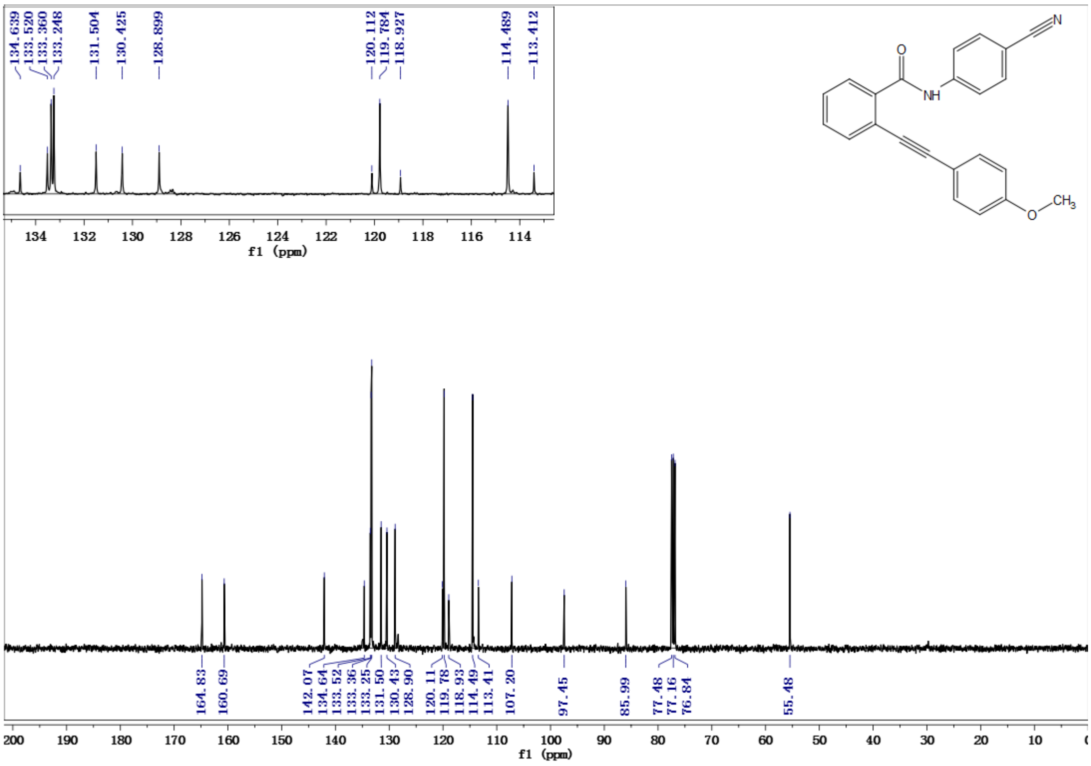

^{1}H NMR spectrum of **1aff**

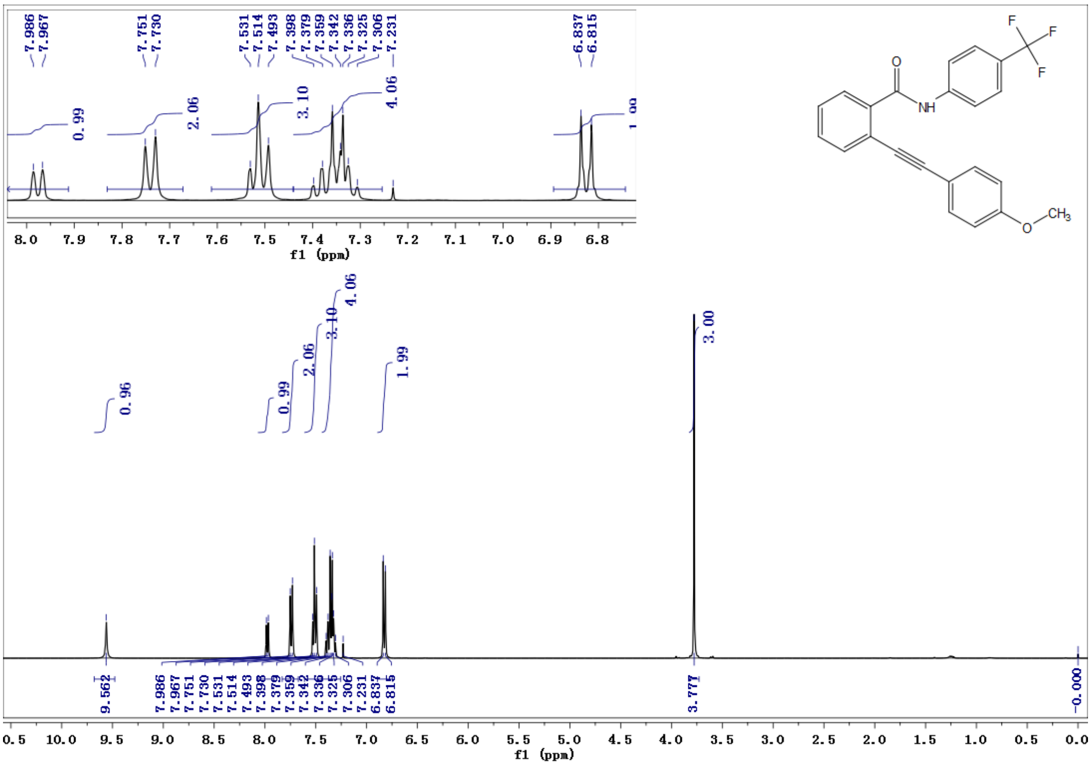

^{13}C NMR spectrum of **1aff**

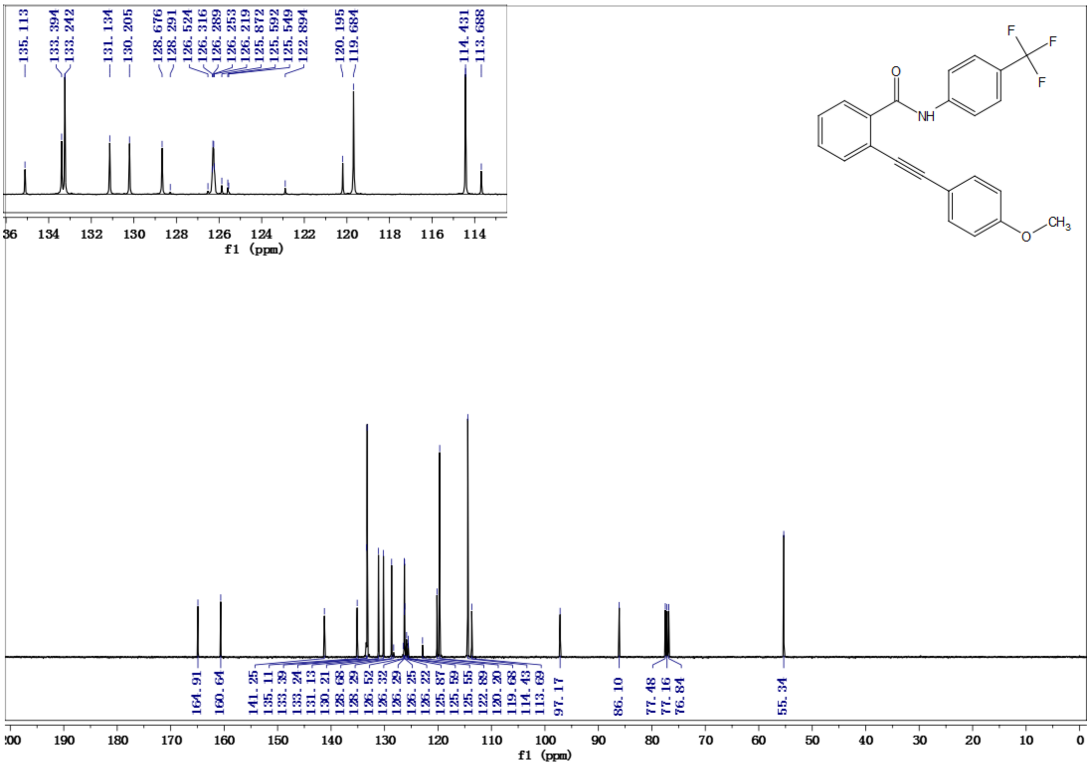

^{1}H NMR spectrum of **1baa**

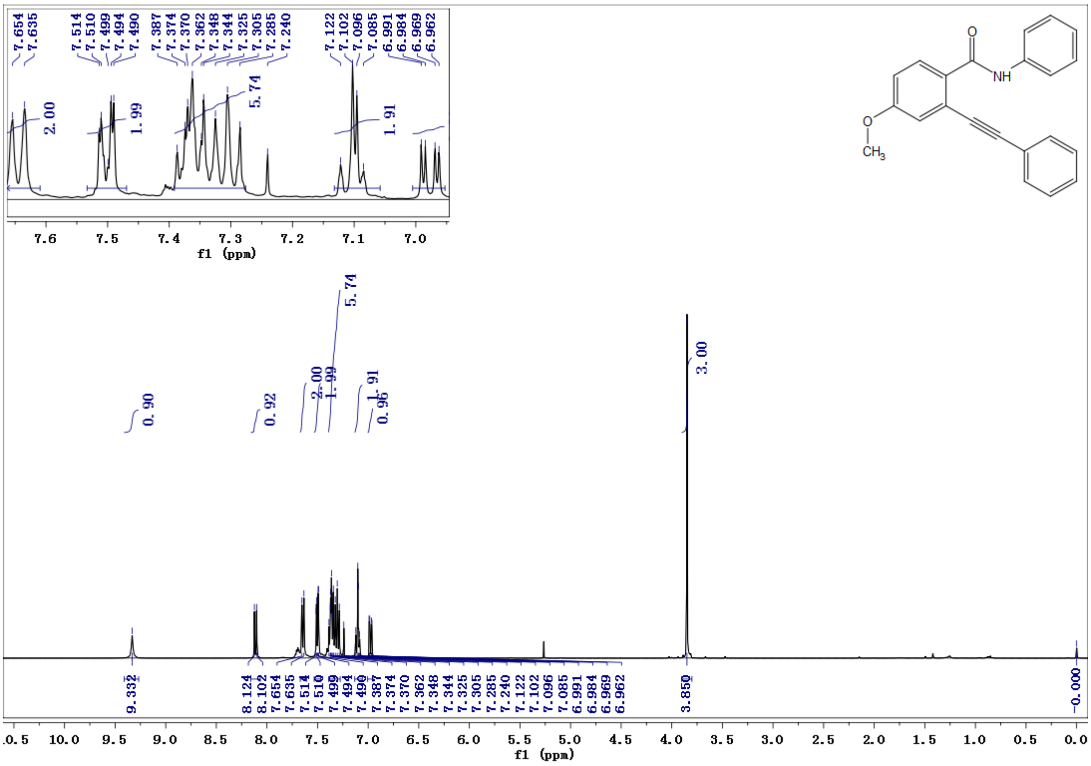

^{13}C NMR spectrum of **1baa**

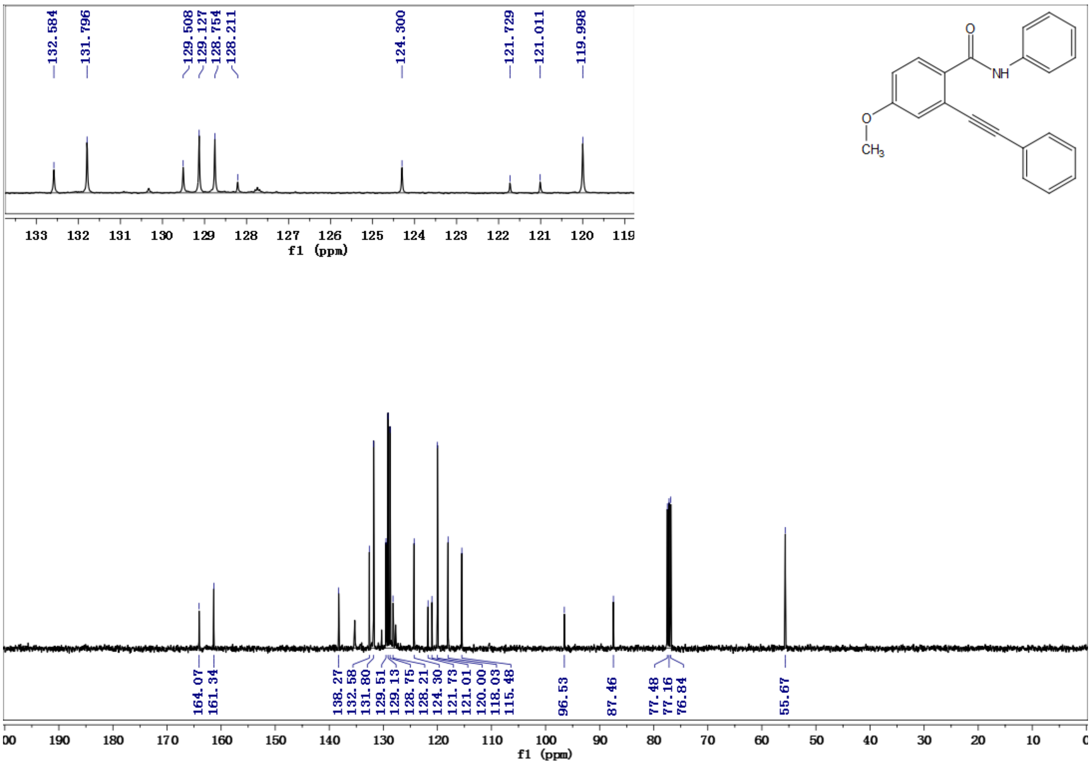

^{1}H NMR spectrum of **1caa**

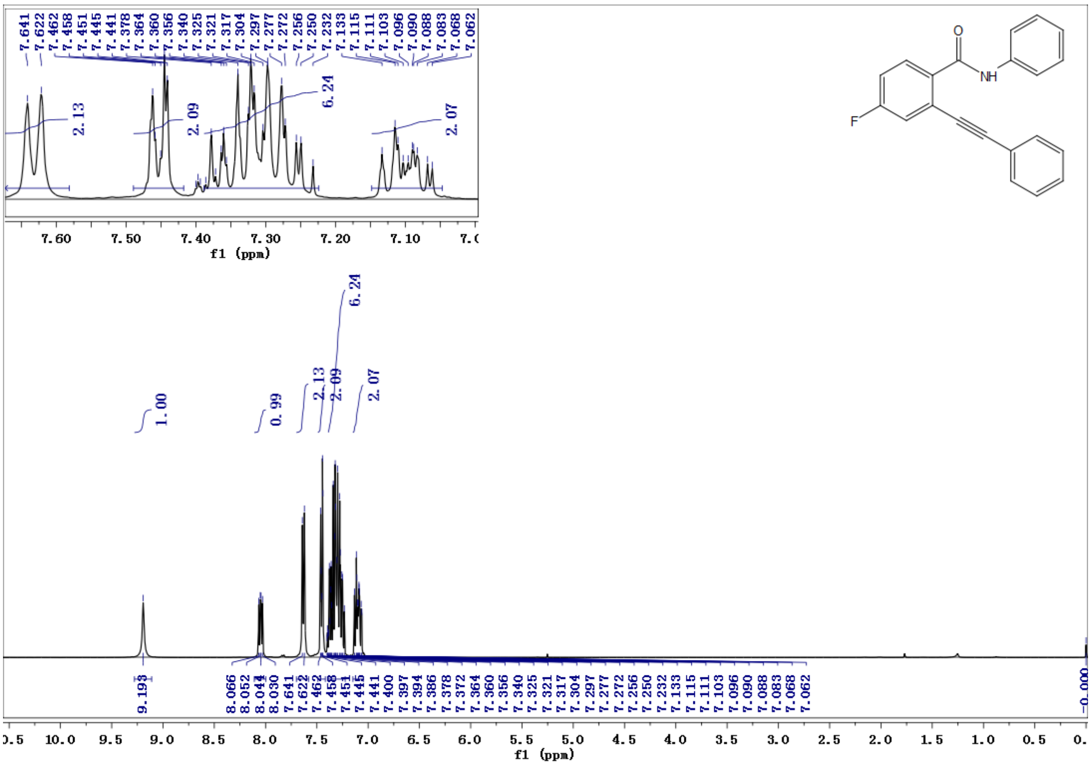

^{13}C NMR spectrum of **1caa**

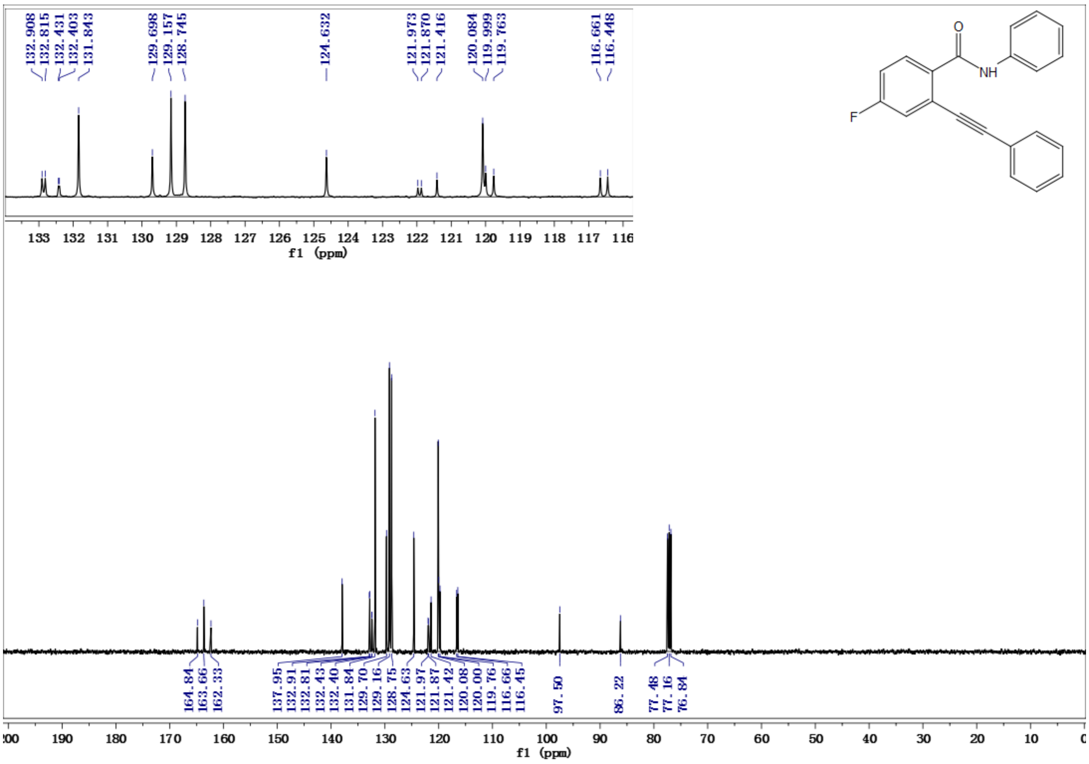

4. ^{1}H and ^{13}C NMR charts of the products

^{1}H NMR spectrum of **2aaa**

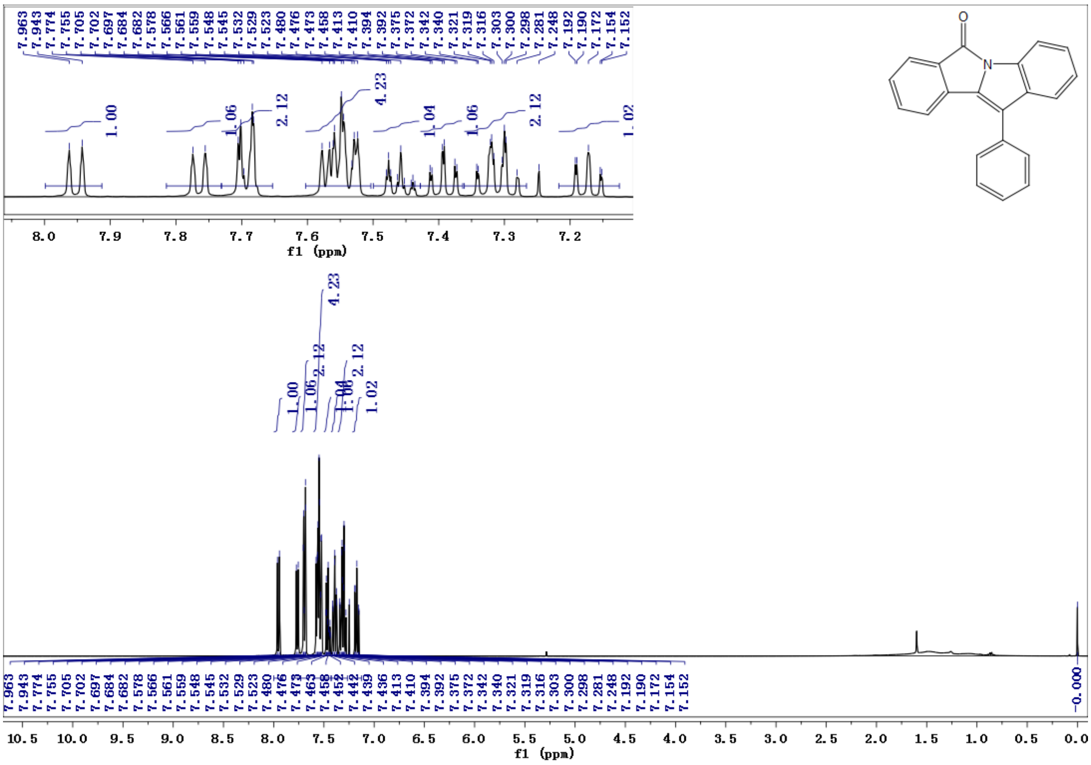

^{13}C NMR spectrum of **2aaa**

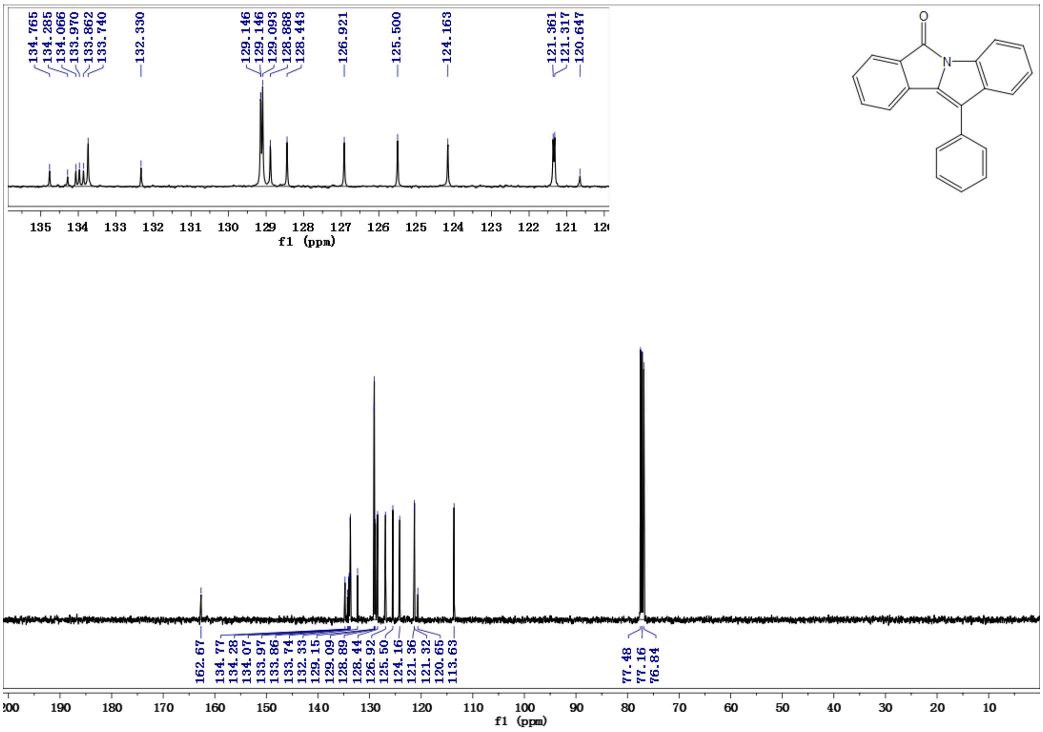

^{1}H NMR spectrum of **2aba**

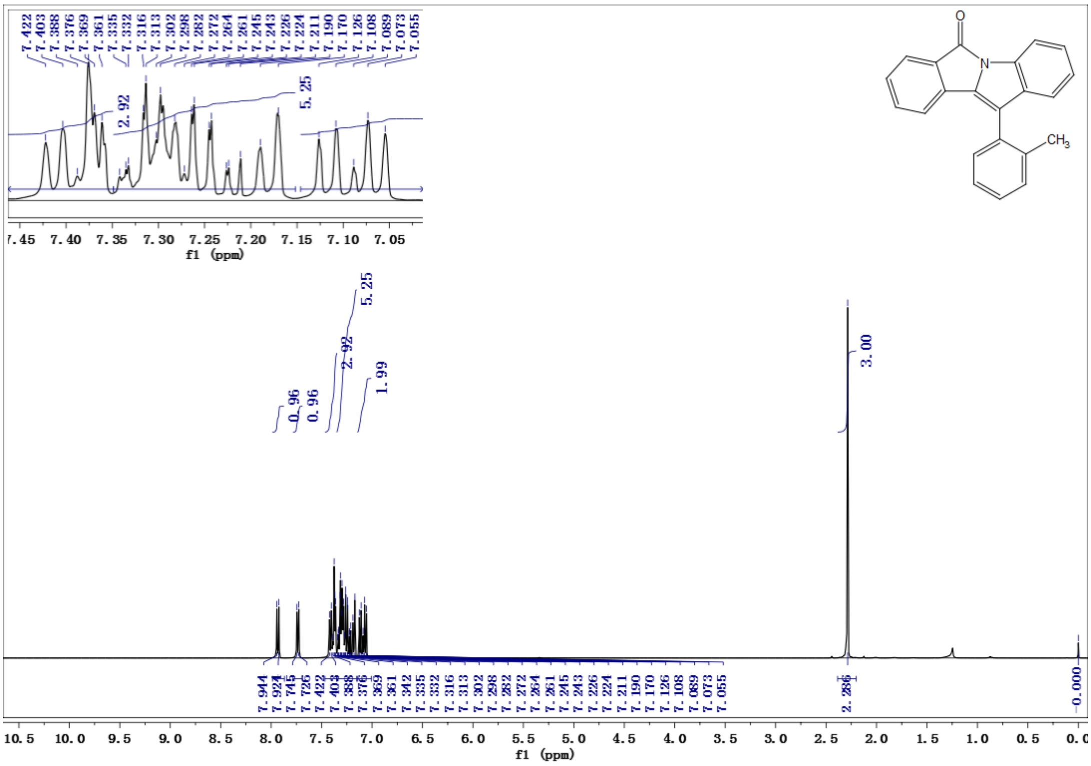

^{13}C NMR spectrum of **2aba**

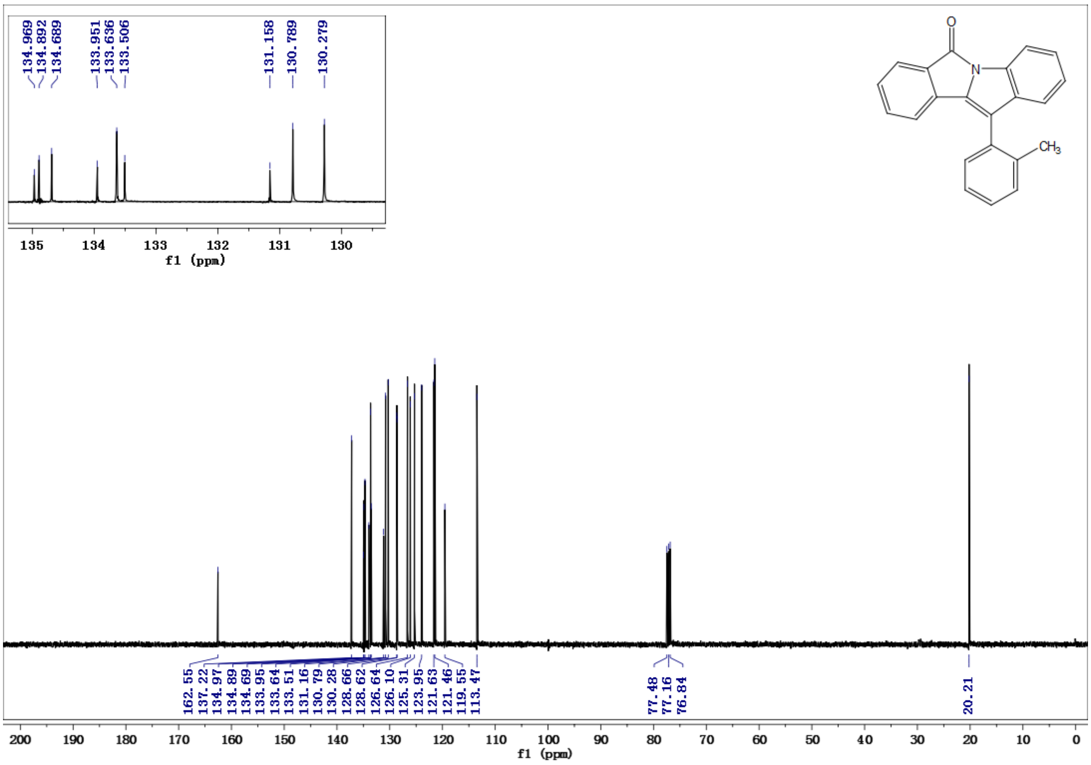

^{1}H NMR spectrum of **2aca**

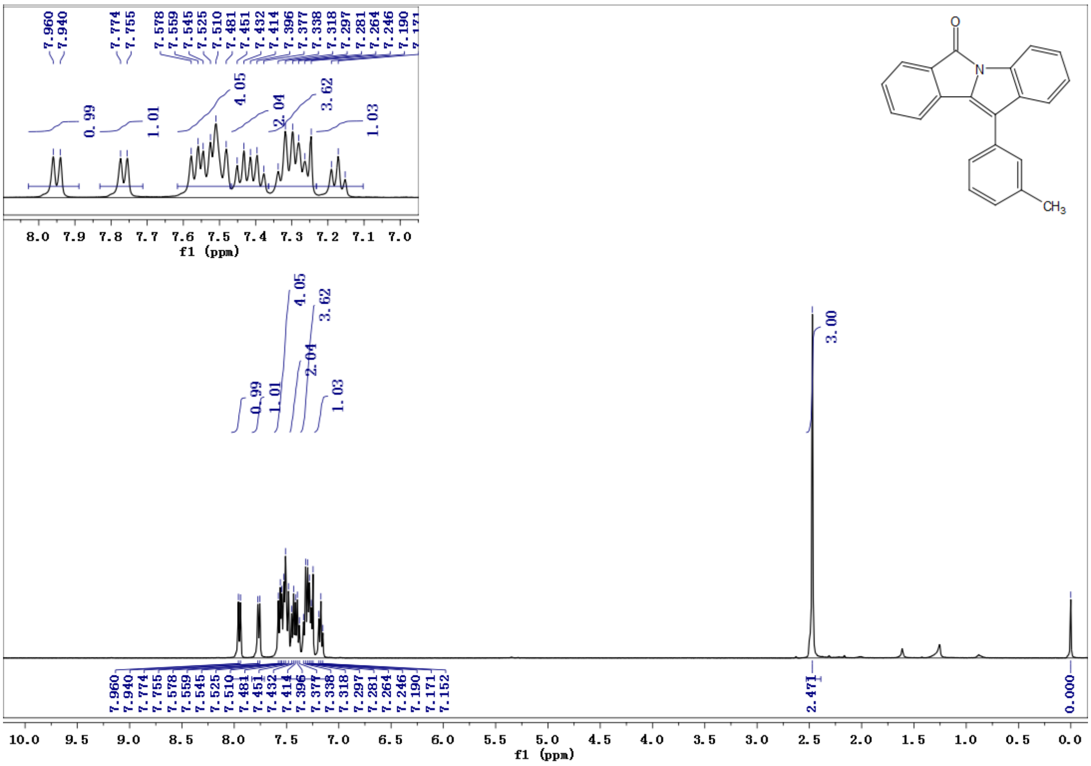

^{13}C NMR spectrum of **2aca**

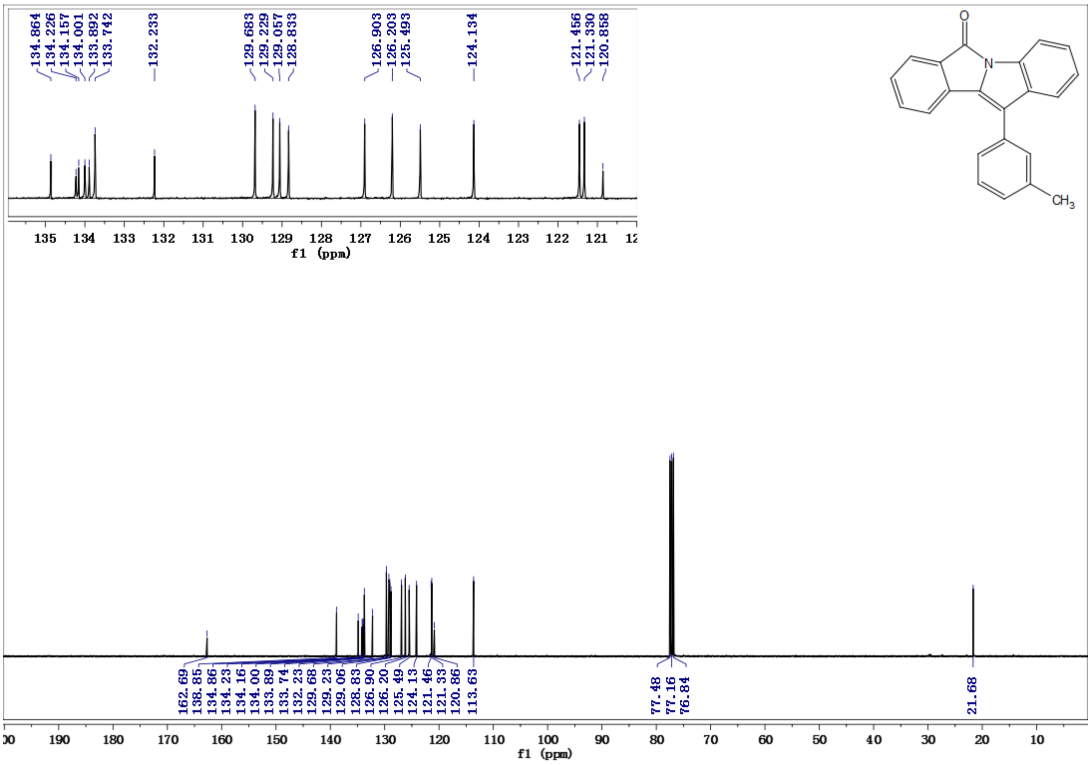

^{1}H NMR spectrum of **2ada**

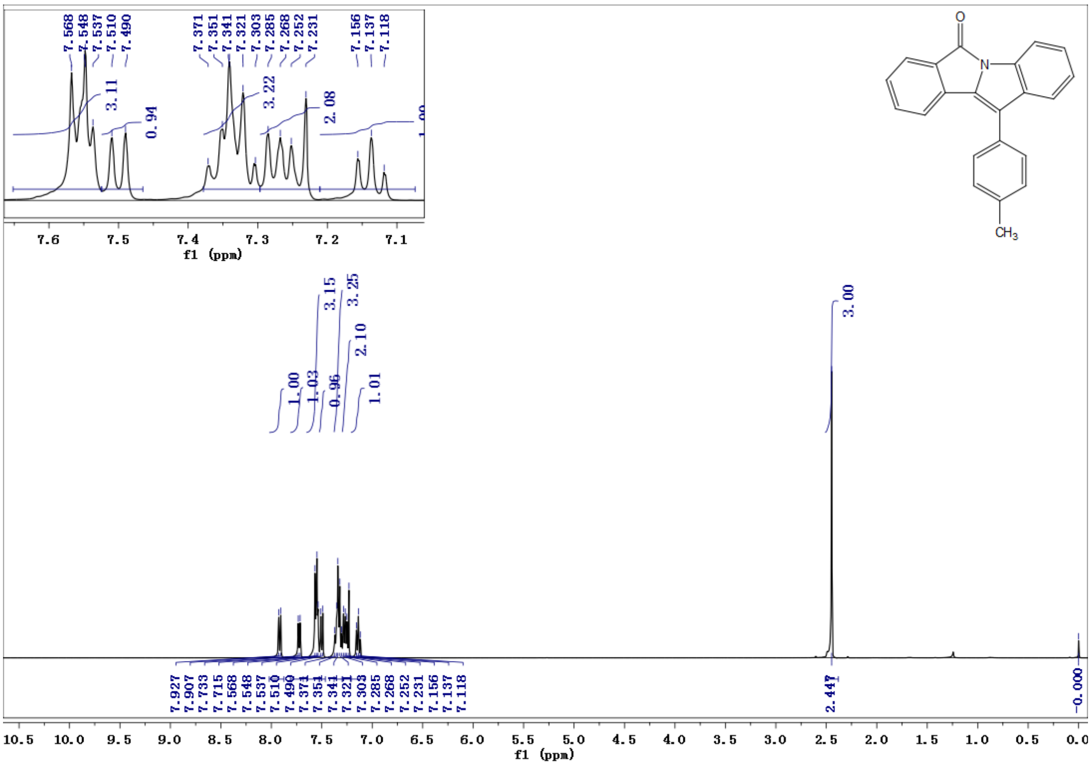

^{13}C NMR spectrum of **2ada**

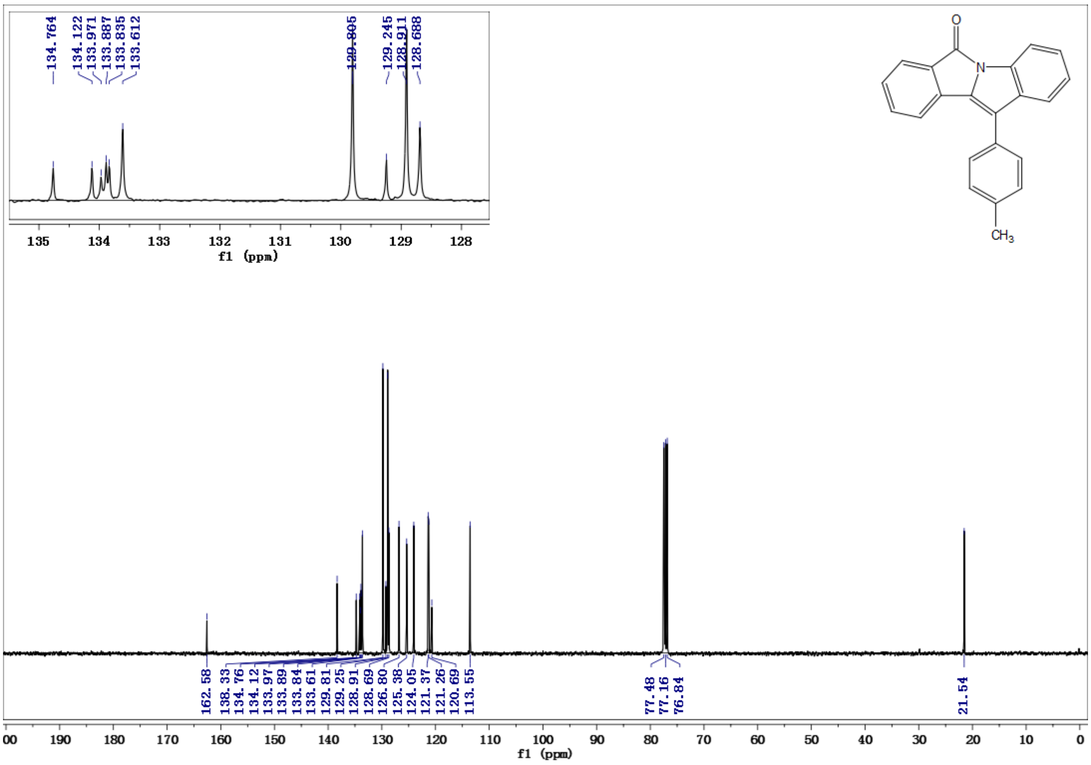

^{1}H NMR spectrum of **2aea**

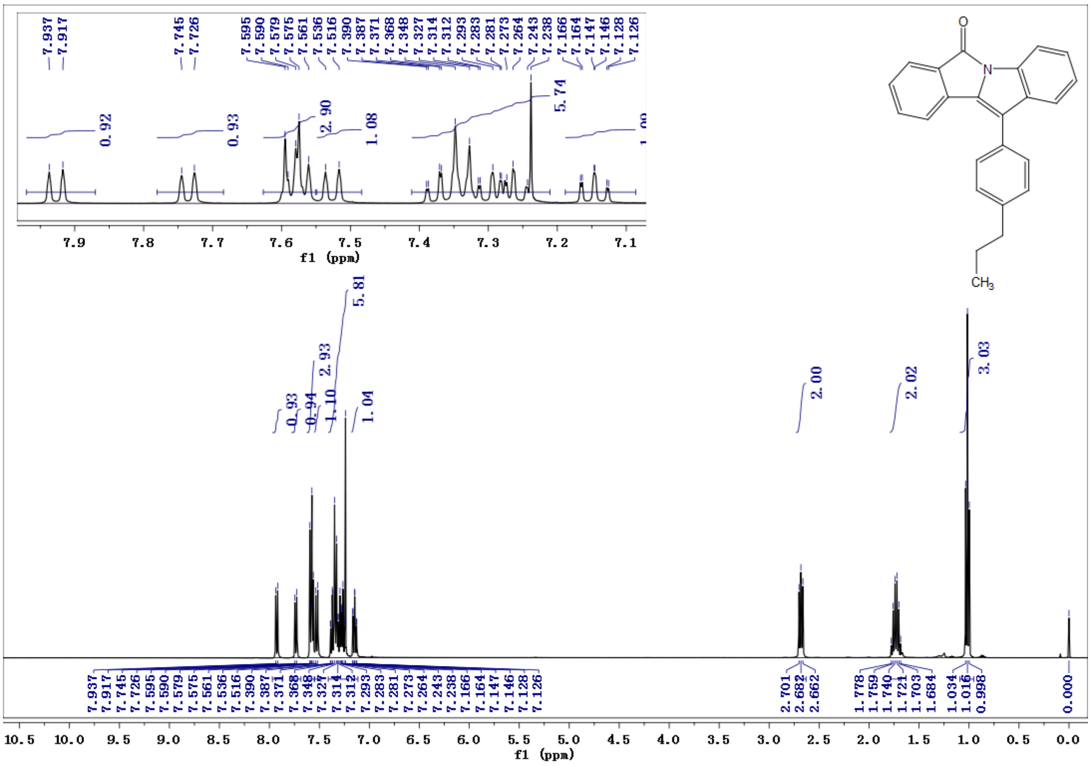

^{13}C NMR spectrum of **2aea**

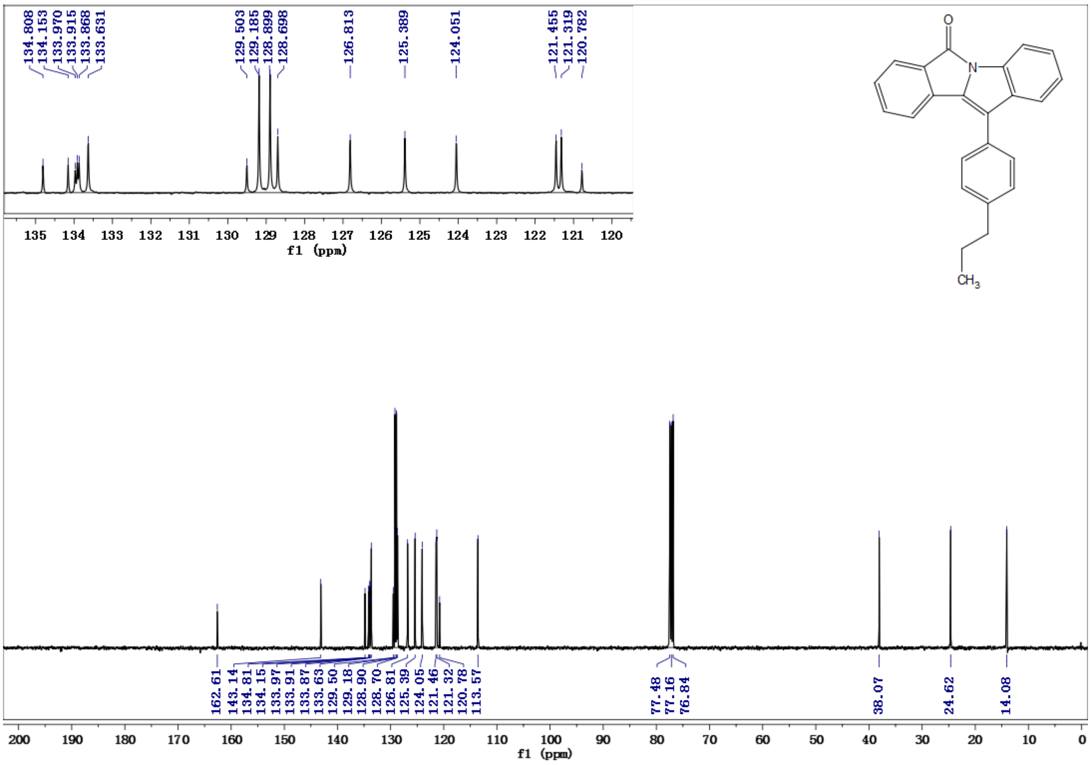

^{1}H NMR spectrum of **2afa**

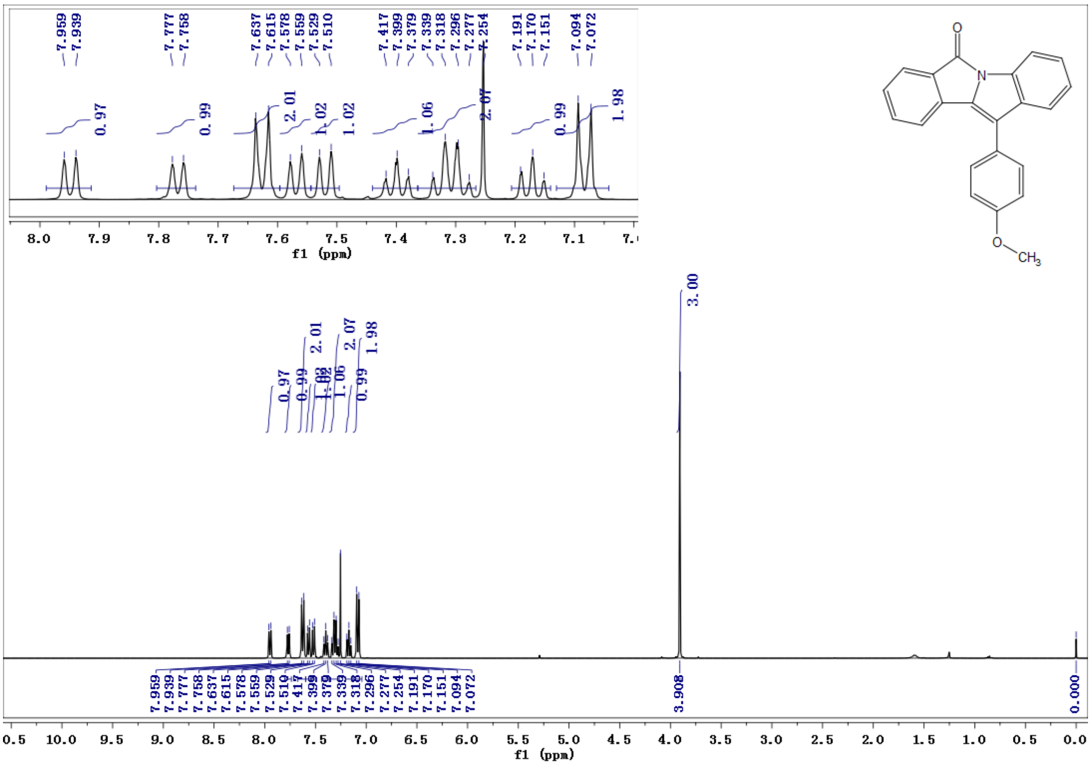

^{13}C NMR spectrum of **2afa**

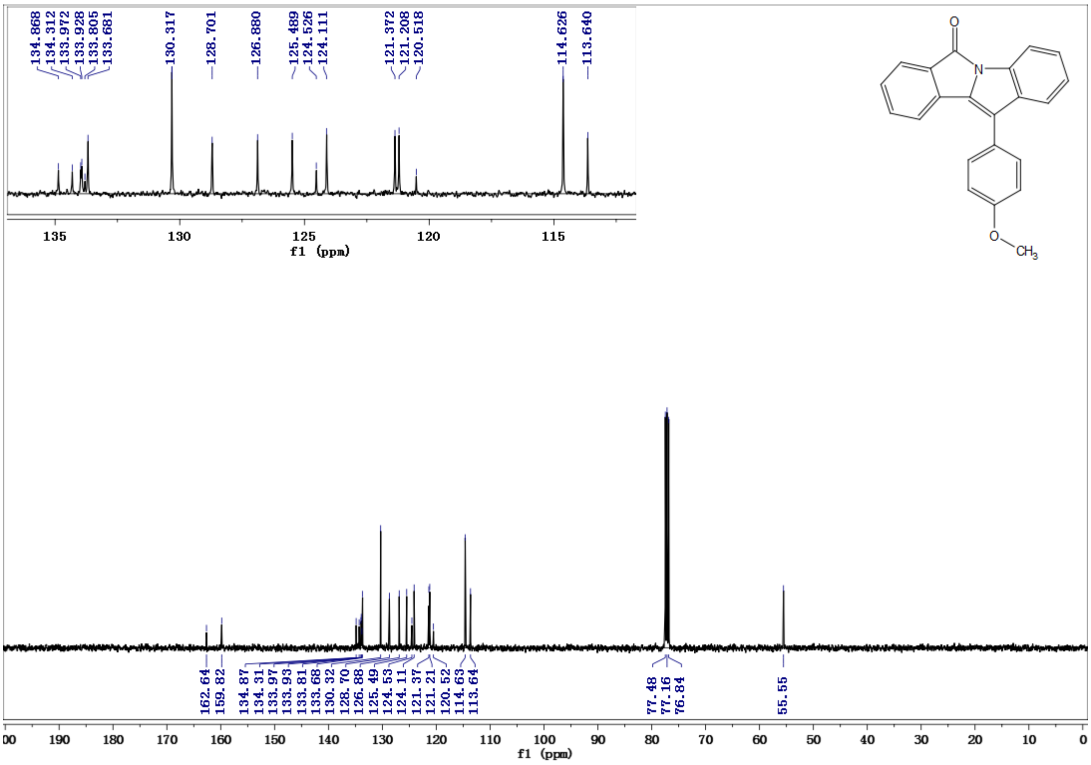

^{1}H NMR spectrum of **2aga**

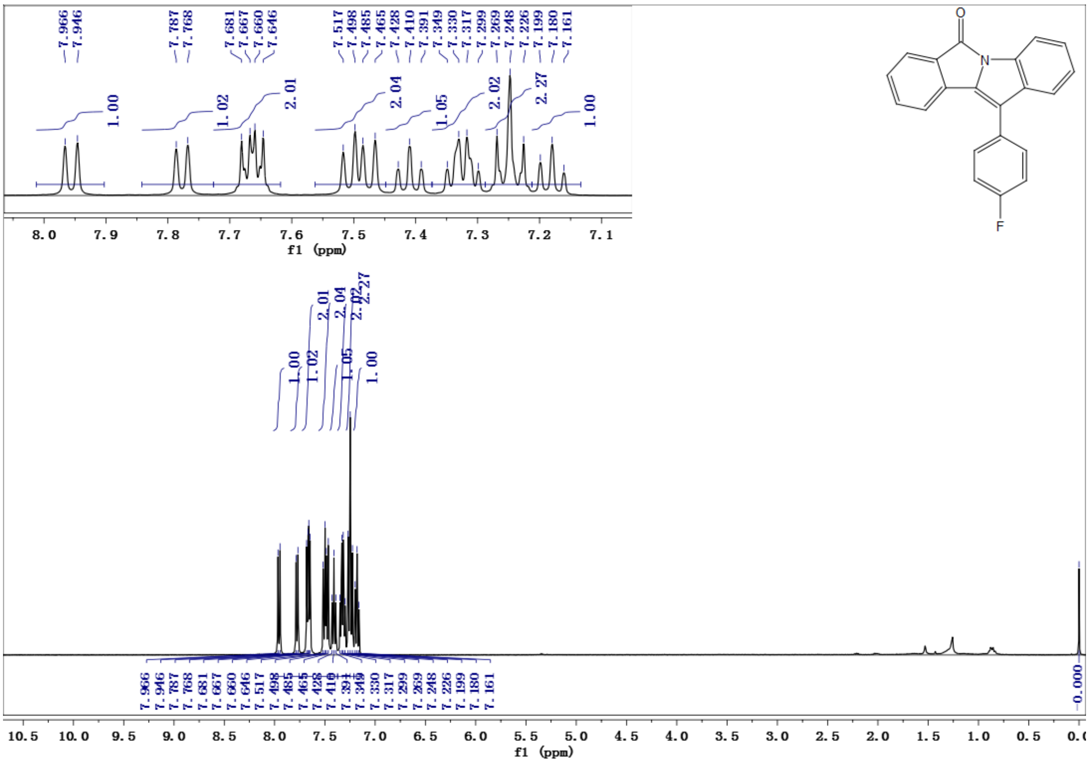

^{13}C NMR spectrum of **2aga**

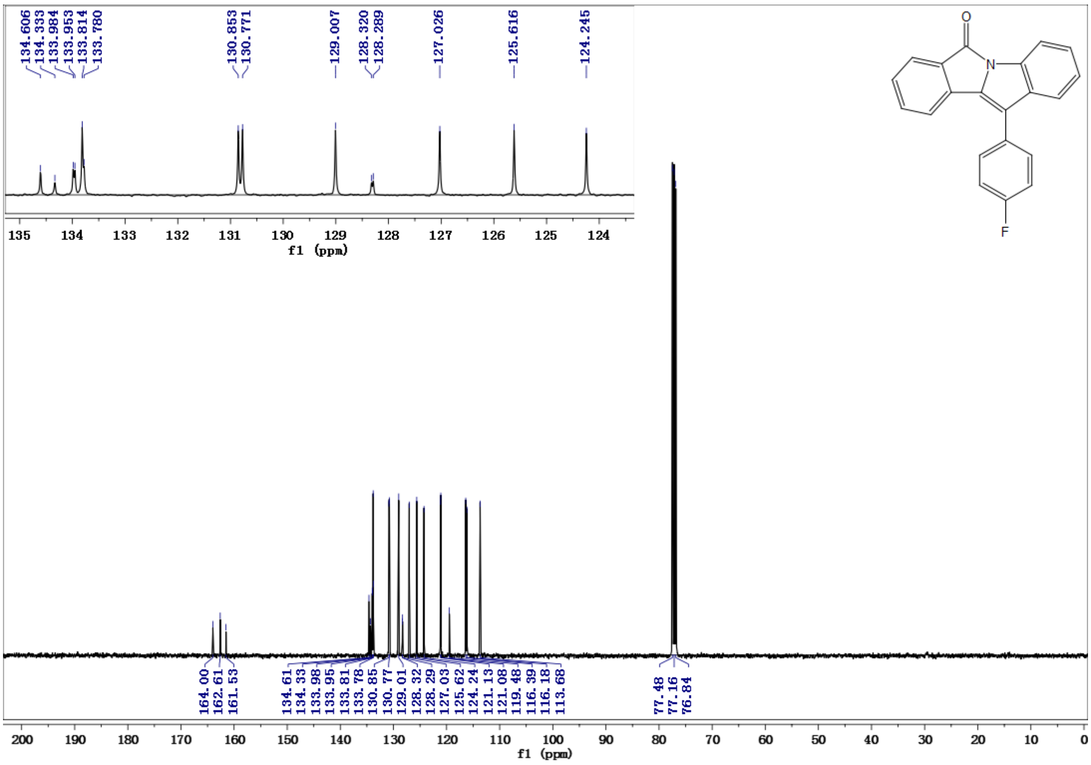

^{19}F NMR spectrum of **2aga**

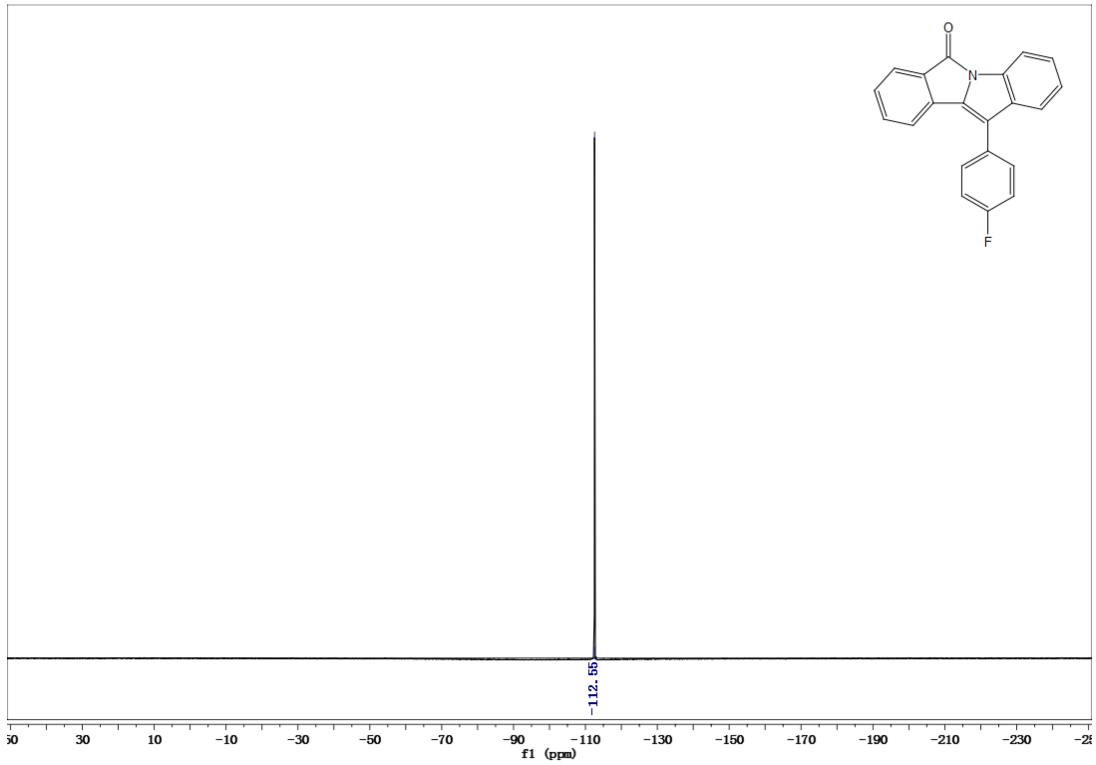

^{1}H NMR spectrum of **2aha**

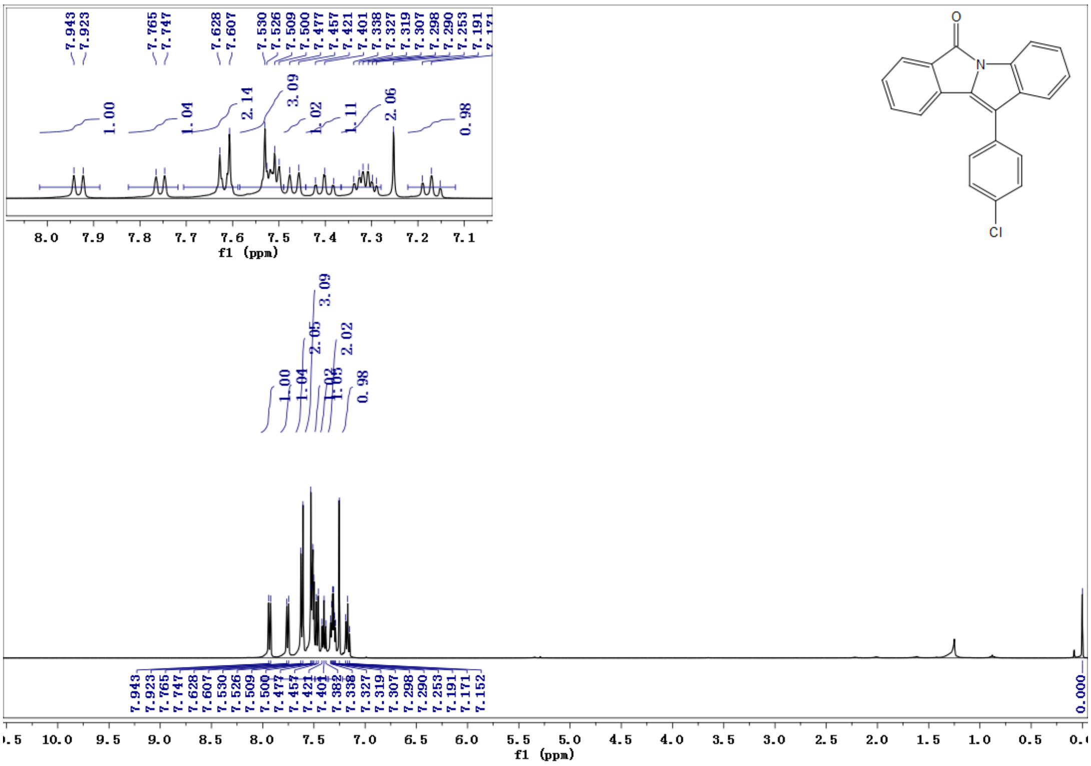

^{13}C NMR spectrum of **2aha**

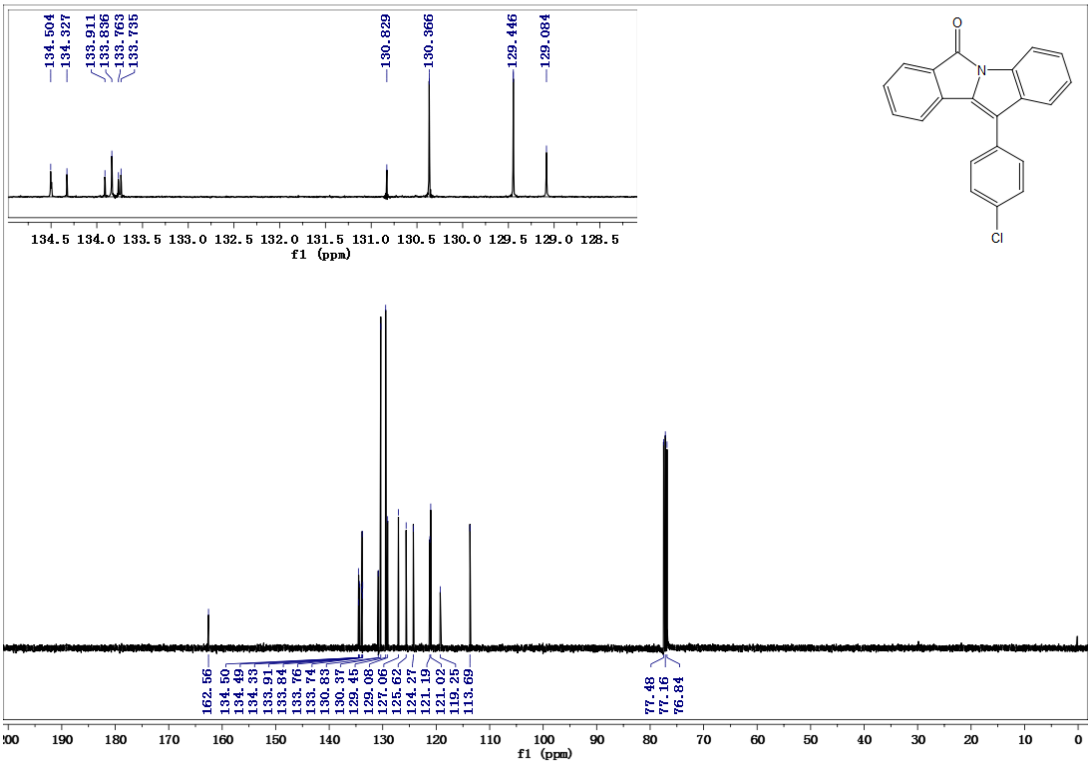

^{1}H NMR spectrum of **2aia**

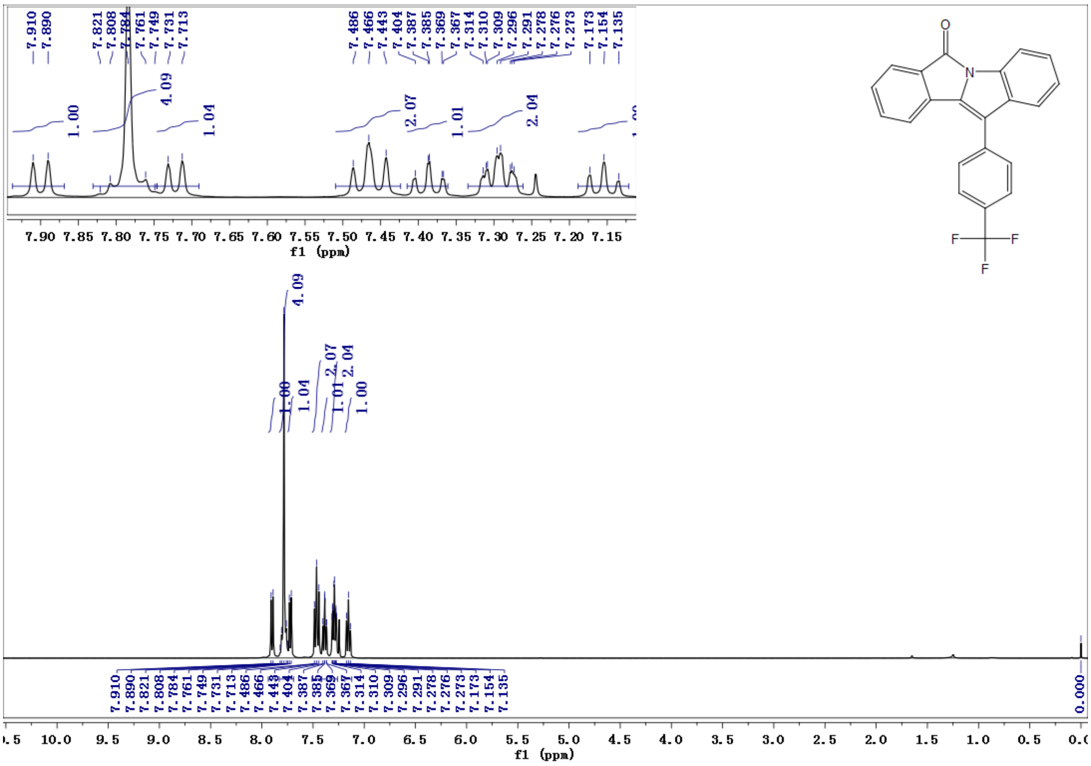

^{13}C NMR spectrum of **2aia**

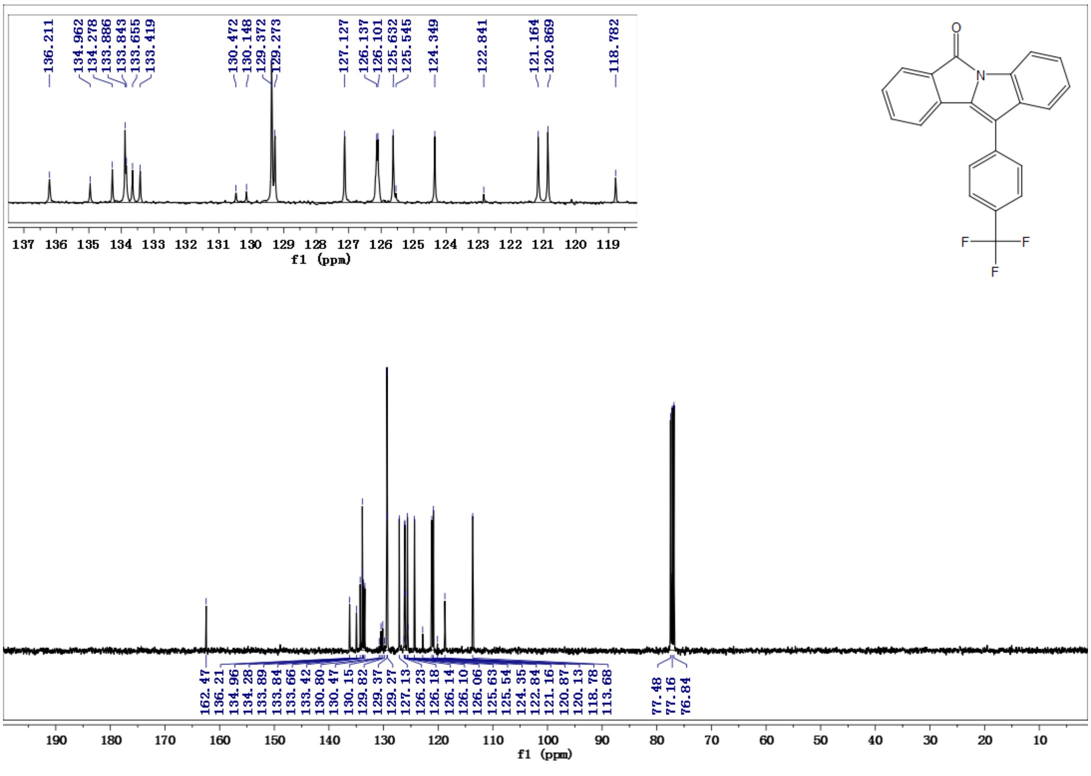

^{19}F NMR spectrum of **2aia**

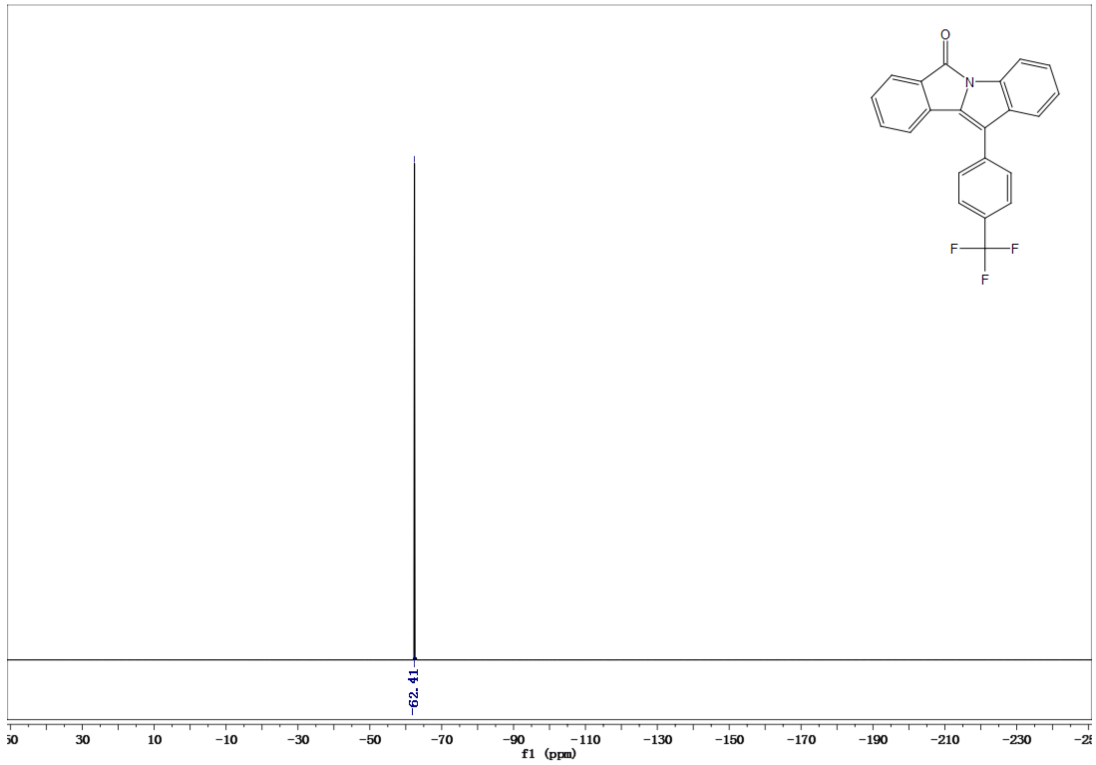

^{1}H NMR spectrum of **2aja**

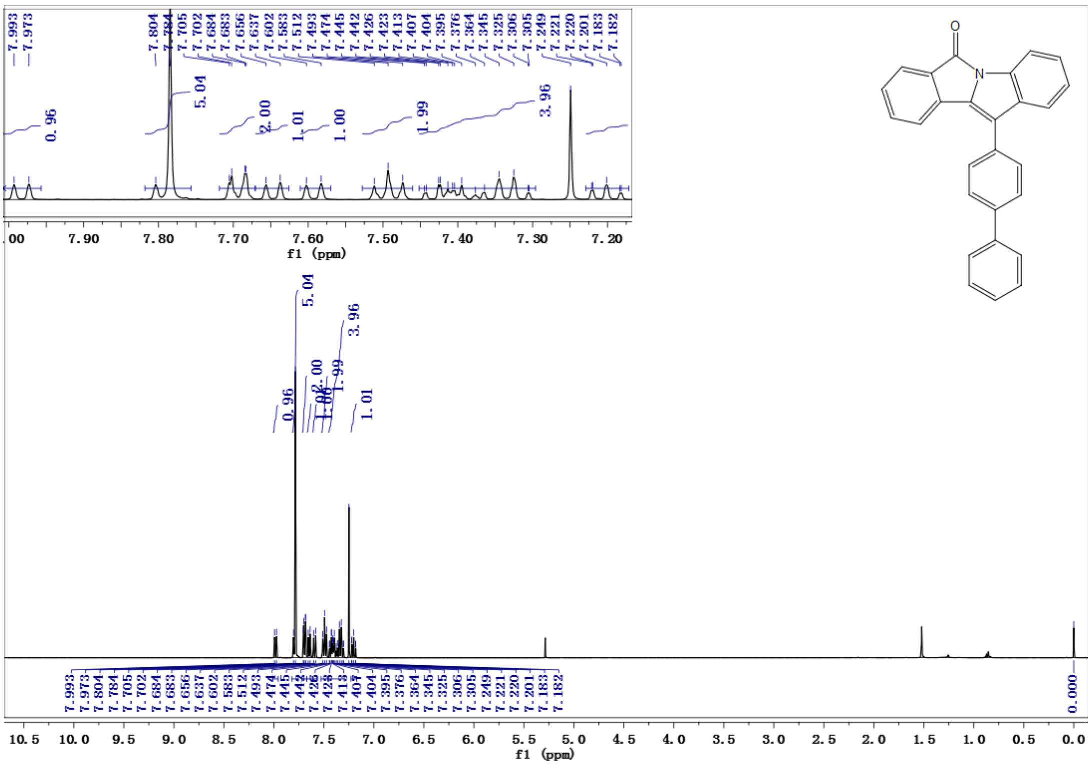

^{13}C NMR spectrum of **2aja**

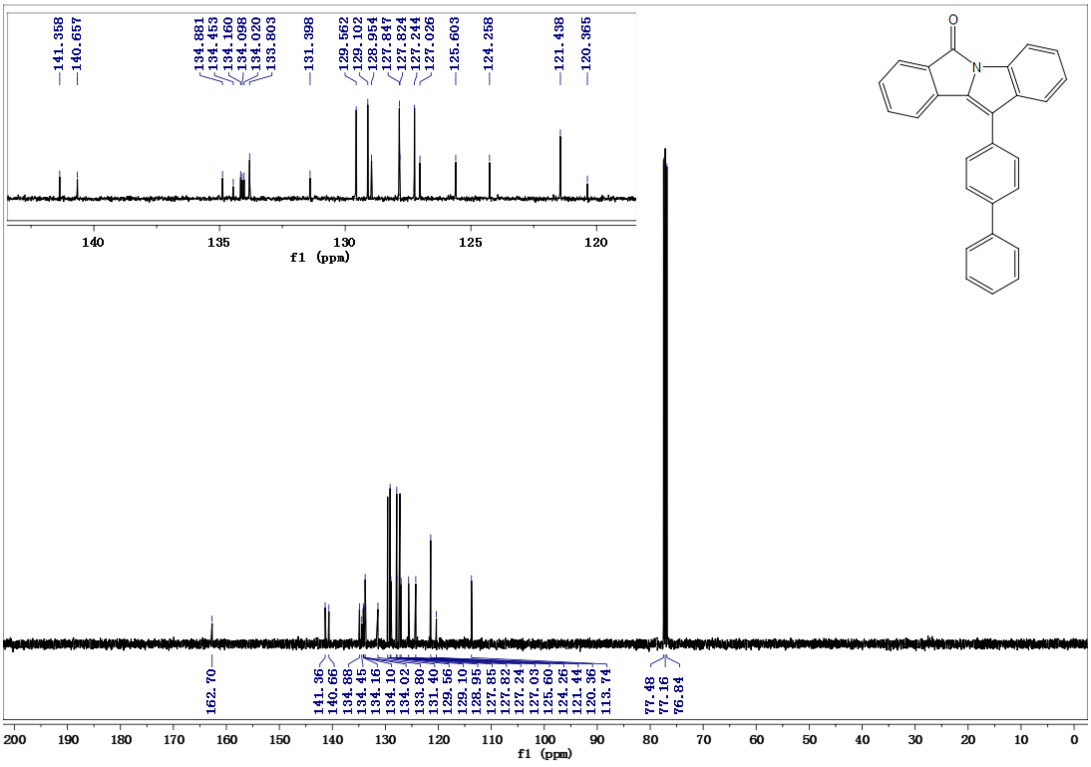

^{1}H NMR spectrum of **2aab**

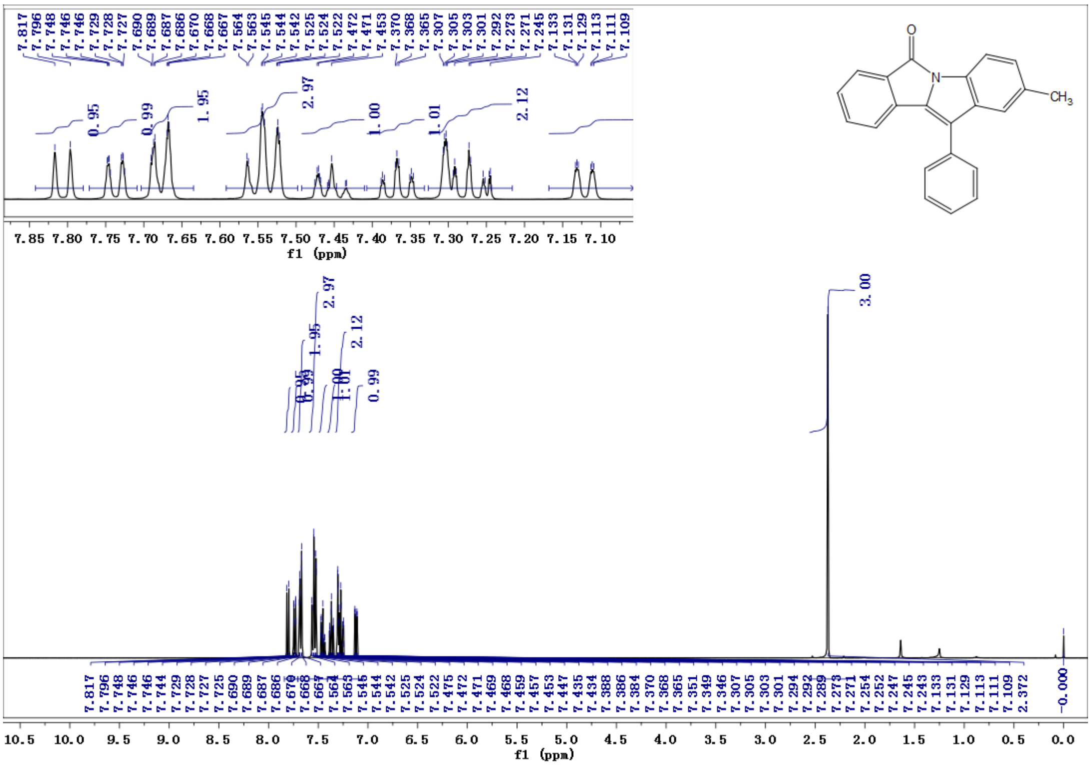

^{13}C NMR spectrum of **2aab**

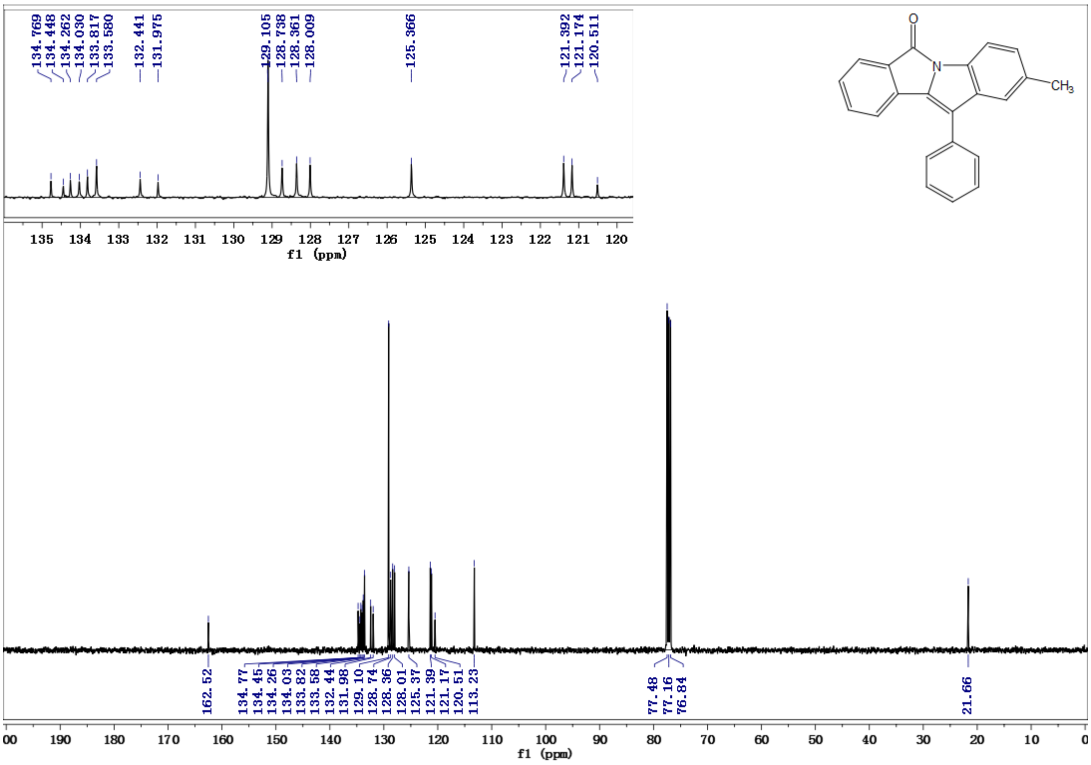

^{1}H NMR spectrum of **2abb**

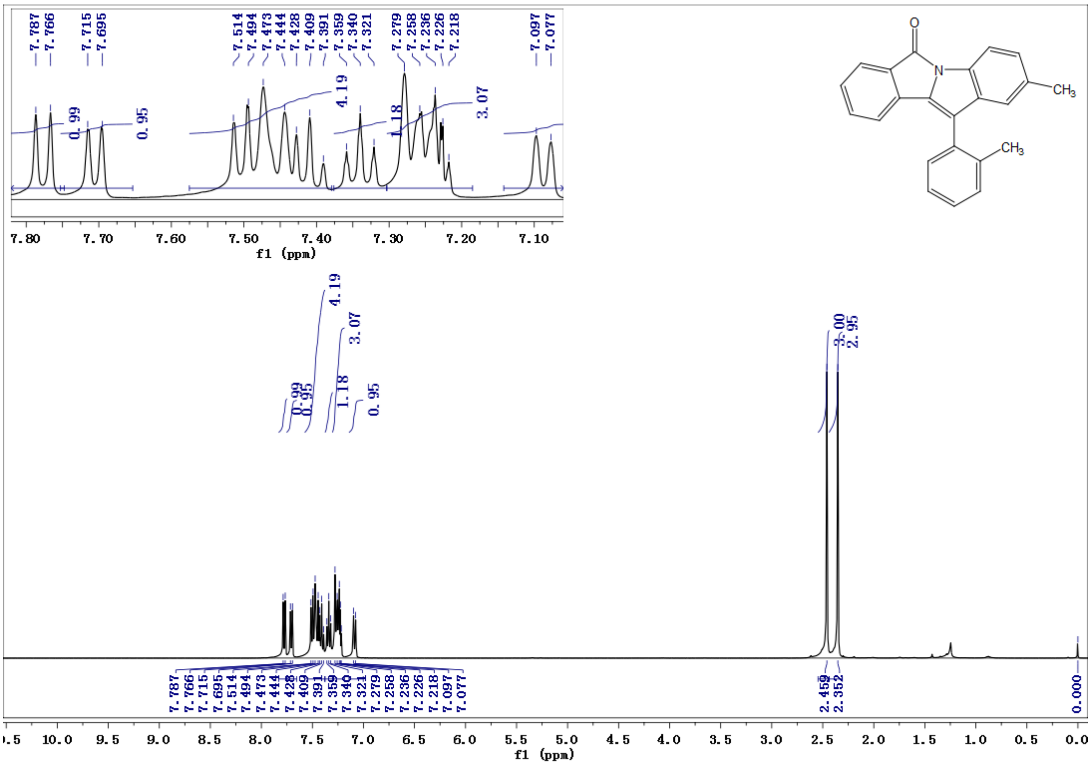

^{13}C NMR spectrum of **2abb**

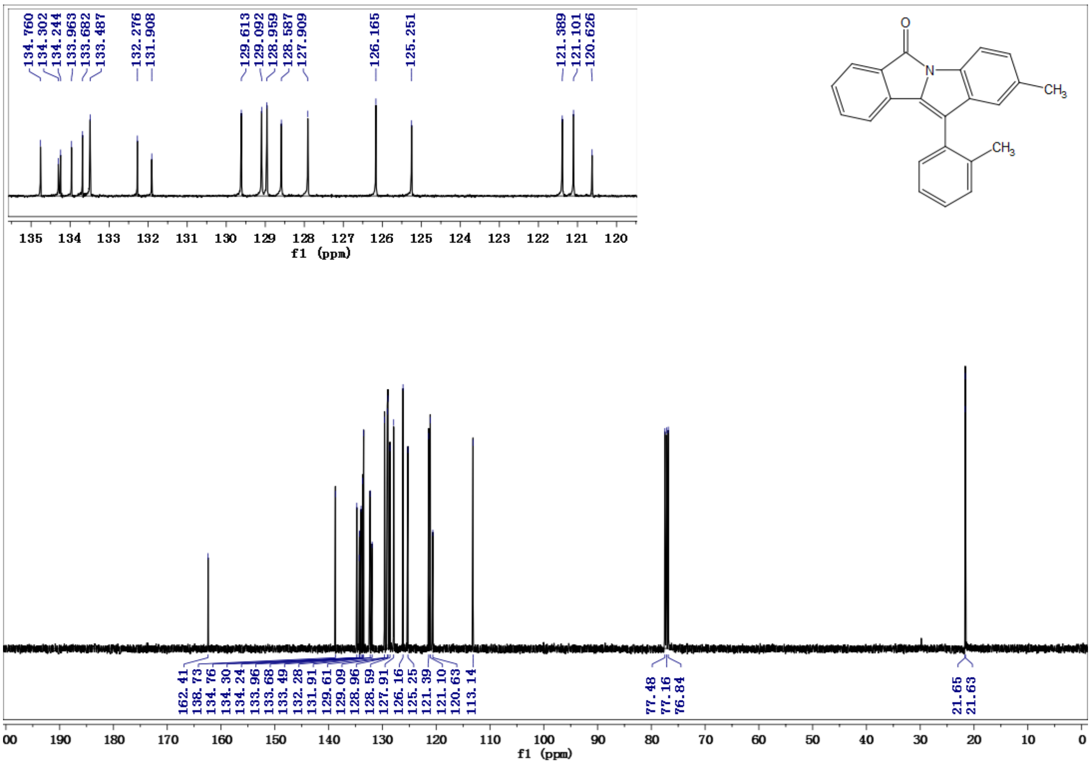

^{1}H NMR spectrum of **2acb**

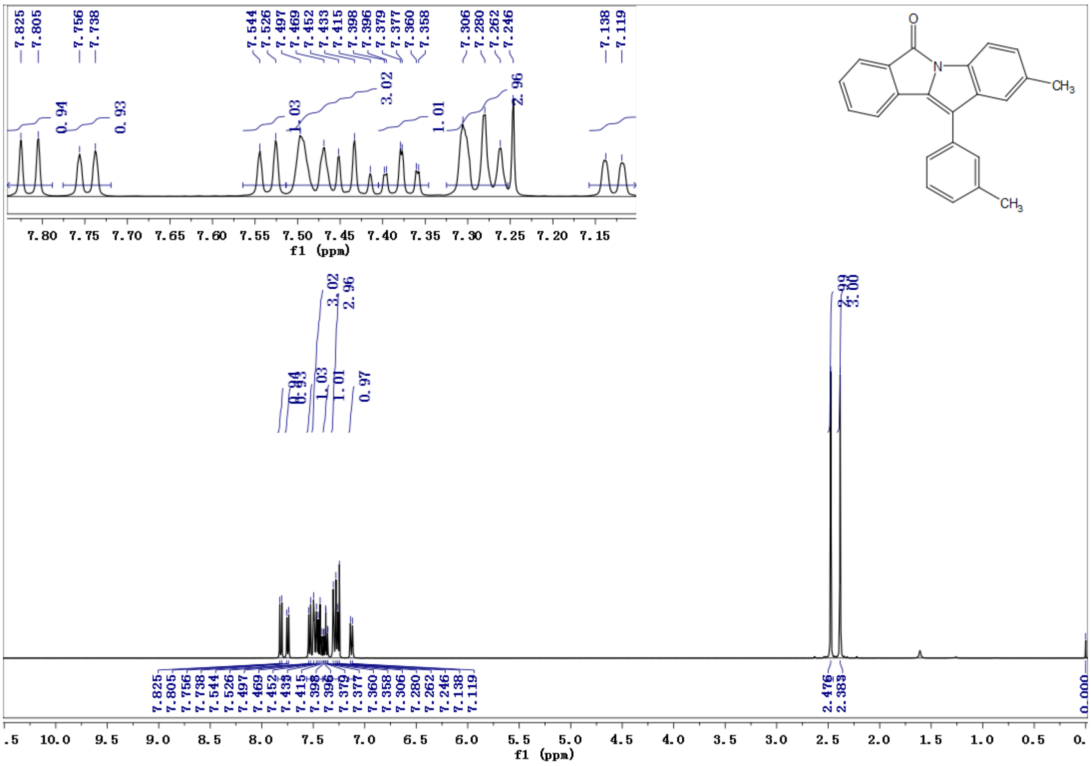

^{13}C NMR spectrum of **2acb**

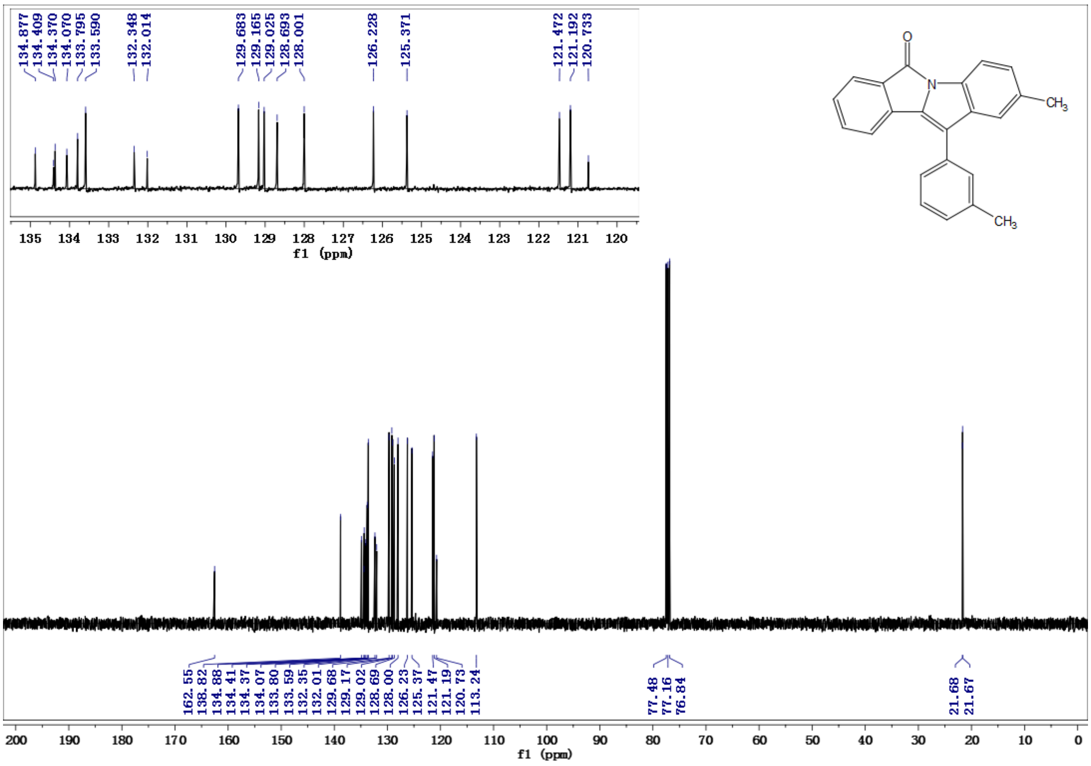

^{1}H NMR spectrum of **2adb**

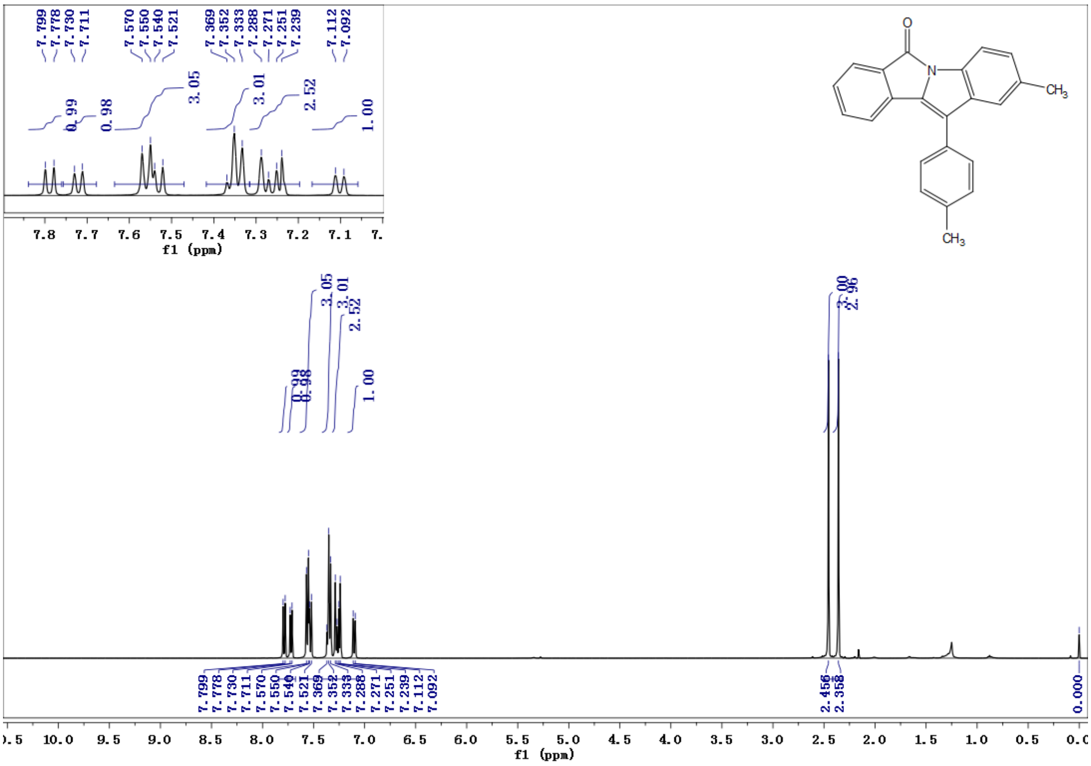

^{13}C NMR spectrum of **2adb**

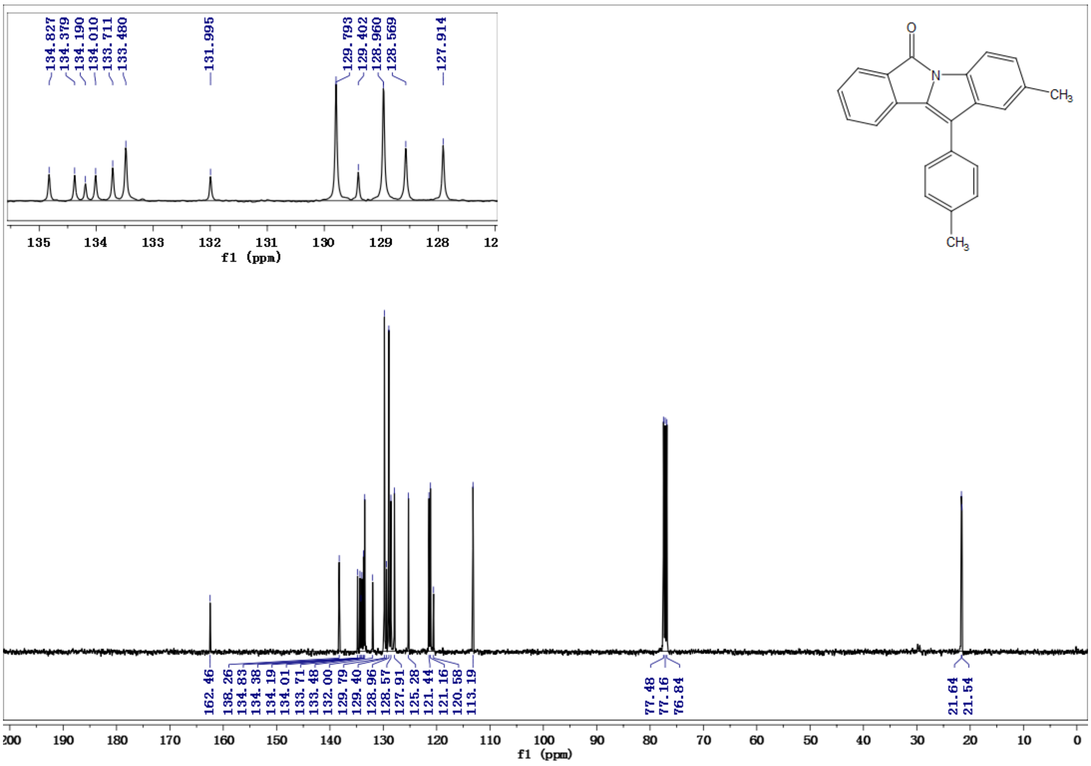

^{1}H NMR spectrum of **2aeb**

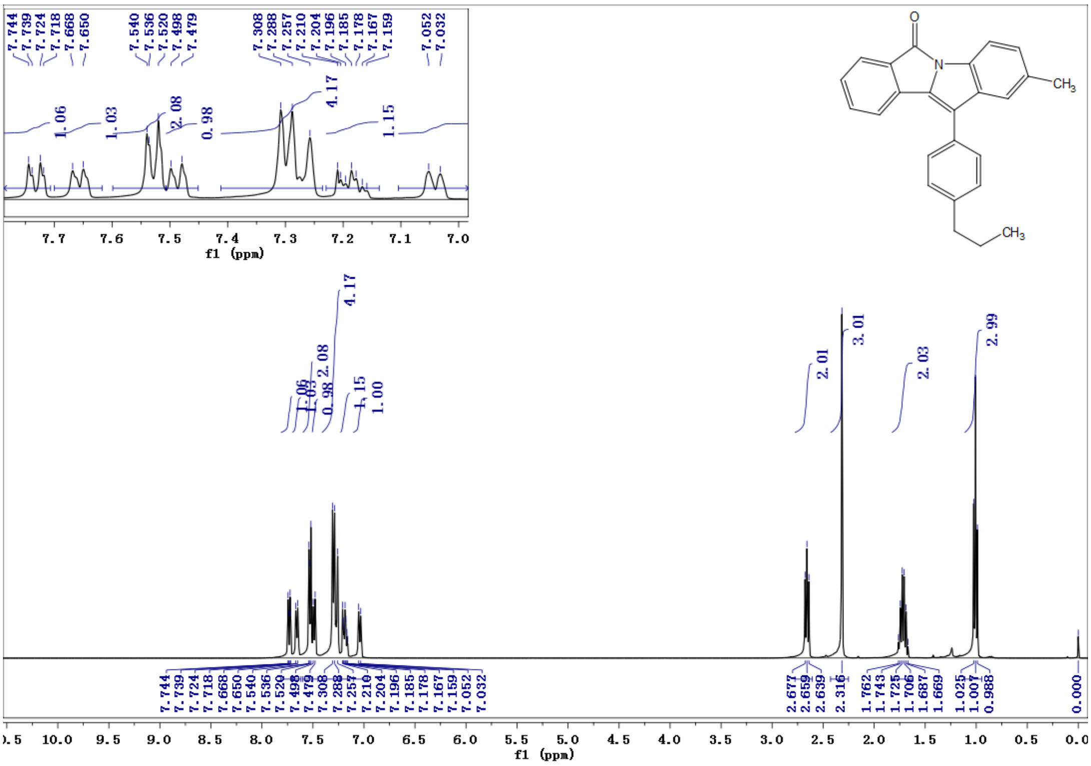

^{13}C NMR spectrum of **2aeb**

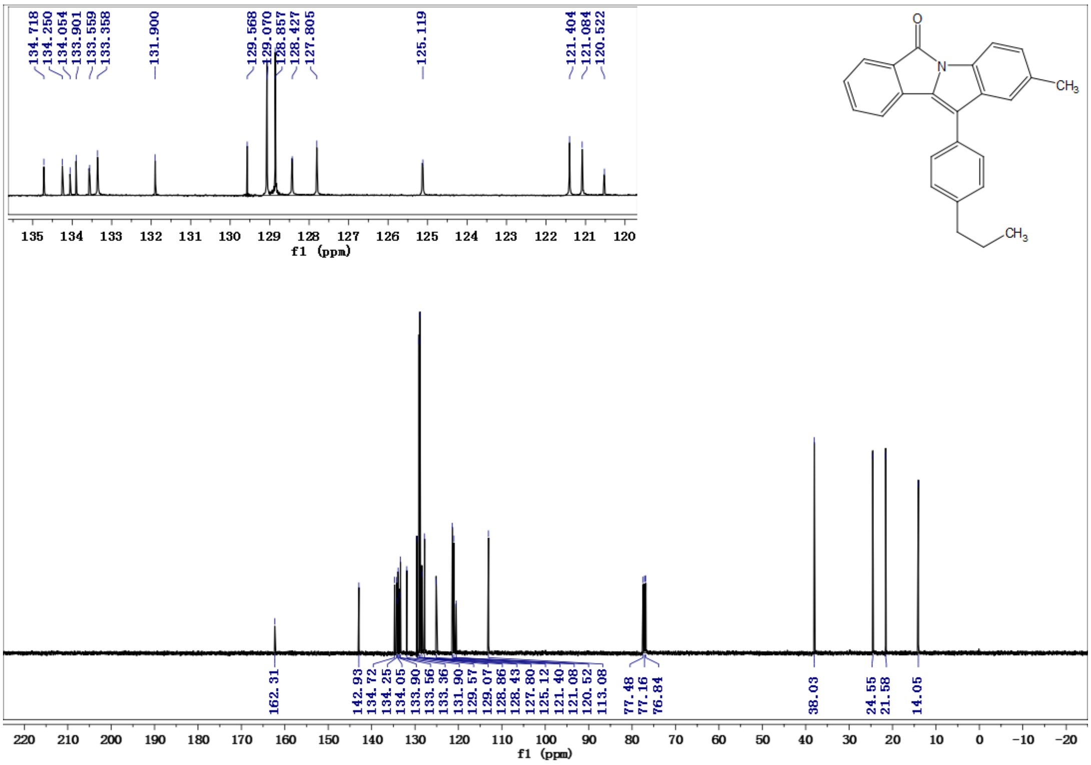

^{1}H NMR spectrum of **2afb**

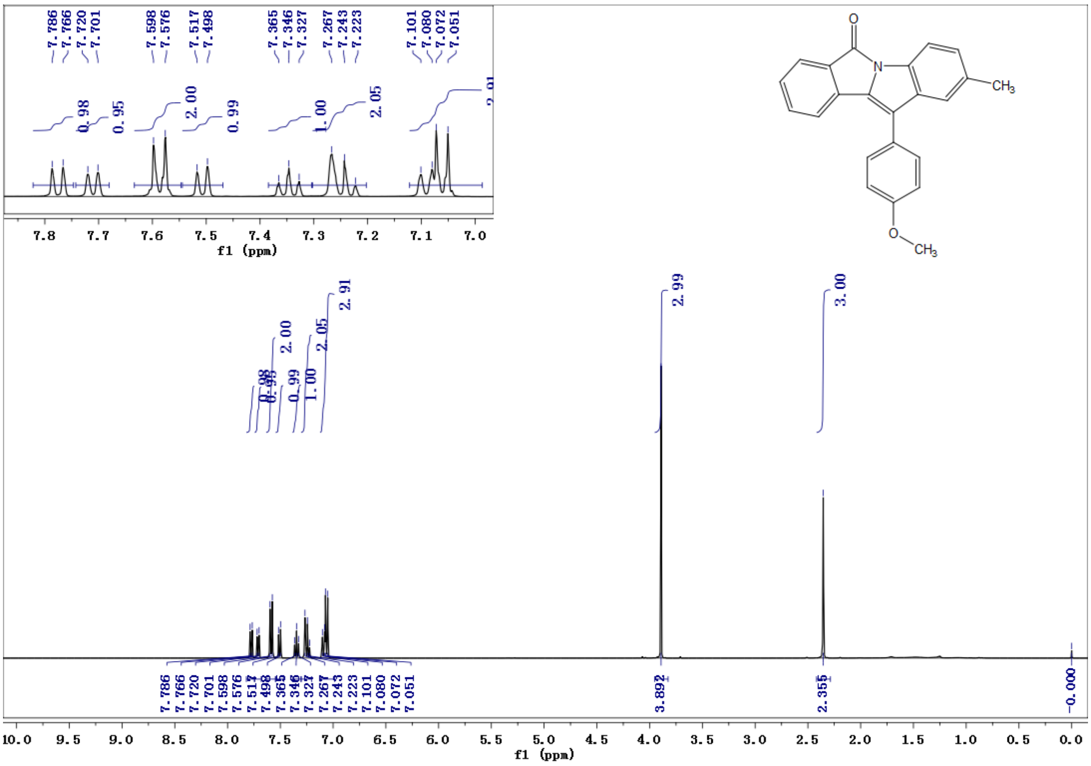

^{13}C NMR spectrum of **2afb**

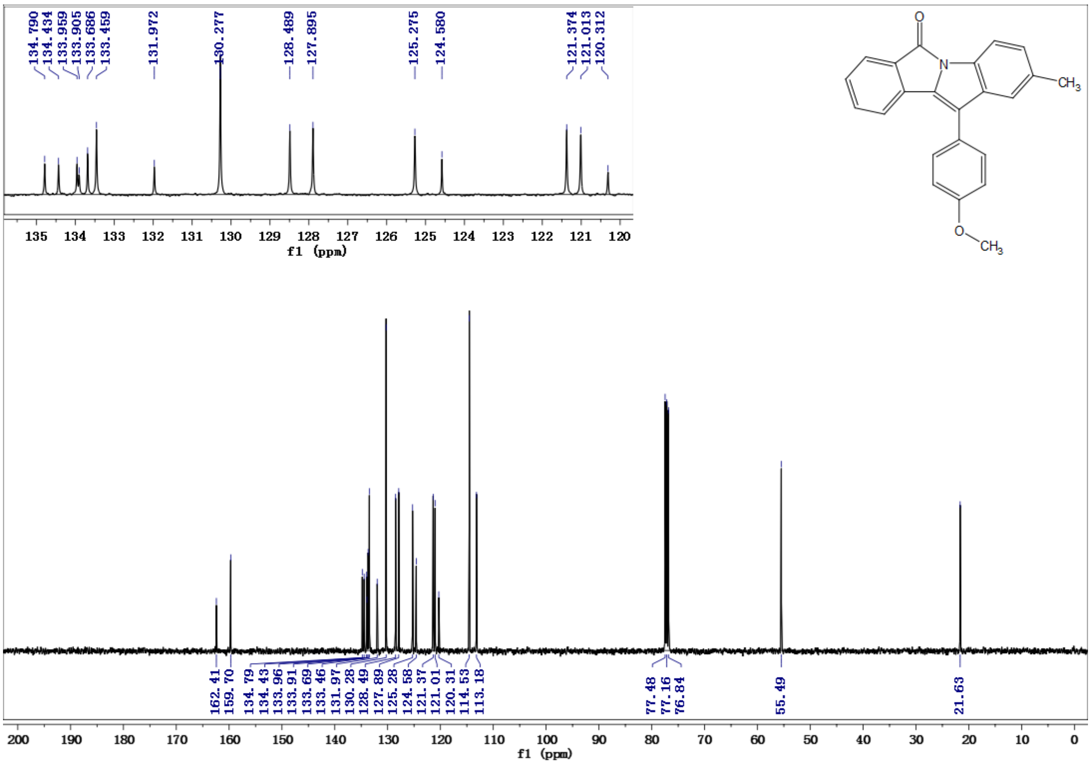

^{1}H NMR spectrum of **2agb**

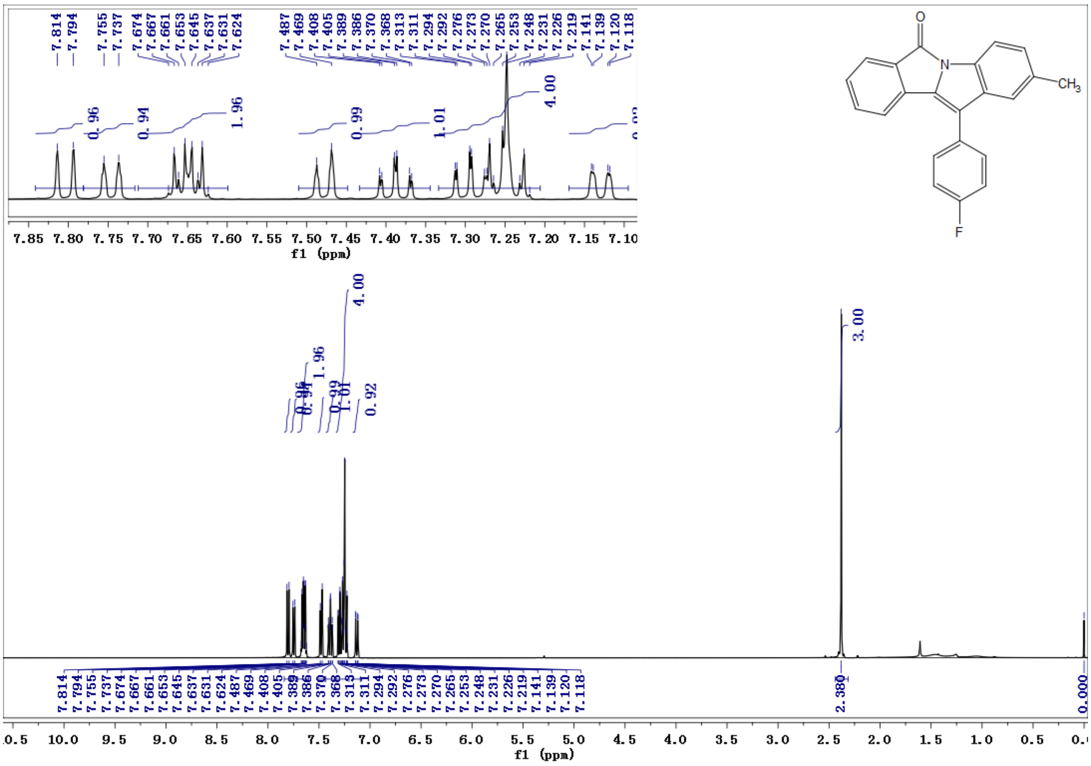

^{13}C NMR spectrum of **2agb**

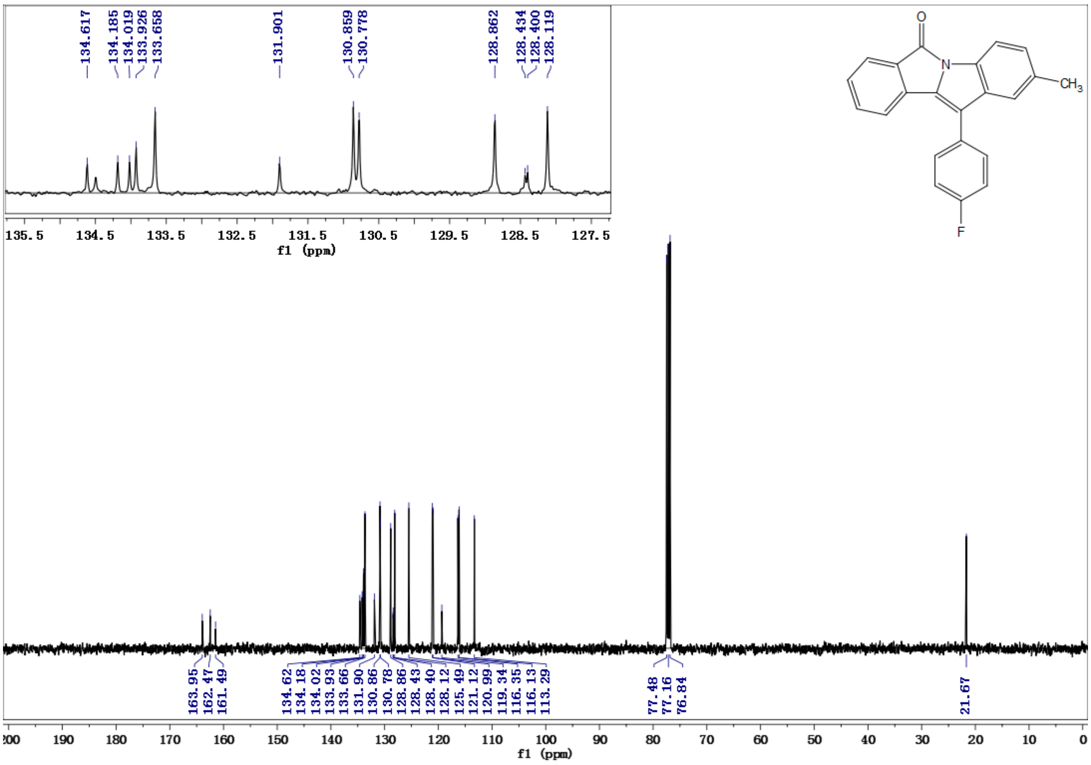

^{19}F NMR spectrum of **2agb**

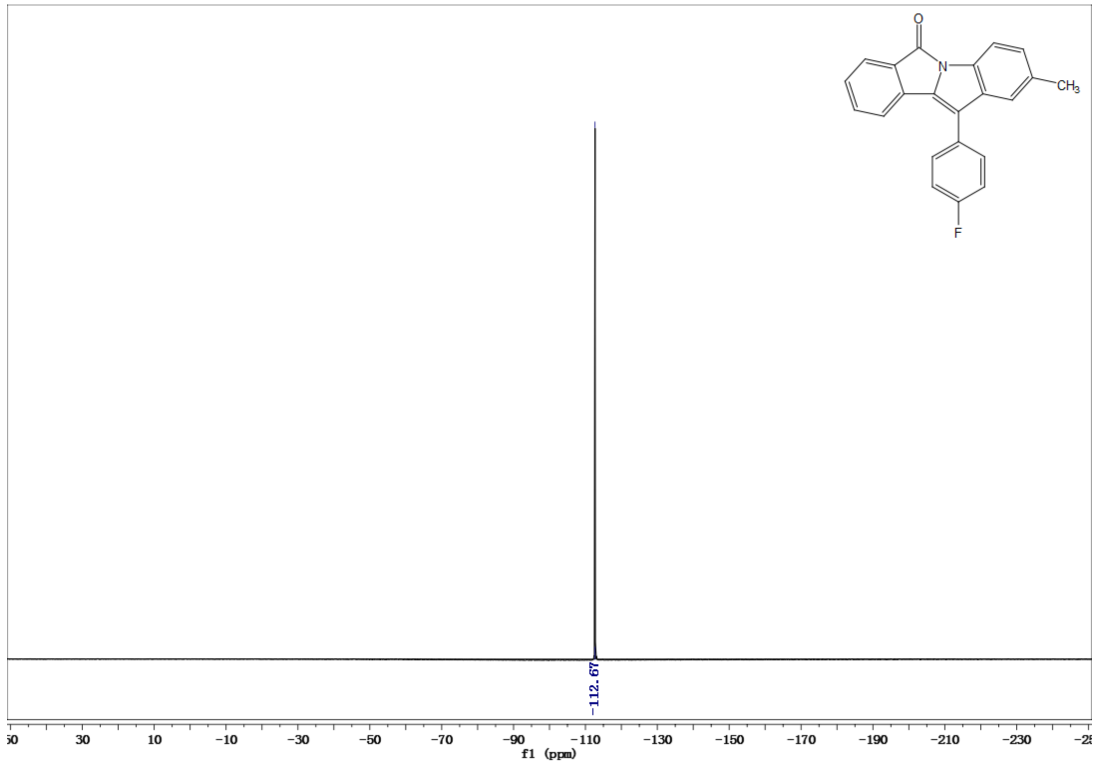

^{1}H NMR spectrum of **2ahb**

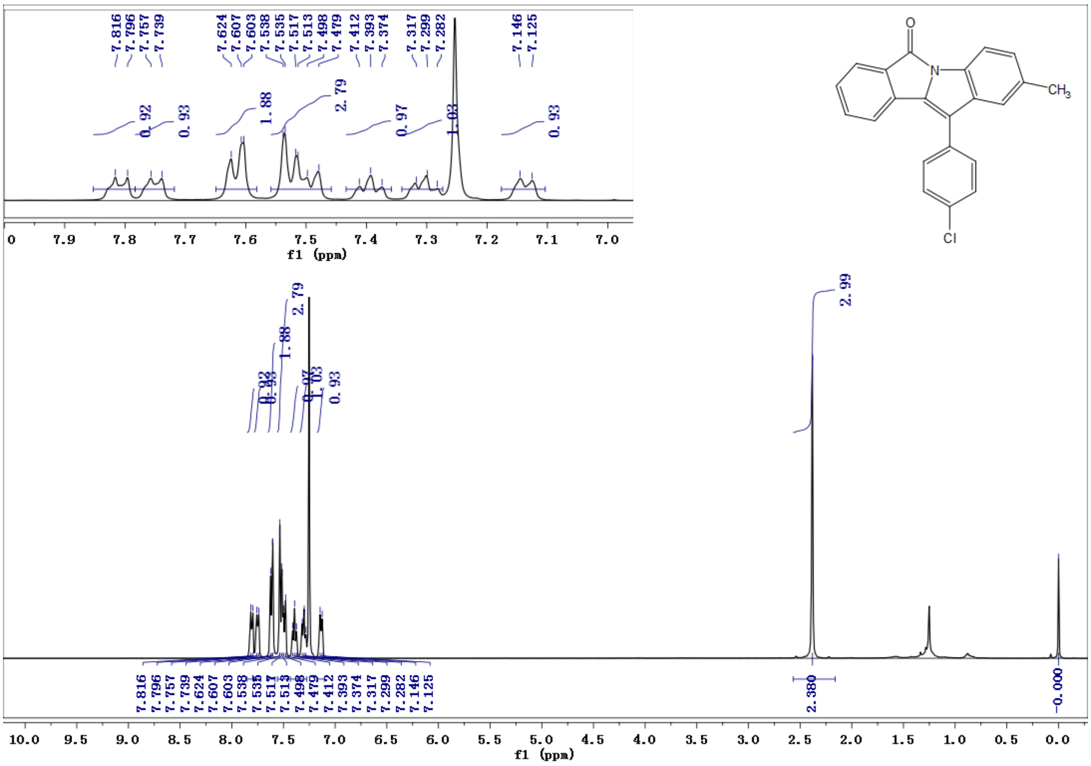

^{13}C NMR spectrum of **2ahb**

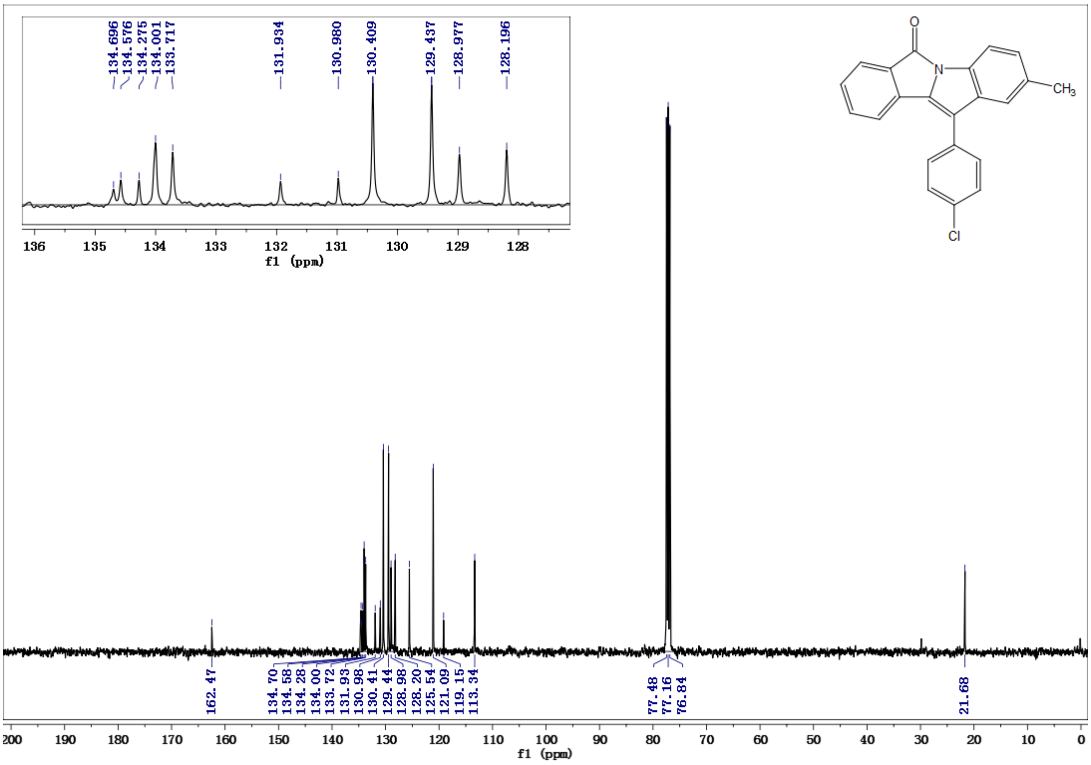

^{1}H NMR spectrum of **2aib**

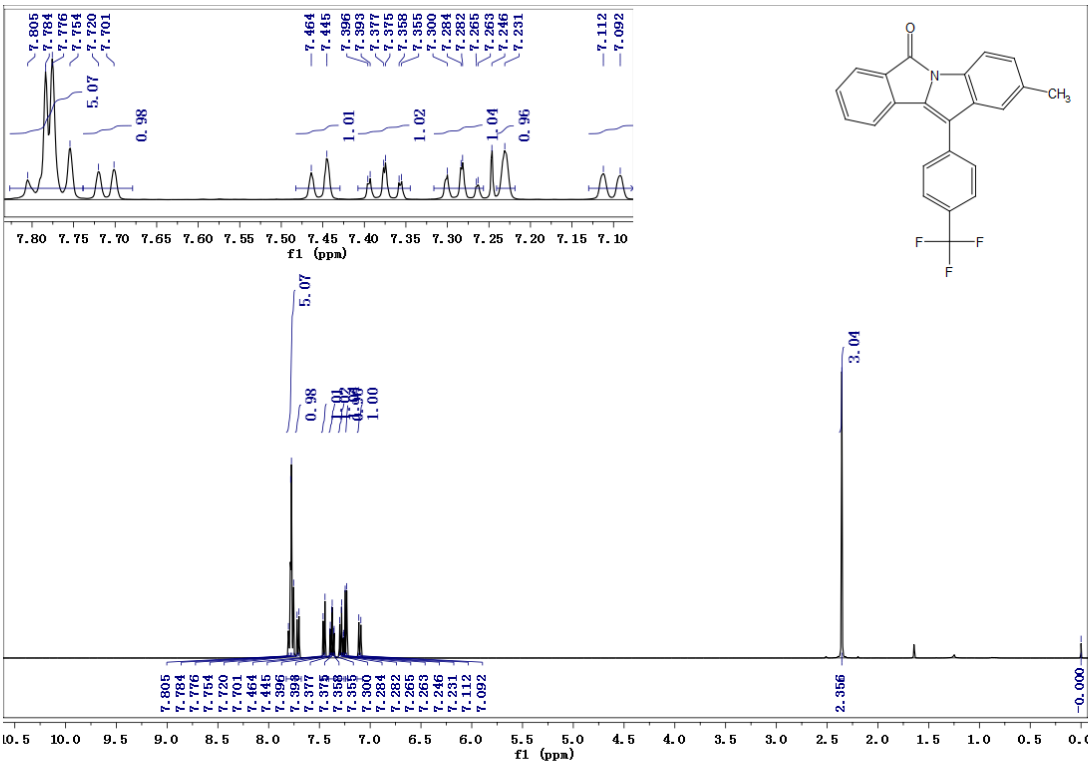

^{13}C NMR spectrum of **2aib**

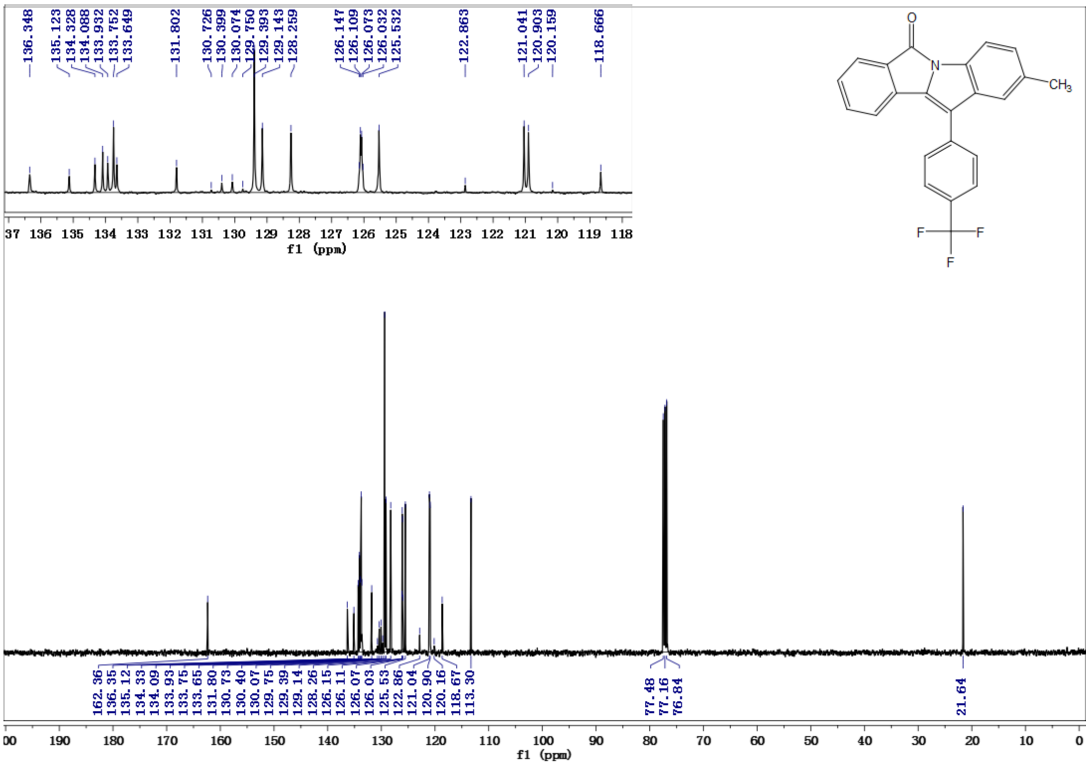

^{19}F NMR spectrum of **2aib**

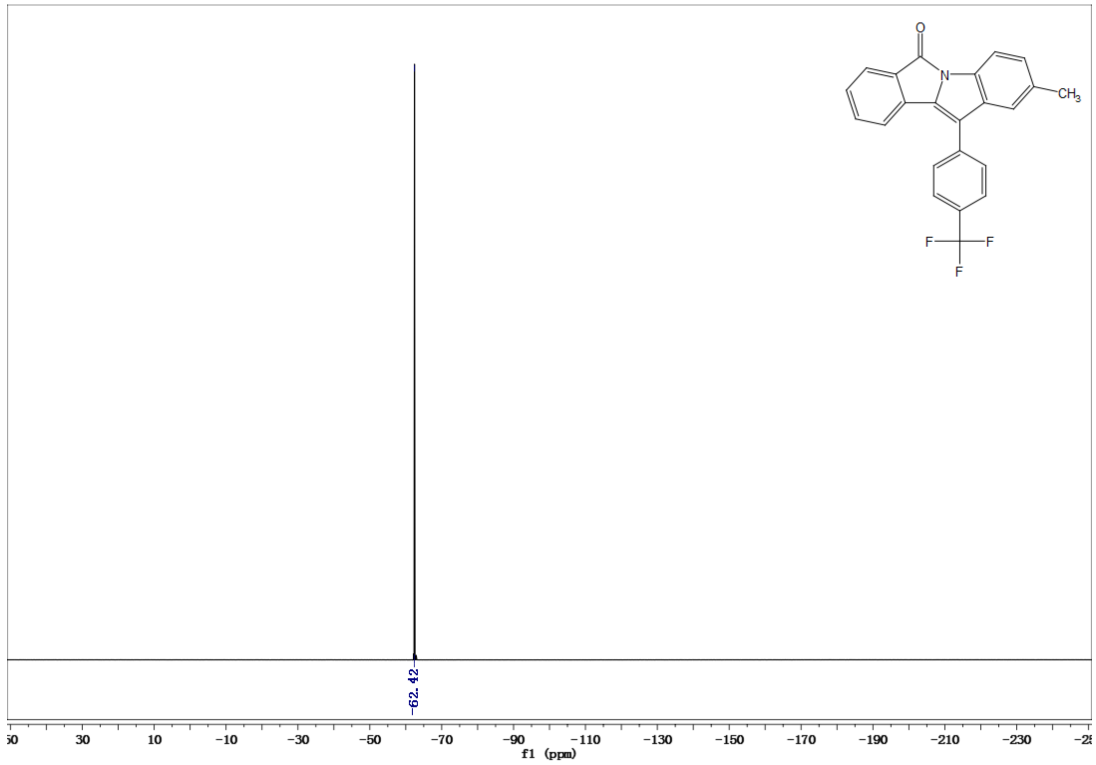

^{1}H NMR spectrum of **2ajb**

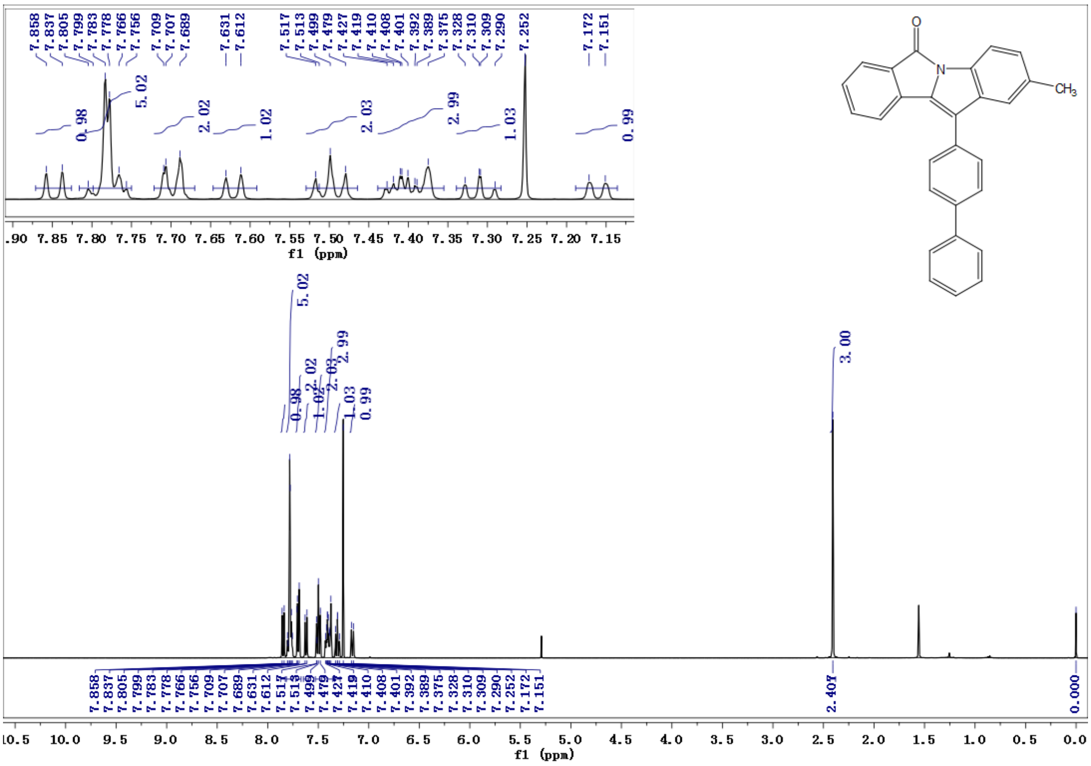

^{13}C NMR spectrum of **2ajb**

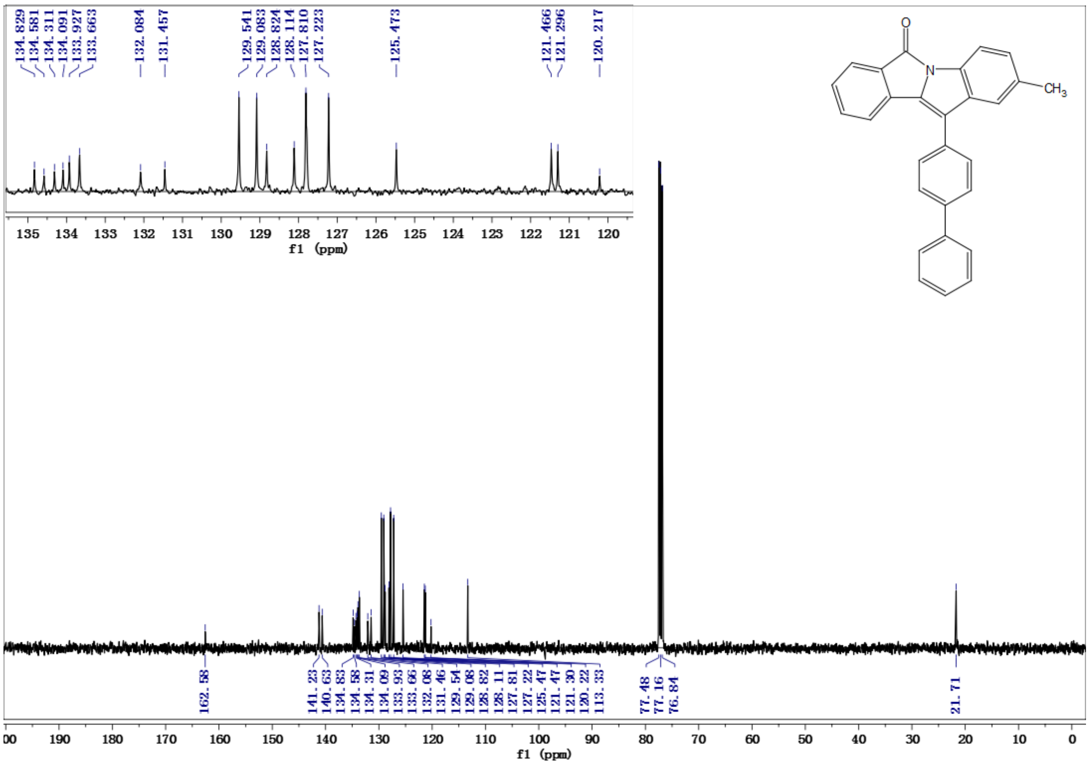

^{1}H NMR spectrum of **2aac**

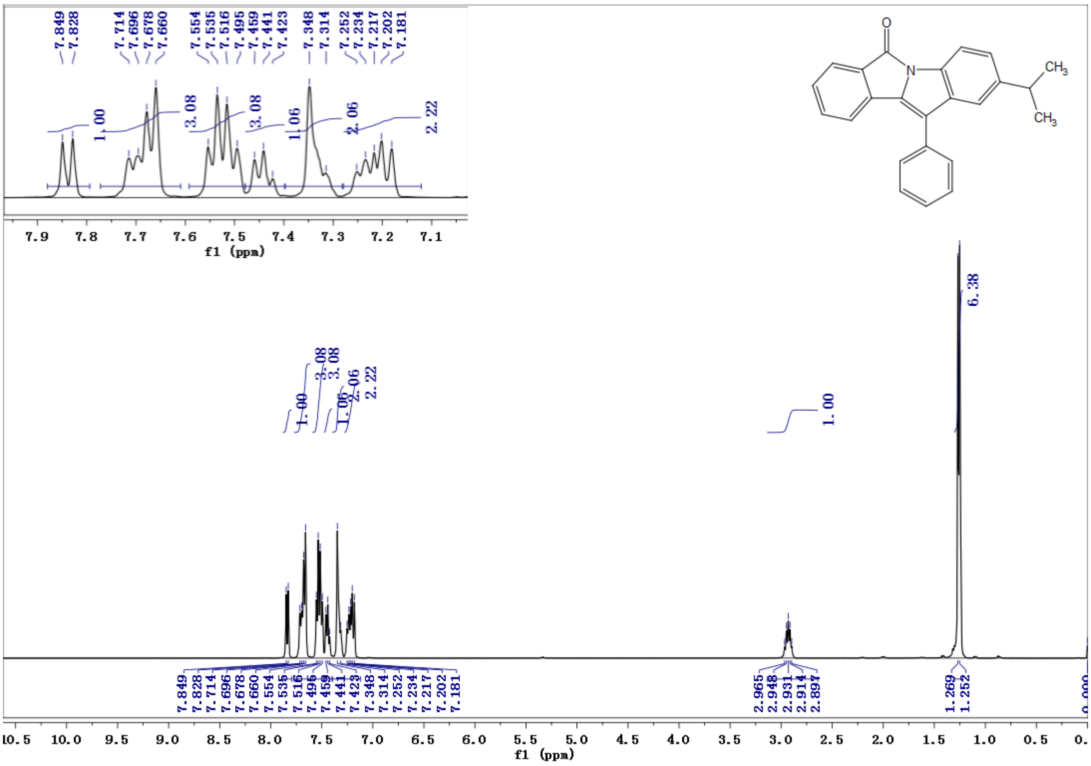

^{13}C NMR spectrum of **2aac**

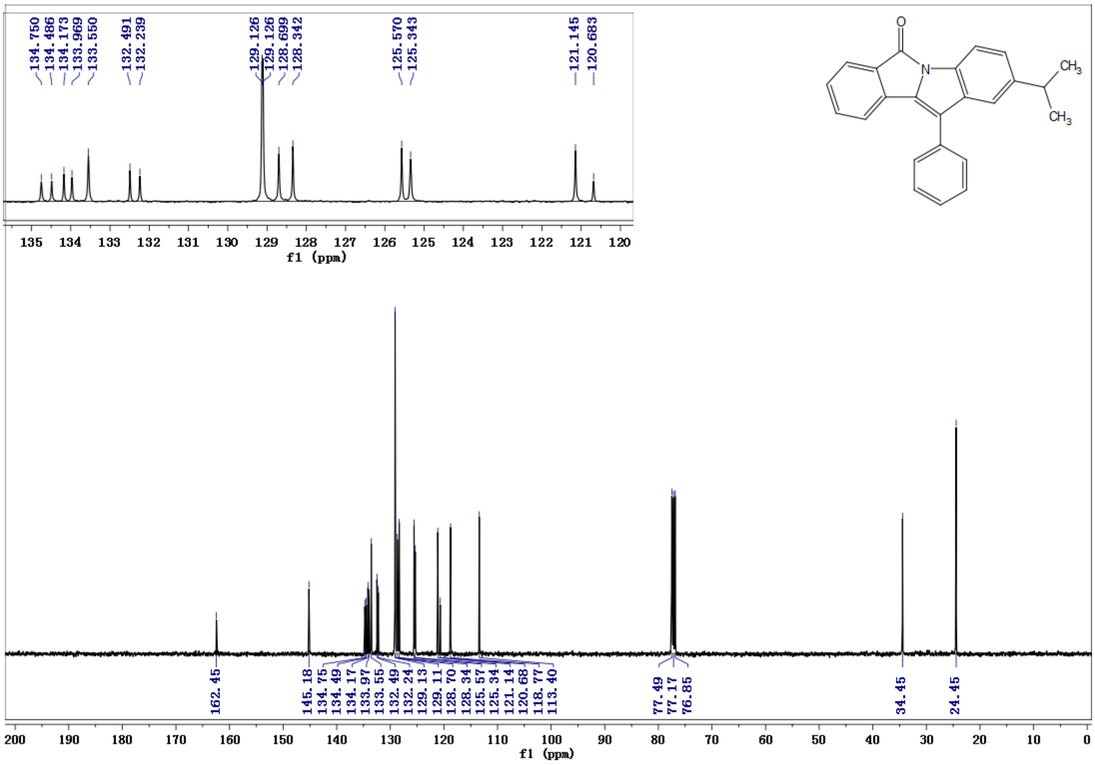

^{1}H NMR spectrum of **2aad**

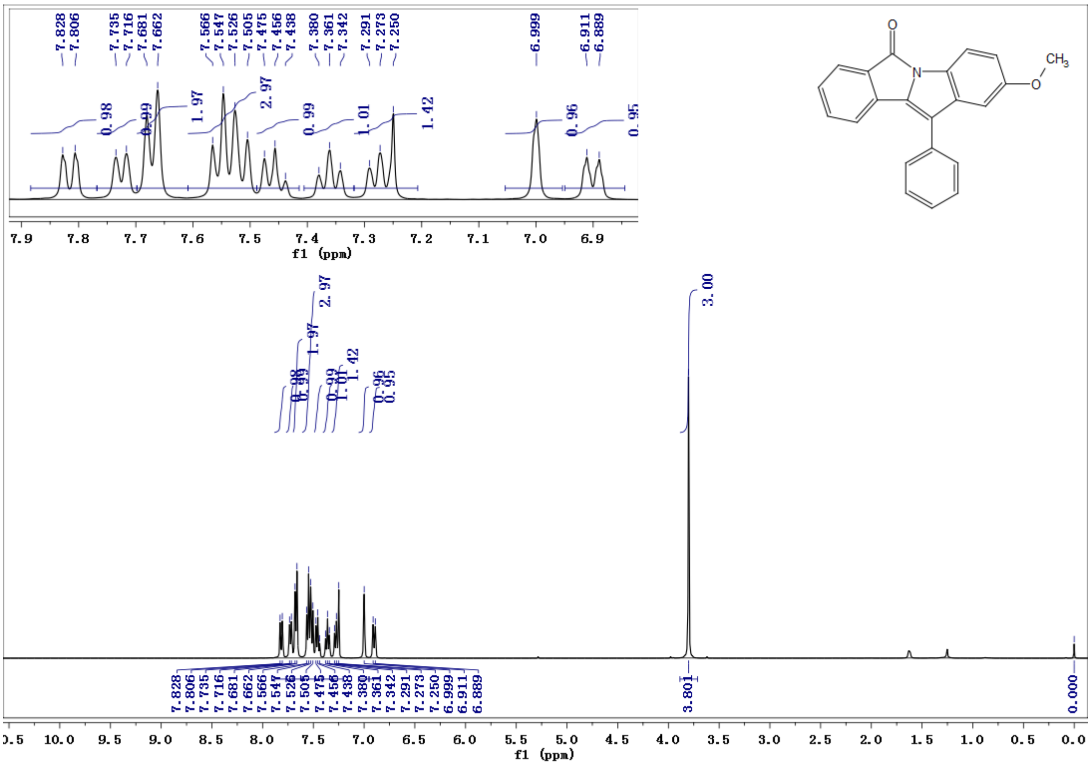

^{13}C NMR spectrum of **2aad**

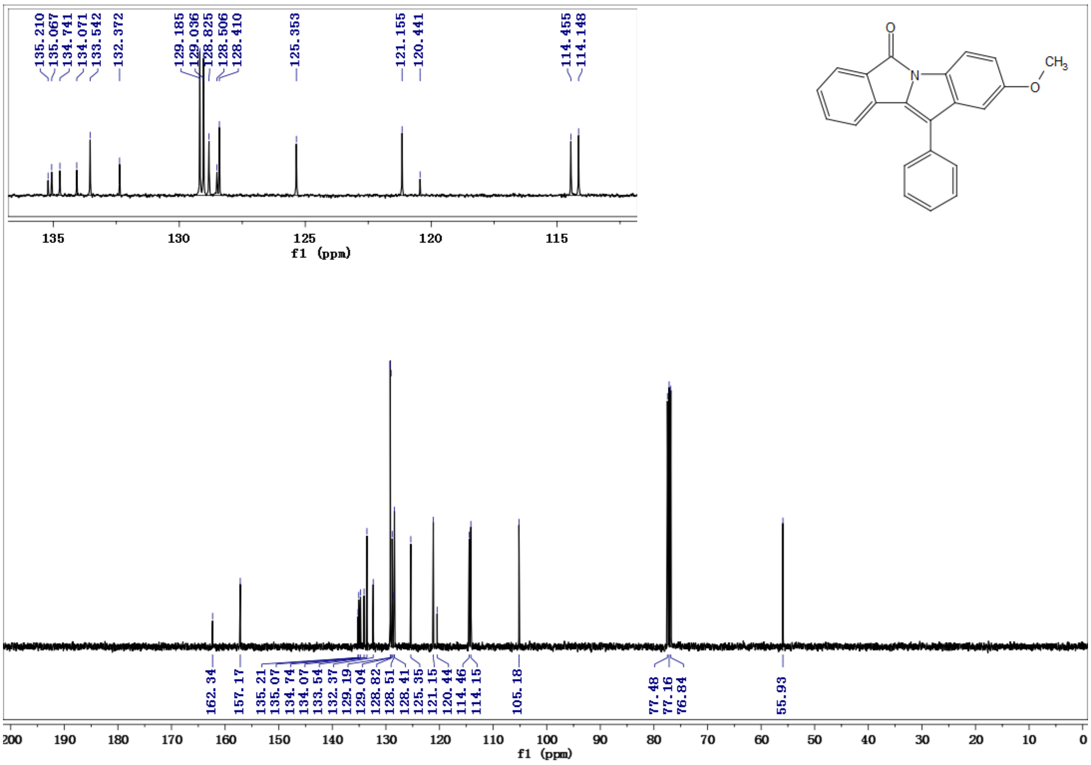

^{1}H NMR spectrum of **2aae**

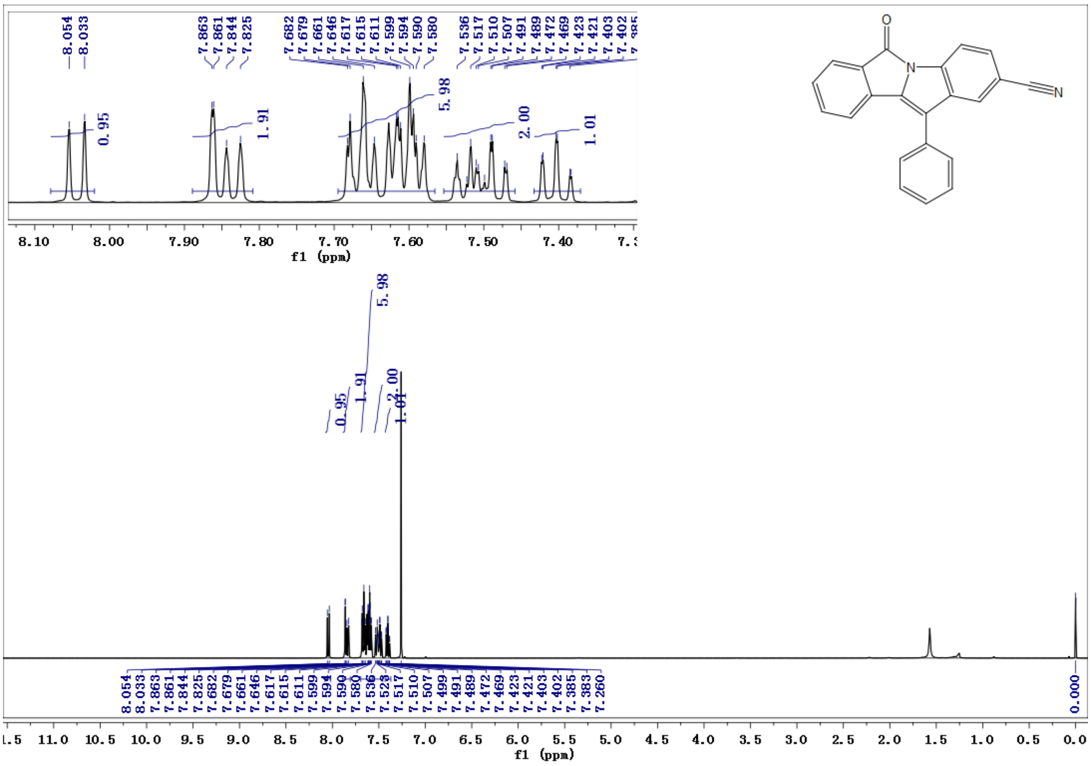

^{13}C NMR spectrum of **2aae**

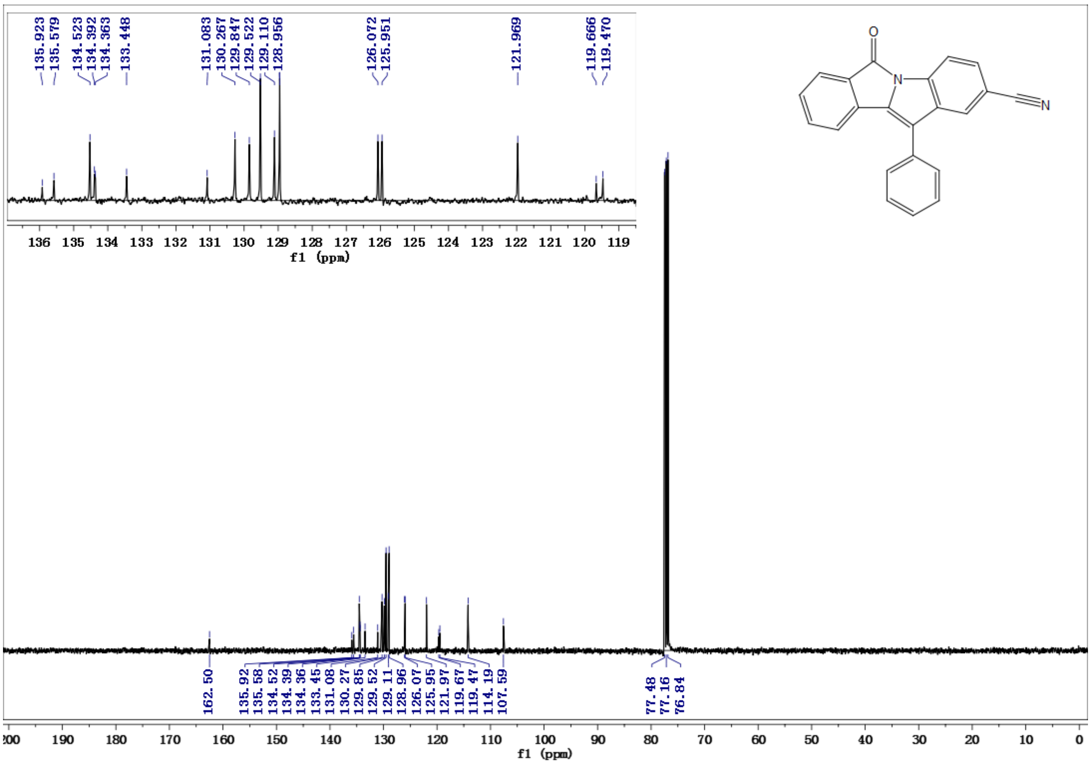

^{1}H NMR spectrum of **2afd**

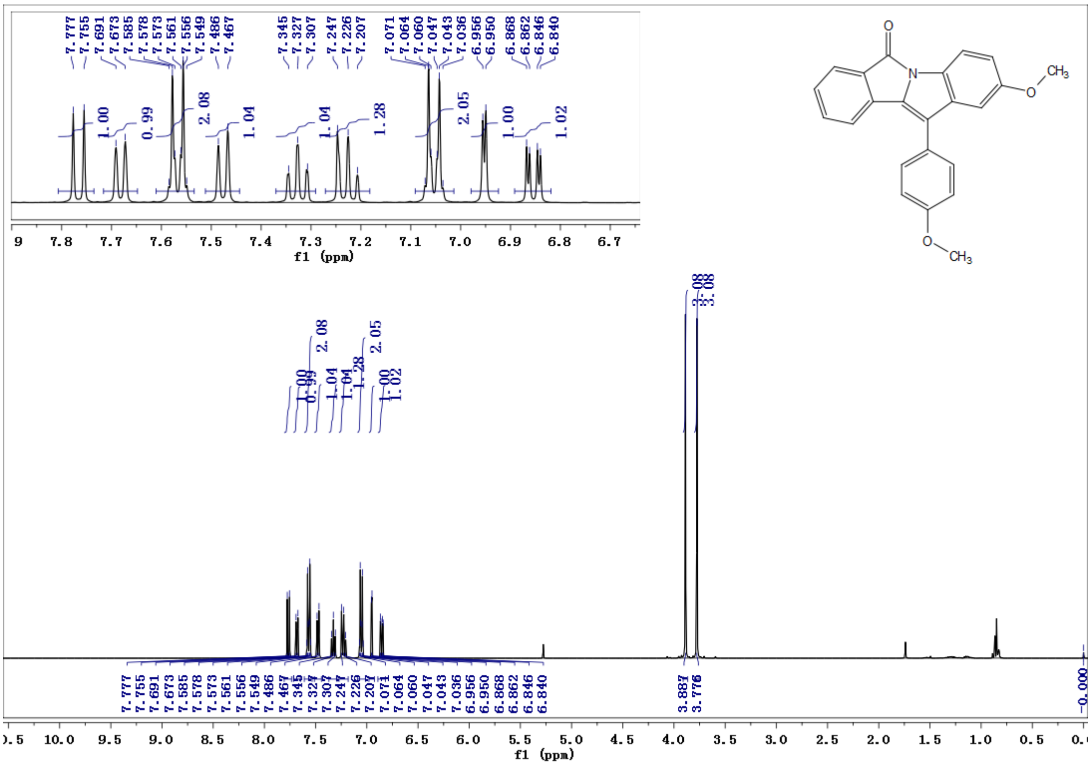

^{13}C NMR spectrum of **2afd**

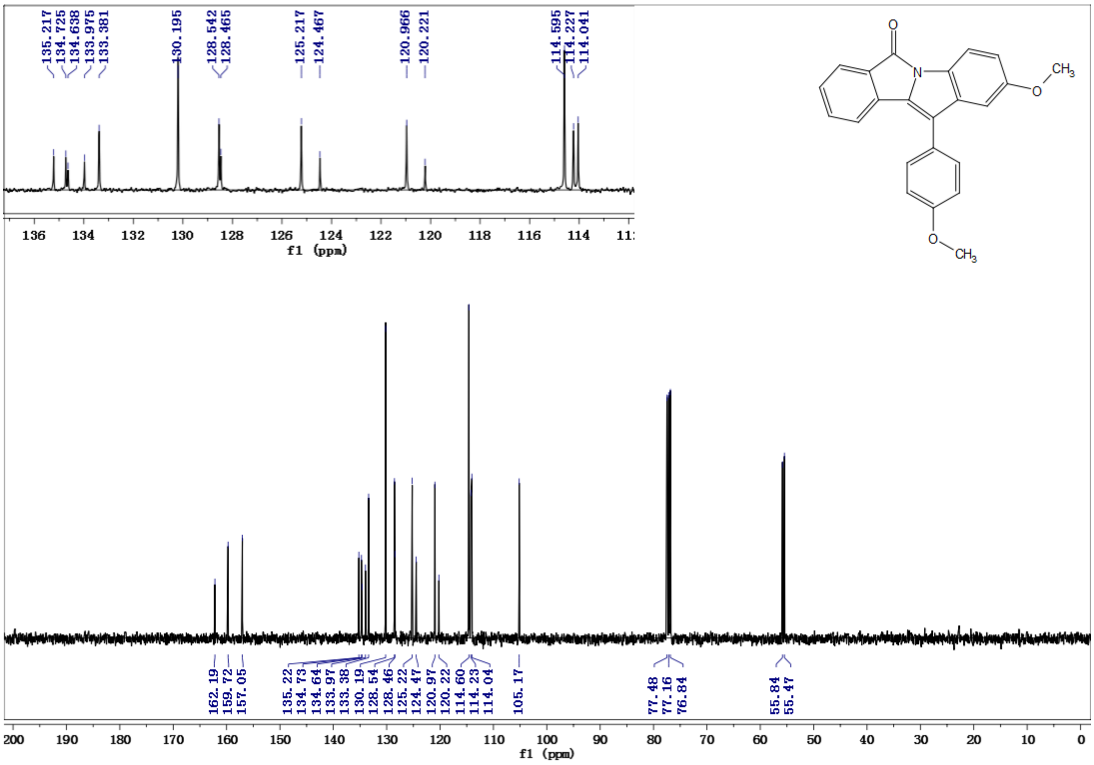

^{1}H NMR spectrum of **2afe**

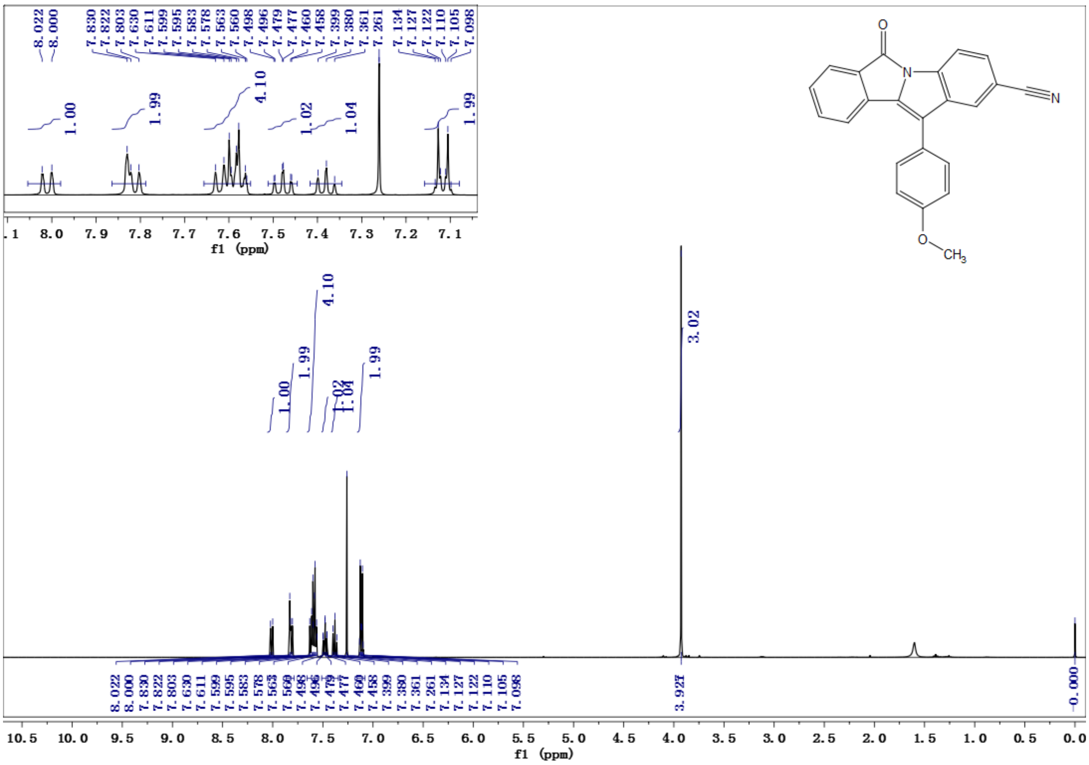

^{13}C NMR spectrum of **2afe**

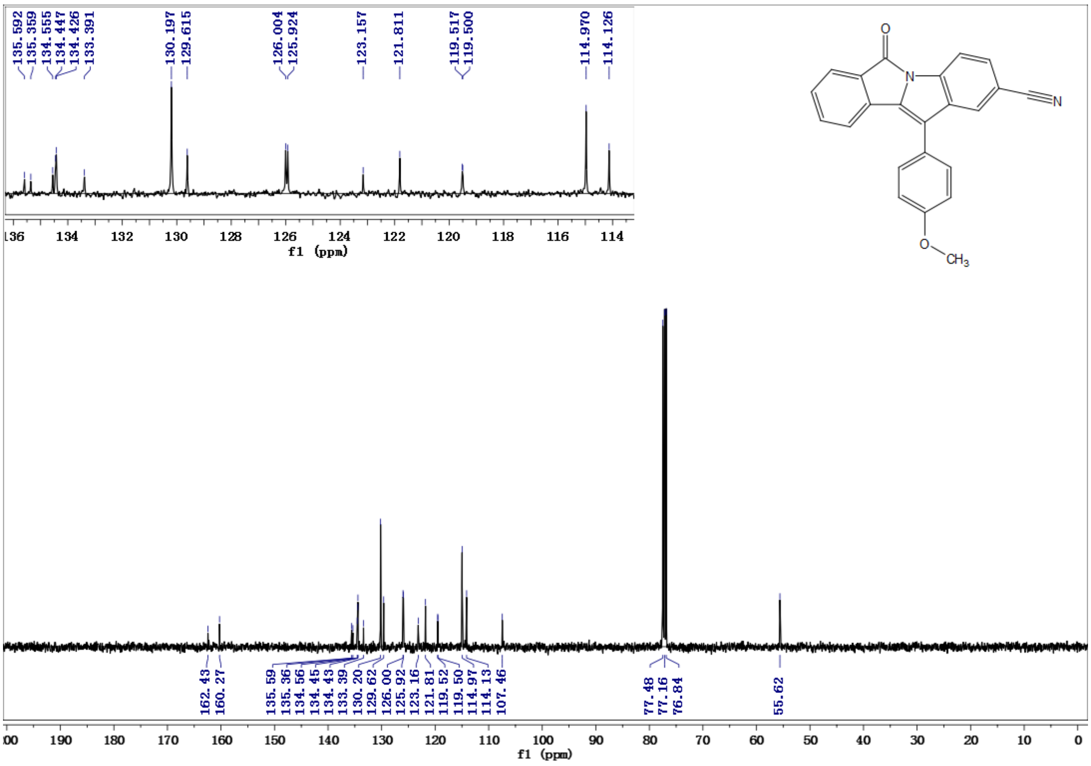

^{1}H NMR spectrum of **2aff**

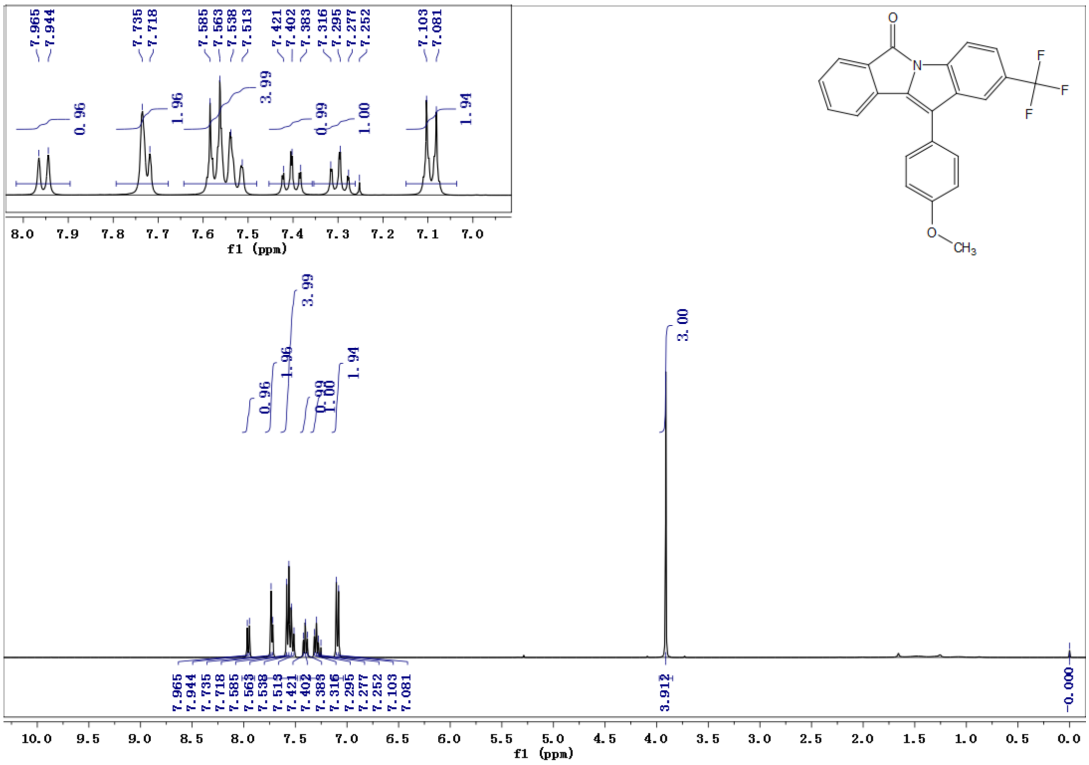

^{13}C NMR spectrum of **2aff**

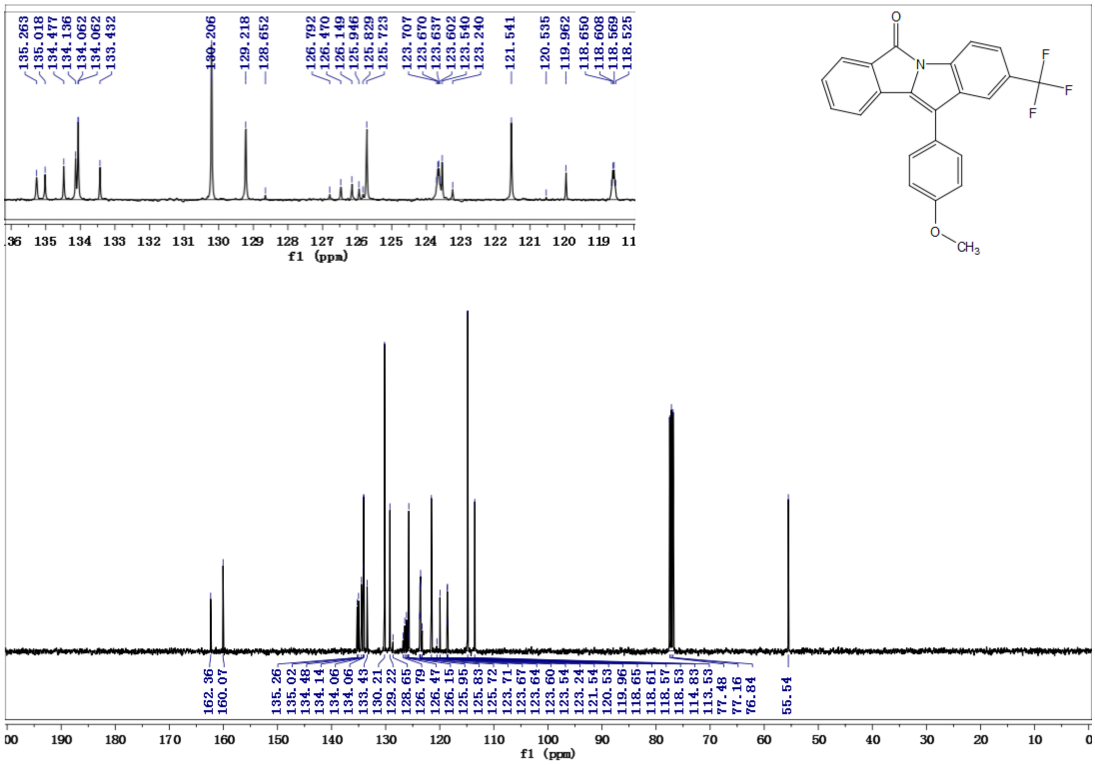

^{19}F NMR spectrum of **2aff**

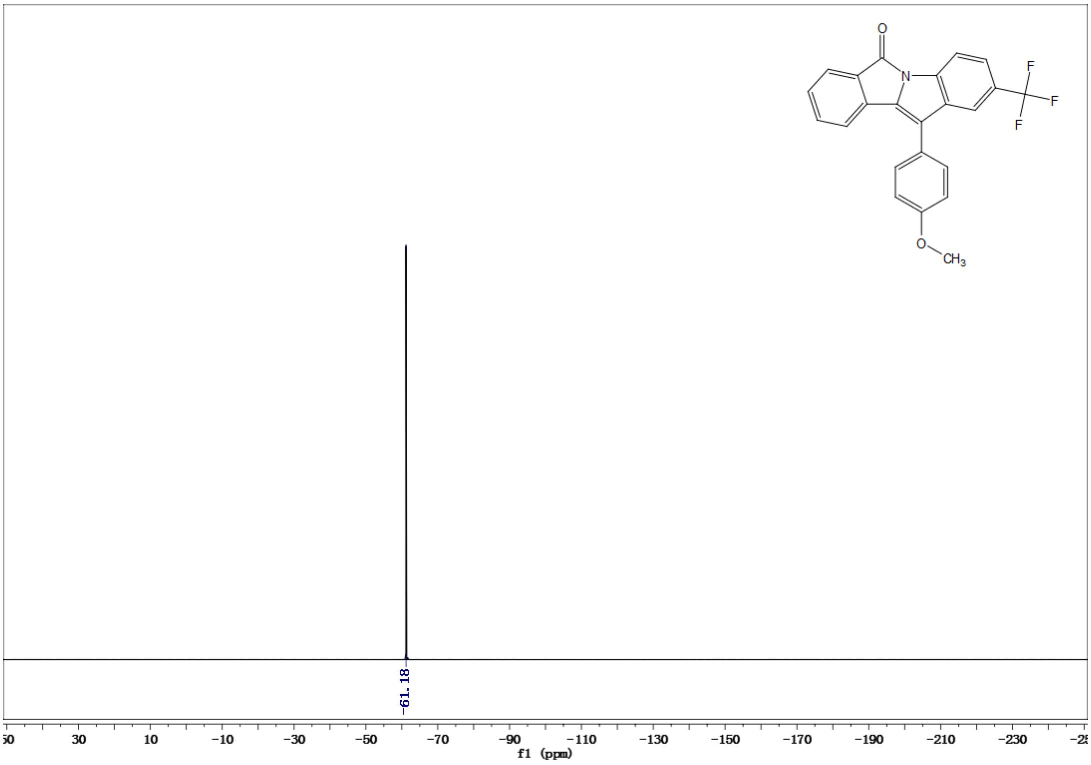

^{1}H NMR spectrum of **2baa**

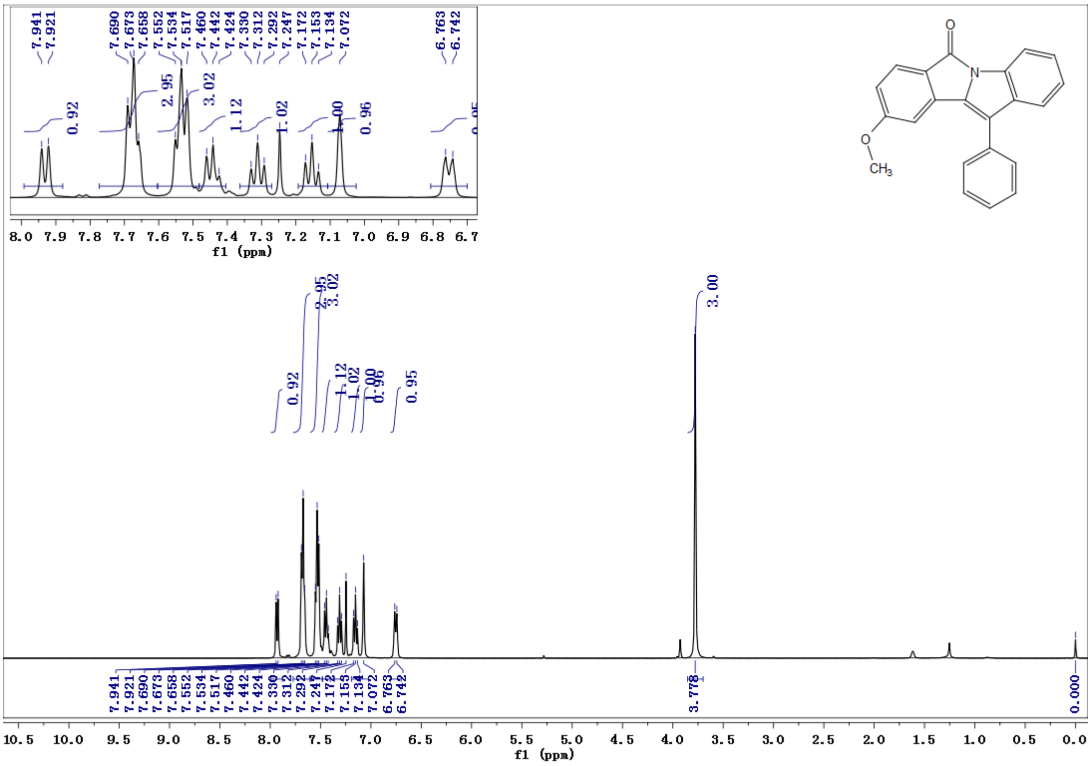

^{13}C NMR spectrum of **2baa**

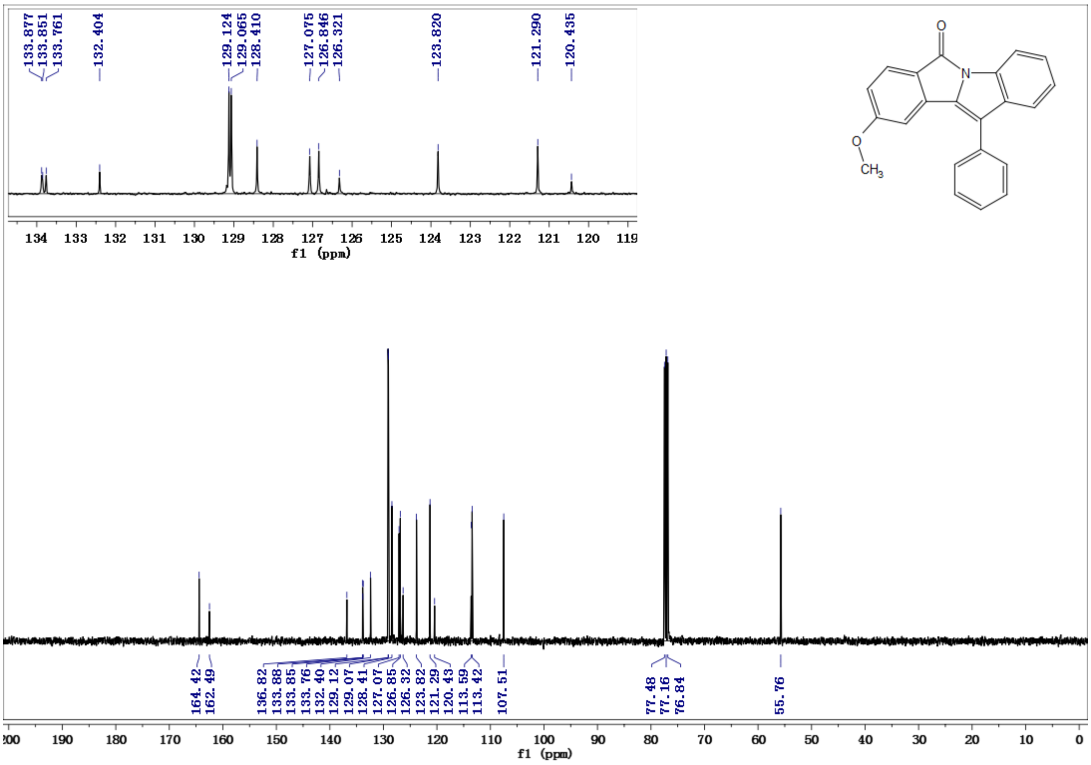

^{1}H NMR spectrum of **2caa**

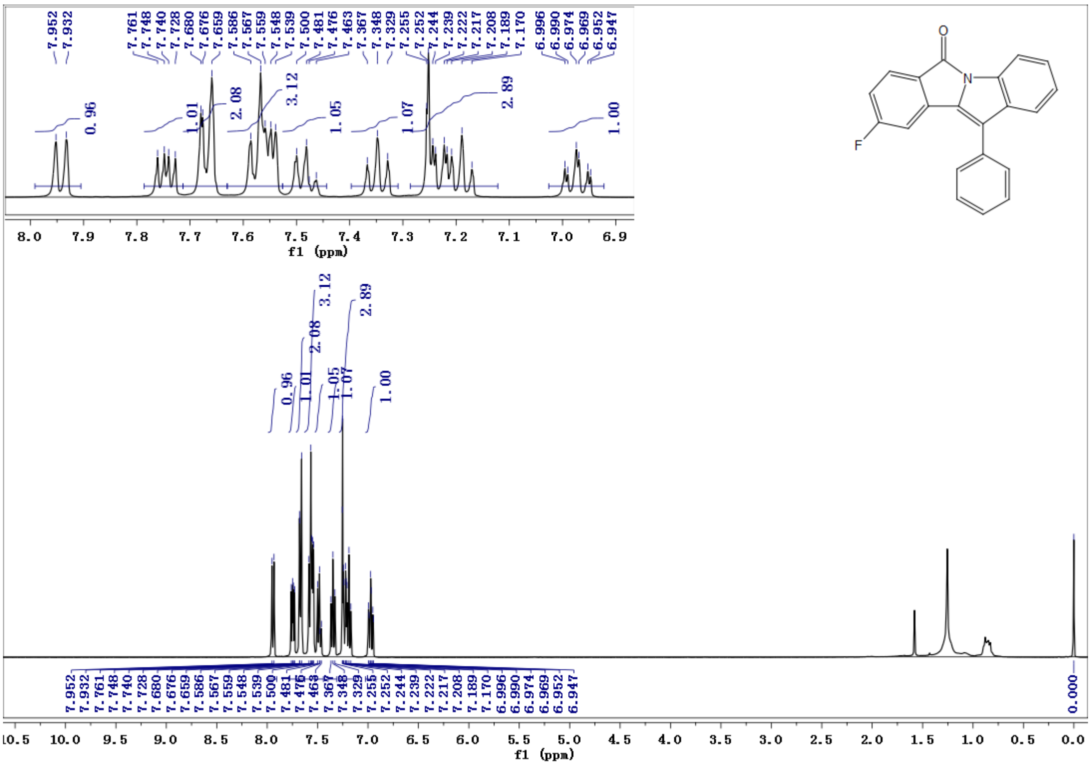

^{13}C NMR spectrum of **2caa**

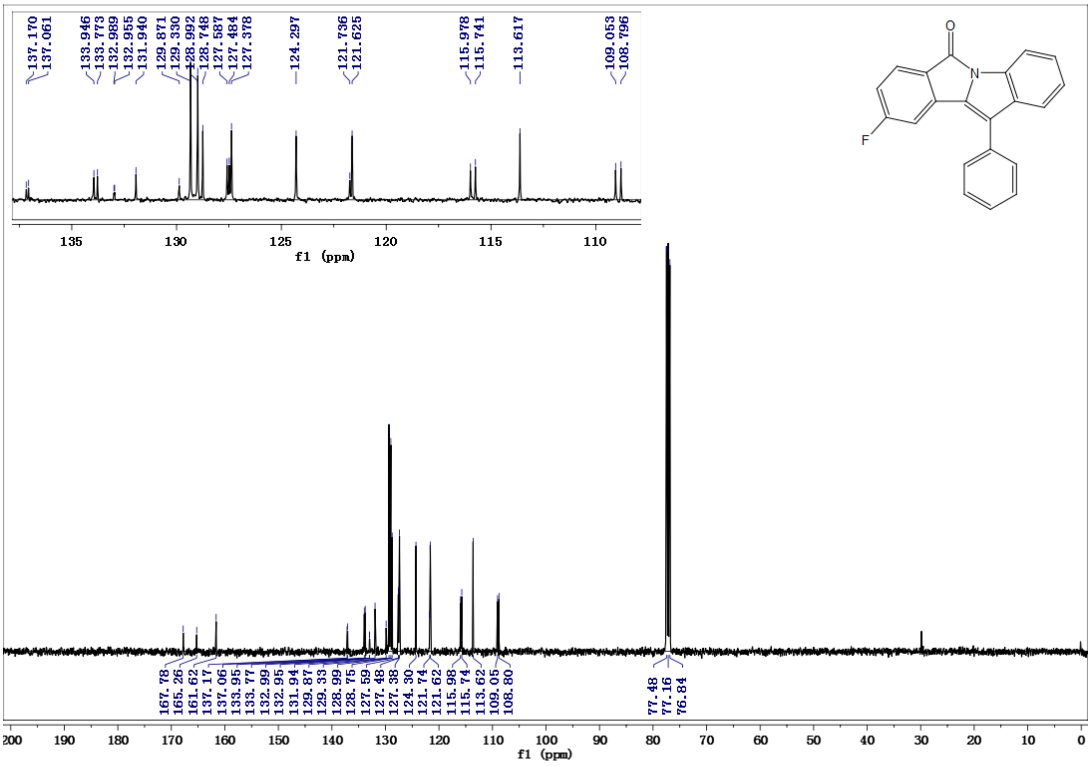

^{19}F NMR spectrum of **2caa**

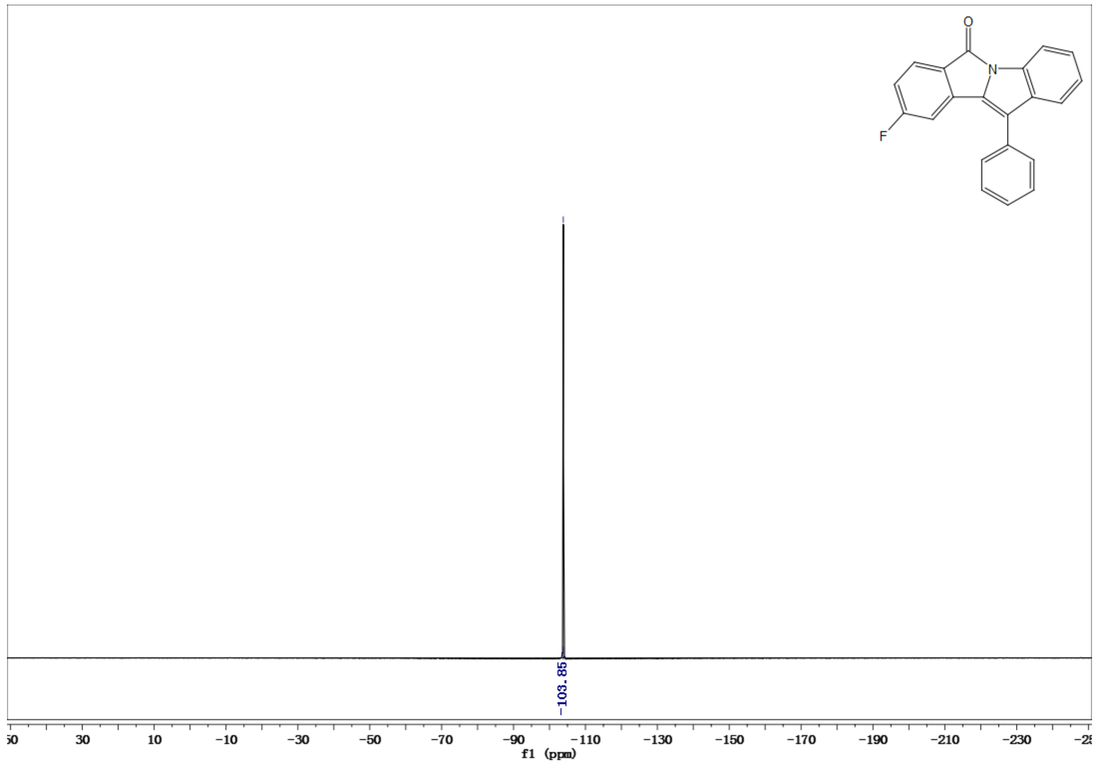

5. ^{1}H and ^{13}C NMR charts of the intermediates

^{1}H NMR spectrum of **2a**

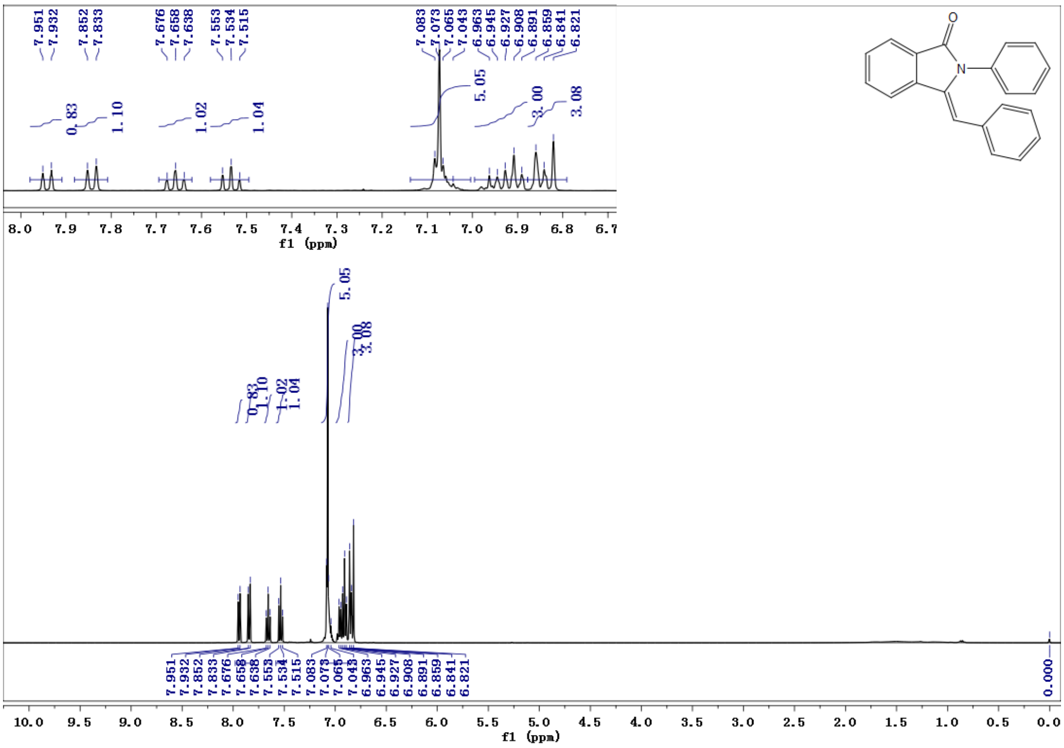

^{13}C NMR spectrum of **2a**

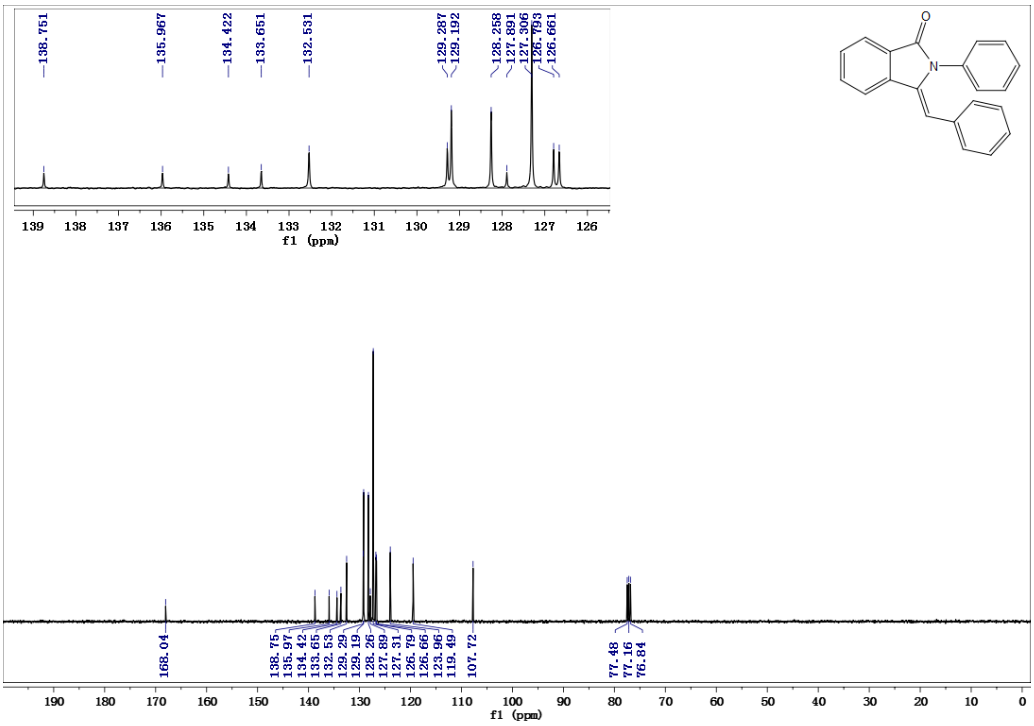

^{1}H NMR spectrum of **2d**

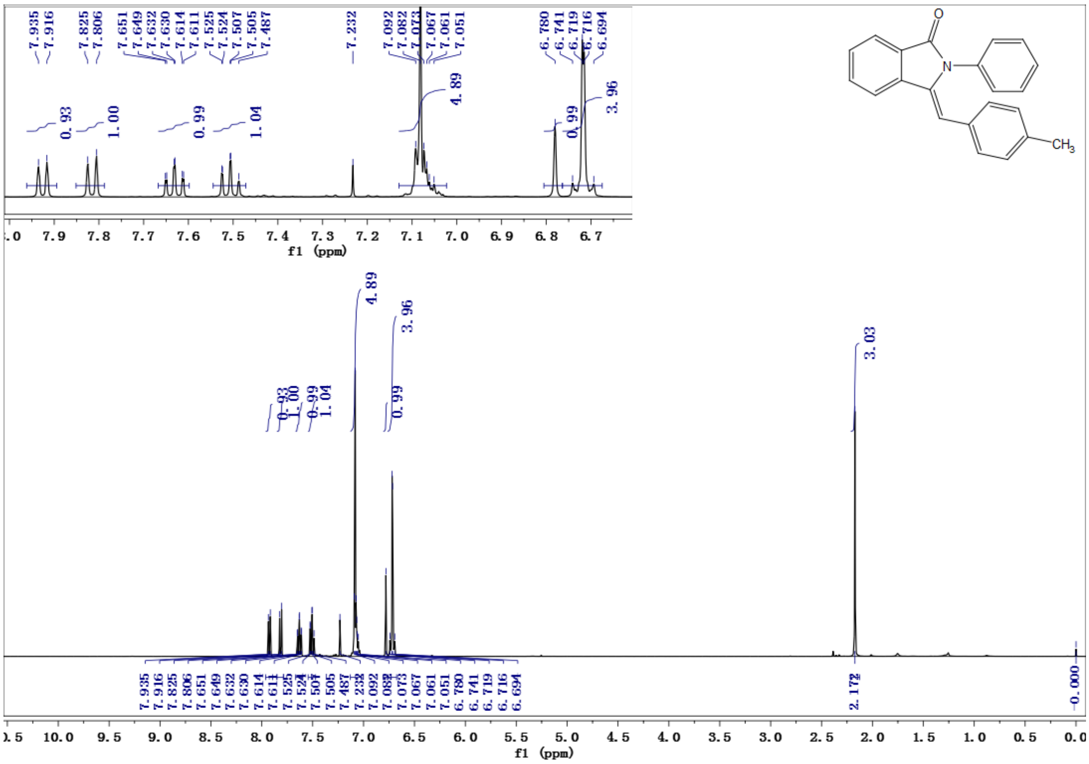

^{13}C NMR spectrum of **2d**

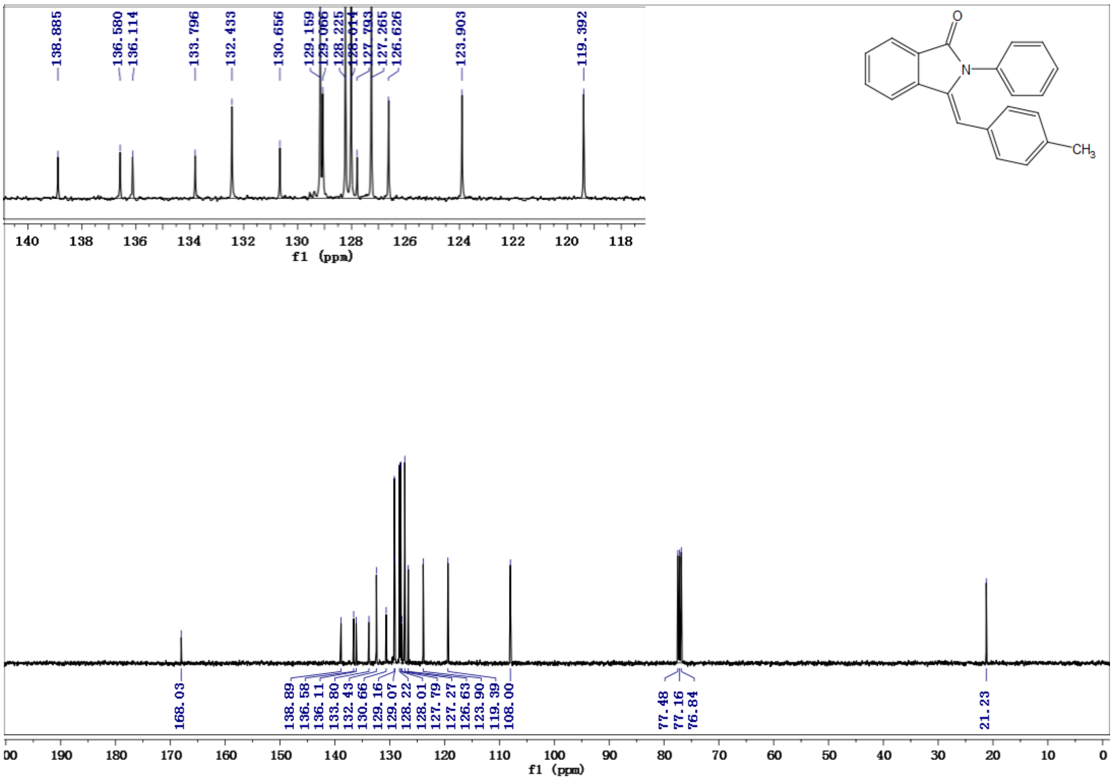

^{1}H NMR spectrum of **2d-*d*$_5$**

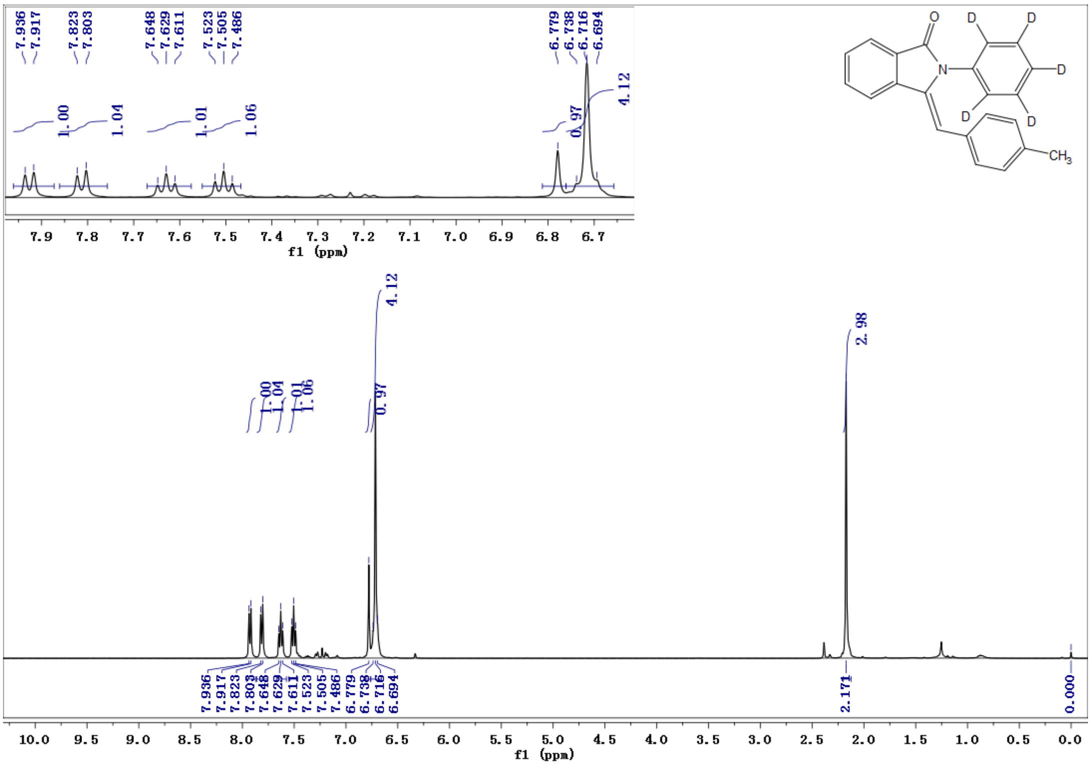

6. X-ray structural details of 2a

Crystal Structure of $C_{21}H_{15}NO$

The low temperature (173±2°K) single-crystal X-ray experiments were performed on a SuperNova diffractometer with Cu K_α radiation. Unit cell was obtained and refined by 2261 reflections with 5.1° < θ < 64.5°. No decay was observed in data collection. Raw intensities were corrected for Lorentz and polarization effects, and for absorption by empirical method. Direct phase determination yielded the positions of all non-hydrogen atoms. All non-hydrogen atoms were subjected to anisotropic refinement. All hydrogen atoms were generated geometrically with C-H bonds of 0.93 Å according to criteria described in the SHELXTL manual (Bruker, 1997). They were included in the refinement with $U_{iso}(H) = 1.2U_{eq}$ of their parent atoms. The final full-matric least-square refinement on F^2 converged with $R1 = 0.0311$ and $wR2 = 0.0711$ for 3825 observed reflections [$I \geq 2\sigma(I)$]. The final difference electron density map shows no features. Details of crystal parameters, data collection and structure refinement are given in Table 1.

Data collection was controlled by CrysAlisPro, Agilent Technologies, Version 1.171.36.32 (Oxford, 2013). Computations were performed using the SHELXTL NT ver. 5.10 program package (Bruker, 1997) on an IBM PC 586 computer. Analytic expressions of atomic scattering factors were employed, and anomalous dispersion corrections were incorporated (*International Tables for X-ray Crystallography*, 1989). Crystal drawings were produced with XP (Bruker, 1997).

Notes:

Bruker. (1997) SHELXTL. Structure Determination Programs, Version 5.10, Bruker AXS Inc.,6300 Enterprise Lane, Madison, WI 53719-1173, USA.

International Tables for X-ray Crystallography: (1989) Vol. C (Kluwer Academic Publishers, Dordrecht) Tables 4.2.6.8 and 6.1.1.4.

Oxford. (2013) CrysAlisPro, Agilent Technologies, Version 1.171.36.32, Oxford Diffraction Ltd., 68 Milton Park, Abingdon, Oxfordshire, OX14 4RX, UK.

Table 1. Details of Data Collection, Processing and Structure Refinement

Sample code	**2a**
Molecular formula	$C_{21}H_{15}NO$
Molecular weight	297.34
Color and habit	colorless prism
Crystal size	0.2 × 0.25 × 0.3 mm
Crystal system	orthorhombic
Space group	$P2_12_12_1$ (No. 19)
Unit cell parameters	a = 8.2130(3) Å α = 90.00°
	b = 9.3722(4) Å β = 90.00°
	c = 19.8868(8) Å γ = 90.00°
	V = 1530.76(11) $Å^3$ Z = 4 F(000) = 624
Density (calcd)	1.290 g/cm^3
Diffractometer	SuperNova, Dual, Cu at home/near, AtlasS2
Radiation	Cu K_α, λ = 1.54178 Å
Temperature	173±2K
Scan type	ω-scan
Data collection range	$-9 < h < 7$, $-10 < k < 9$, $-20 < l < 23$; $\theta_{max} = 65.2°$
Reflections measured	Total: 4399 Unique (n): 2354 Observed [I ≥ 2σ(I)]: 2119
Absorption coefficient	0.621 mm^{-1}
Minimum and maximum transmission	0.990, 1.000
No. of variables, p	209
Weighting scheme	$w = \dfrac{1}{\sigma^2(F_o^2) + (0.0322P)^2 + 0.0536P}$ $P = (F_o^2 + 2F_c^2)/3$
$R1 = \dfrac{\Sigma\lvert\lvert F_o\rvert - \lvert F_c\rvert\rvert}{\Sigma\lvert F_o\rvert}$ (for all reflections)	0.0358 0.0311 (for observed data)
$wR2 = \sqrt{\dfrac{\Sigma[w(F_o^2 - F_c^2)^2]}{\Sigma w(F_o^2)^2}}$ (for all reflections)	0.0743 0.0711 (for observed data)
Goof = S = $\sqrt{\dfrac{\Sigma[w(F_o^2 - F_c^2)^2]}{n - p}}$	1.070
Largest and mean Δ/σ	0.000, 0.000
Residual extrema in final difference map	-0.094 to 0.104 e $Å^{-3}$

Table 2. Atomic coordinates and equivalent isotropic temperature factors* (Å^2)

Atoms	x	y	z	$U_{eq.}$
O(1)	0.6909(2)	0.4025(2)	0.88689(9)	0.0757(6)
N(1)	0.8626(2)	0.3857(2)	0.79528(9)	0.0469(4)
C(1)	0.8279(3)	0.3934(3)	0.86377(12)	0.0519(6)
C(2)	0.9856(3)	0.3871(2)	0.89838(11)	0.0473(5)
C(3)	1.0216(3)	0.3953(3)	0.96639(12)	0.0579(6)
C(4)	1.1826(3)	0.3877(3)	0.98520(12)	0.0620(7)
C(5)	1.3041(3)	0.3732(3)	0.93756(13)	0.0592(6)
C(6)	1.2684(3)	0.3652(2)	0.86988(12)	0.0512(6)
C(7)	1.1068(3)	0.3723(2)	0.85063(11)	0.0434(5)
C(8)	1.0322(2)	0.3719(2)	0.78355(11)	0.0418(5)
C(9)	1.1129(3)	0.3627(2)	0.72557(11)	0.0459(5)
C(10)	1.0558(2)	0.3754(2)	0.65584(11)	0.0435(5)
C(11)	1.0935(3)	0.2713(3)	0.60880(13)	0.0562(6)
C(12)	1.0375(3)	0.2799(3)	0.54408(13)	0.0667(7)
C(13)	0.9432(3)	0.3934(3)	0.52435(13)	0.0620(7)
C(14)	0.9100(3)	0.5006(3)	0.56946(13)	0.0588(6)
C(15)	0.9658(3)	0.4915(2)	0.63435(11)	0.0502(6)
C(16)	0.7376(2)	0.3784(2)	0.74535(11)	0.0453(5)
C(17)	0.7203(3)	0.2577(3)	0.70637(12)	0.0536(6)
C(18)	0.6069(3)	0.2558(3)	0.65509(13)	0.0634(7)
C(19)	0.5089(3)	0.3722(3)	0.64366(13)	0.0653(7)
C(20)	0.5227(3)	0.4904(3)	0.68434(14)	0.0670(7)
C(21)	0.6364(3)	0.4937(3)	0.73526(12)	0.0563(6)

*$U_{eq.}$ defined as one third of the trace of the orthogonalized **U** tensor.

Table 3. Bond lengths (Å) and bond angles (º)

O(1)-C(1)	1.218(3)	C(9)-C(10)	1.469(3)
N(1)-C(1)	1.393(3)	C(10)-C(15)	1.383(3)
N(1)-C(8)	1.418(3)	C(10)-C(11)	1.386(3)
N(1)-C(16)	1.430(3)	C(11)-C(12)	1.369(4)
C(1)-C(2)	1.468(3)	C(12)-C(13)	1.373(3)
C(2)-C(7)	1.383(3)	C(13)-C(14)	1.374(4)
C(2)-C(3)	1.387(3)	C(14)-C(15)	1.372(3)
C(3)-C(4)	1.376(3)	C(16)-C(21)	1.378(3)
C(4)-C(5)	1.383(3)	C(16)-C(17)	1.379(3)
C(5)-C(6)	1.380(3)	C(17)-C(18)	1.381(3)
C(6)-C(7)	1.383(3)	C(18)-C(19)	1.375(4)
C(7)-C(8)	1.468(3)	C(19)-C(20)	1.376(4)
C(8)-C(9)	1.333(3)	C(20)-C(21)	1.378(3)
C(1)-N(1)-C(8)	111.50(18)	N(1)-C(8)-C(7)	105.08(18)
C(1)-N(1)-C(16)	122.31(17)	C(8)-C(9)-C(10)	130.77(19)
C(8)-N(1)-C(16)	125.91(17)	C(15)-C(10)-C(11)	117.7(2)
O(1)-C(1)-N(1)	124.2(2)	C(15)-C(10)-C(9)	121.7(2)
O(1)-C(1)-C(2)	129.8(2)	C(11)-C(10)-C(9)	120.6(2)
N(1)-C(1)-C(2)	106.00(18)	C(12)-C(11)-C(10)	121.2(2)
C(7)-C(2)-C(3)	121.5(2)	C(11)-C(12)-C(13)	120.3(2)
C(7)-C(2)-C(1)	108.50(19)	C(12)-C(13)-C(14)	119.4(2)
C(3)-C(2)-C(1)	130.0(2)	C(15)-C(14)-C(13)	120.2(2)
C(4)-C(3)-C(2)	117.8(2)	C(14)-C(15)-C(10)	121.2(2)
C(3)-C(4)-C(5)	120.8(2)	C(21)-C(16)-C(17)	120.0(2)
C(6)-C(5)-C(4)	121.4(2)	C(21)-C(16)-N(1)	119.8(2)
C(5)-C(6)-C(7)	118.1(2)	C(17)-C(16)-N(1)	120.3(2)
C(2)-C(7)-C(6)	120.4(2)	C(16)-C(17)-C(18)	119.7(2)
C(2)-C(7)-C(8)	108.91(18)	C(19)-C(18)-C(17)	120.5(2)
C(6)-C(7)-C(8)	130.7(2)	C(18)-C(19)-C(20)	119.6(2)
C(9)-C(8)-N(1)	129.5(2)	C(19)-C(20)-C(21)	120.4(2)
C(9)-C(8)-C(7)	125.39(18)	C(20)-C(21)-C(16)	119.9(2)

Table 4. Anisotropic thermal parameters* (Å^2)

Atoms	U_{11}	U_{22}	U_{33}	U_{23}	U_{13}	U_{12}
O(1)	0.0523(10)	0.1152(15)	0.0597(11)	-0.0060(11)	0.0144(9)	0.0028(11)
N(1)	0.0386(9)	0.0586(11)	0.0433(10)	-0.0036(9)	0.0034(8)	-0.0006(9)
C(1)	0.0483(13)	0.0587(14)	0.0488(13)	-0.0030(12)	0.0079(12)	-0.0002(12)
C(2)	0.0504(12)	0.0448(12)	0.0465(12)	-0.0004(11)	0.0038(11)	-0.0034(11)
C(3)	0.0676(15)	0.0601(15)	0.0459(13)	-0.0035(11)	0.0062(13)	0.0004(13)
C(4)	0.0767(17)	0.0614(15)	0.0478(14)	-0.0027(13)	-0.0117(14)	-0.0034(15)
C(5)	0.0582(14)	0.0586(14)	0.0607(16)	0.0010(13)	-0.0124(13)	-0.0040(13)
C(6)	0.0463(12)	0.0521(13)	0.0552(14)	0.0007(12)	-0.0008(11)	-0.0033(11)
C(7)	0.0466(11)	0.0379(12)	0.0458(13)	0.0003(11)	0.0002(10)	-0.0029(10)
C(8)	0.0396(10)	0.0385(11)	0.0472(12)	-0.0010(11)	0.0026(10)	-0.0013(10)
C(9)	0.0410(11)	0.0480(12)	0.0487(13)	0.0014(11)	0.0039(10)	0.0018(10)
C(10)	0.0387(10)	0.0461(13)	0.0456(12)	0.0023(11)	0.0057(10)	-0.0019(11)
C(11)	0.0624(14)	0.0523(14)	0.0540(14)	-0.0010(12)	0.0017(13)	0.0104(12)
C(12)	0.0822(18)	0.0667(17)	0.0512(15)	-0.0104(13)	-0.0001(15)	0.0036(15)
C(13)	0.0615(15)	0.0746(18)	0.0498(14)	0.0118(14)	-0.0067(13)	-0.0097(15)
C(14)	0.0560(14)	0.0557(15)	0.0646(15)	0.0187(14)	0.0033(13)	0.0038(12)
C(15)	0.0513(12)	0.0461(13)	0.0531(14)	0.0032(11)	0.0092(12)	0.0013(11)
C(16)	0.0369(10)	0.0528(13)	0.0463(12)	-0.0006(11)	0.0061(10)	-0.0044(11)
C(17)	0.0541(13)	0.0503(13)	0.0565(15)	-0.0007(12)	0.0007(13)	-0.0063(11)
C(18)	0.0615(15)	0.0701(17)	0.0586(16)	-0.0086(14)	-0.0009(14)	-0.0159(15)
C(19)	0.0460(13)	0.093(2)	0.0571(15)	0.0015(16)	-0.0038(12)	-0.0073(15)
C(20)	0.0455(13)	0.0824(19)	0.0733(17)	0.0013(16)	0.0012(13)	0.0110(14)
C(21)	0.0457(12)	0.0605(14)	0.0627(15)	-0.0086(13)	0.0026(12)	0.0048(12)

*The exponent takes the form: $-2\pi^2\Sigma\Sigma U_{ij}h_ih_j\mathbf{a}_i^*\mathbf{a}_j^*$

Table 5. Coordinates and isotropic temperature factors* (Å^2) for H atoms

Atoms	x	y	z	$U_{eq.}$
H(3)	0.9395	0.4056	0.9983	0.069
H(4)	1.2100	0.3924	1.0305	0.074
H(5)	1.4121	0.3687	0.9514	0.071
H(6)	1.3507	0.3554	0.8381	0.061
H(9)	1.2238	0.3454	0.7300	0.055
H(11)	1.1581	0.1943	0.6214	0.067
H(12)	1.0634	0.2087	0.5134	0.080
H(13)	0.9020	0.3977	0.4808	0.074
H(14)	0.8496	0.5795	0.5560	0.071
H(15)	0.9428	0.5648	0.6644	0.060
H(17)	0.7846	0.1779	0.7146	0.064
H(18)	0.5969	0.1753	0.6281	0.076
H(19)	0.4338	0.3711	0.6087	0.078
H(20)	0.4548	0.5684	0.6774	0.080
H(21)	0.6449	0.5736	0.7627	0.068

*The exponent takes the form: $-8\pi^2 U \sin^2\theta/\lambda^2$

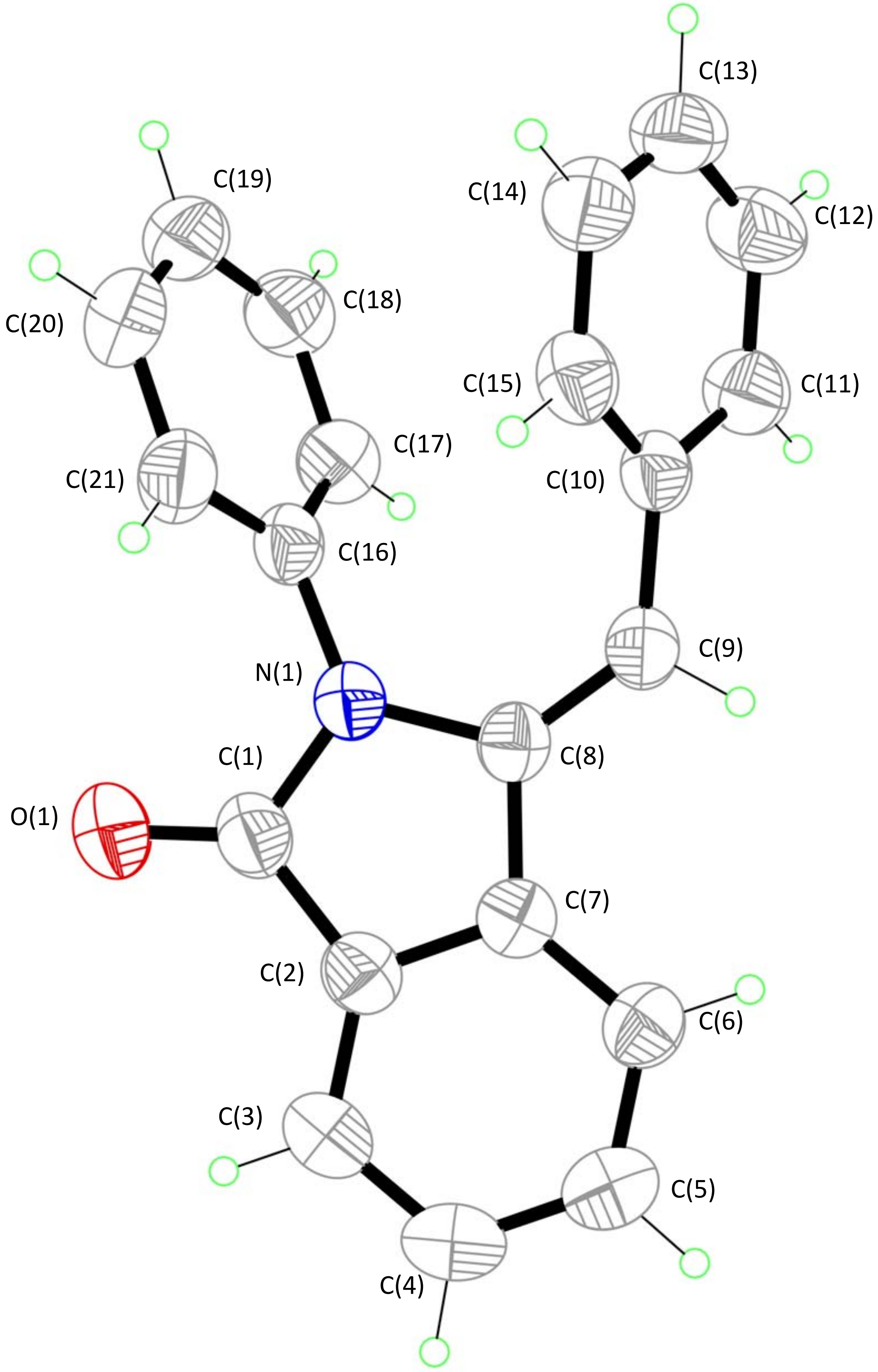

ORTEP drawing of $C_{21}H_{15}NO$ with 50% probability ellipsoids, showing the atomic numbering scheme.

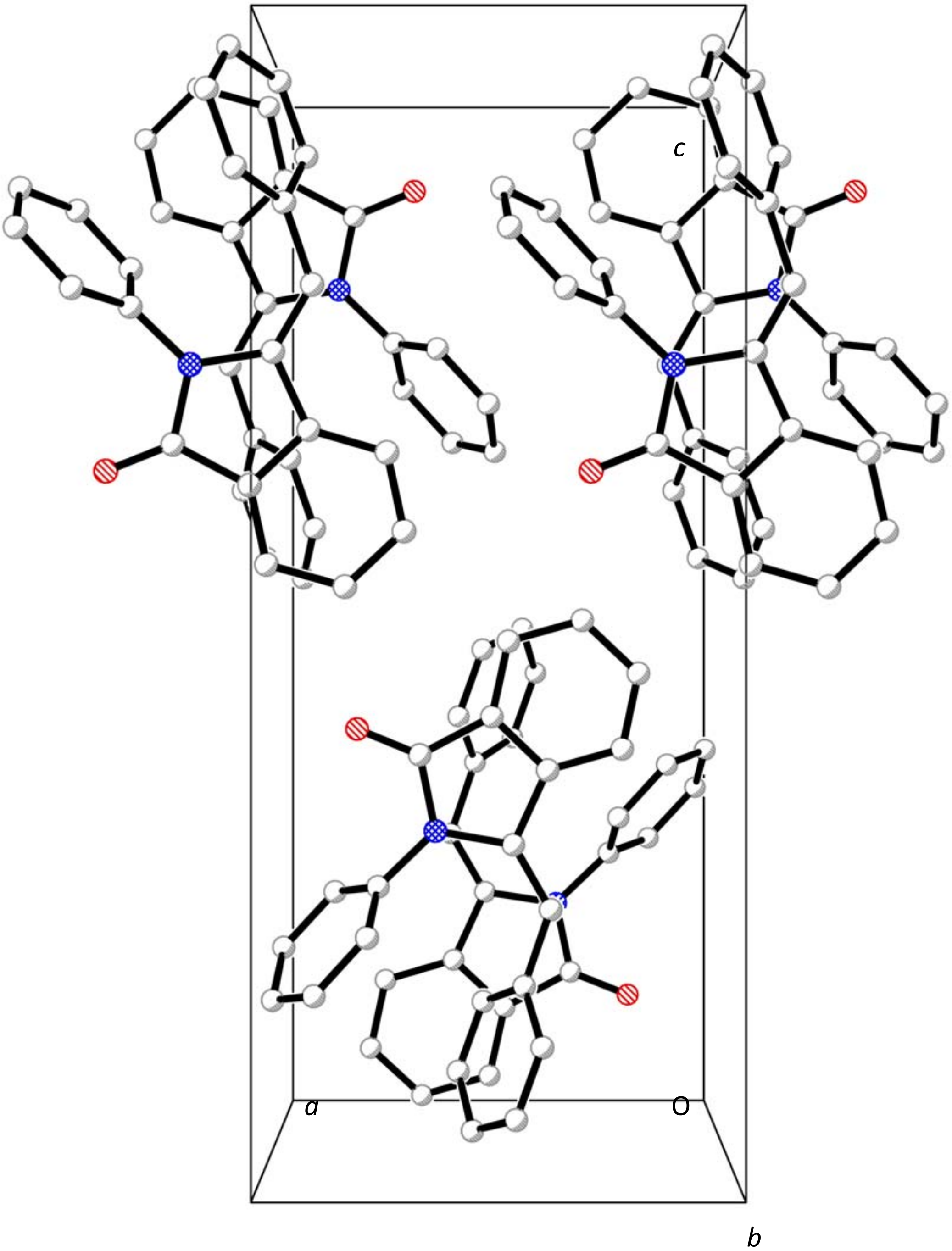

A packing view along the *b* direction
